# Supplementary material for: Effect of genetically predicted sclerostin on cardiovascular biomarkers, risk factors, and disease outcomes
Source: Nat Commun. 2024 Nov 13;15:9832. doi: 10.1038/s41467-024-53623-5 (PMC11561231; doi:10.1038/s41467-024-53623-5)
Supplement: Supplementary file 1 — Supplementary information [file 41467_2024_53623_MOESM1_ESM.pdf]

Supplementary Figures and Tables

Supplementary Table 1. Results from the fixed-effects and random effects meta-analysis of the instruments. Sun et al. used simple linear regression using an additive genetic model for the test statistic. Ferkingstad et al. used linear mixed model implemented in BOLT-LMM for the test statistic. Pietzner et al. used BGENIE to perform a linear association test. Note: EA = Effect allele, OA = Other allele, SE = Standard error.

SNP	EA	OA	Beta			SE			Effect		SE		P-Value		Heterogeneity			Sample size
			Sun et al. 2018	Ferkingstad et al., 2021	Pietzner et al., 2021	Sun et al. 2018	Ferkingstad et al., 2021	Pietzner et al., 2021	Fixed	Random	Fixed	Random	Fixed	Random	I^2	Q statistic	Q P-Value	
rs7220711	A	G	0.0225	0.0323	0.0661	0.0255	0.00863	0.0125	0.042	0.04303	0.006842	0.013383	1.11E-09	0.00131	0.640486	5.563072	0.061943	49,372
rs66838809	A	G	-0.0833	-0.0462	-0.12	0.0485	0.0137	0.0232	-0.07	-0.08054	0.011478	0.028263	7.76E-09	0.00439	0.737024	7.605257	0.022312	49,372

Supplementary Table 2. Linkage disequilibrium correlation matrix of the instruments. The matrix was calculated using 1000 genomes LD European reference panel. Notice that the values correspond to r, not r^2.

SNP	rs7220711 (G-A)	rs66838809 (A-G)
**rs7220711 (G-A)**	1	0.376
**rs66838809 (A-G)**	0.376	1

Supplementary Table 3. Results of the Mendelian randomisation of the positive control outcomes. MR estimates have been calculated using generalised inverse variance weighted method.

Outcome	N instruments	Type of estimate	MR estimate	Standard error	P value	GWAS ID
Heel bone mineral density	2	Beta [SD change/SD decrease in sclerostin levels]	1.00187062	0.04024844	9.05E-137	https://ftp.ebi.ac.uk/pub/databases/gwas/summary_statistics/GCST006001-GCST007000/GCST006979/
Hip fracture	2	OR / SD decrease in sclerostin levels	0.15877783	0.330056003	2.46725E-08	http://ftp.ebi.ac.uk/pub/databases/gwas/summary_statistics/GCST90161001-GCST90162000/GCST90161240/

Supplementary Table 4. Results of the Mendelian randomisation of the GWAS outcomes. MR estimates have been calculated using generalised inverse variance weighted method.

Outcome	N instruments	Type of estimate	MR estimate	Standard error	P value	GWAS ID
LDL cholesterol	2	Beta [mg/dL change/SD decrease in sclerostin levels]	0.01908919	0.03039105	0.52992621	https://csg.sph.umich.edu/willer/public/glgc-lipids2021/
HDL cholesterol	2	Beta [mg/dL change/SD decrease in sclerostin levels]	-0.1455347	0.03013333	1.37E-06	https://csg.sph.umich.edu/willer/public/glgc-lipids2021/
Fasting glucose	2	Beta [mmol/L change/SD decrease in sclerostin levels]	0.02641789	0.03965886	0.50532907	https://magicinvestigators.org/downloads/
HbA1c	2	Beta [% change/SD decrease in sclerostin levels]	0.02104062	0.02902738	0.46854154	https://magicinvestigators.org/downloads/
Coronary artery disease	2	OR / SD decrease in sclerostin levels	1.24958769	0.10954944	0.04196071	https://ftp.ebi.ac.uk/pub/databases/gwas/summary_statistics/GCST90132001-GCST90133000/GCST90132314/
Myocardial infarction	2	OR / SD decrease in sclerostin levels	1.35466428	0.16583496	0.06718173	https://ftp.ebi.ac.uk/pub/databases/gwas/summary_statistics/GCST011001-GCST012000/GCST011365/harmonised/
Ischaemic stroke	2	OR / SD decrease in sclerostin levels	1.18979215	0.15578975	0.2646495	https://ftp.ebi.ac.uk/pub/databases/gwas/summary_statistics/GCST90104001-GCST90105000/GCST90104540/
Hypertension	2	OR / SD decrease in sclerostin levels	1.0264745	0.01958368	0.18211216	ukb-b-14057
Type 2 diabetes mellitus	2	OR / SD decrease in sclerostin levels	1.44983479	0.1384198	0.00728558	https://www.ebi.ac.uk/gwas/studies/GCST90132184

Supplementary Table 5. Study information of the UK Biobank cohorts.

Outcome	Type of outcome	N	Age (SD)	Male (%)	Mean (SD) /Cases (%)	Mean followup
Whole cohort	-	276,172	57 (8)	128,946 (47)	-	-
Cholesterol	Continuous	263,285	57 (8)	123,011 (47)	5.72 (1.14)	-
LDL cholesterol	Continuous	262,794	57 (8)	122,752 (47)	3.57 (0.87)	-
HDL cholesterol	Continuous	241,065	57 (8)	113,574 (47)	1.45 (0.38)	-
Triglycerides	Continuous	263,067	57 (8)	122,877 (47)	1.75 (1.02)	-
Apolipoprotein-A	Continuous	239,707	57 (8)	113,397 (47)	1.54 (0.27)	-
Apolipoprotein-B	Continuous	261,986	57 (8)	122,208 (47)	1.03 (0.24)	-
C-Reactive protein	Continuous	262,728	57 (8)	122,697 (47)	2.56 (4.35)	-
Lipoprotein (a)	Continuous	209,333	57 (8)	974,06 (47)	44.07 (49.44)	-
Glucose	Continuous	240,909	57 (8)	113,502 (47)	5.12 (1.21)	-
HbA1c	Continuous	263,079	57 (8)	122,845 (47)	35.92 (6.45)	-
Coronary artery disease	Binary	276,172	57 (8)	128,946 (47)	16,233 (6)	-
Myocardial infarction	Binary	276,172	57 (8)	128,946 (47)	10,861 (4)	-
Ischaemic stroke	Binary	276,172	57 (8)	128,946 (47)	3,830 (1)	-
Hypertension	Binary	276,172	57 (8)	128,946 (47)	98,040 (35)	-
Type 2 diabetes	Binary	276,172	57 (8)	128,946 (47)	21,664 (8)	-
Coronary artery disease	Survival	276,172	57 (8)	128,946 (47)	16,233 (6)	68 (8)
Myocardial infarction	Survival	276,065	57 (8)	128,876 (47)	10,754 (4)	68 (8)
Ischaemic stroke	Survival	276,172	57 (8)	128,946 (47)	3,830 (1)	68 (8)
Hypertension	Survival	271,359	57 (8)	127,354 (47)	93,227 (34)	63 (11)
Type 2 diabetes	Survival	266,268	57 (8)	122,592 (46)	11,760 (4)	68 (8)

Supplementary Table 6. Results of the Mendelian randomisation for UK Biobank outcomes.

Outcome	Instruments	Type of estimate	MR estimate	Standard error	P value
Cholesterol	2	Beta [SD change/SD decrease in sclerostin levels]	0.04351909	0.06003166	0.46849154
LDL cholesterol	2	Beta [SD change/SD decrease in sclerostin levels]	0.06030358	0.06081673	0.32141108
HDL cholesterol	2	Beta [SD change/SD decrease in sclerostin levels]	-0.1307288	0.05793801	0.02404829
Apolipoprotein-A	2	Beta [SD change/SD decrease in sclerostin levels]	-0.0782697	0.05897189	0.18443007
Apolipoprotein-B	2	Beta [SD change/SD decrease in sclerostin levels]	0.11891979	0.06111597	0.05167812
Lipoprotein (a)	2	Beta [SD change/SD decrease in sclerostin levels]	-0.0356529	0.06837771	0.60208033
C-Reactive protein	2	Beta [SD change/SD decrease in sclerostin levels]	0.01955029	0.06100932	0.7486291
Triglycerides	2	Beta [SD change/SD decrease in sclerostin levels]	0.26206403	0.05966256	1.12E-05
HbA1c	2	Beta [SD change/SD decrease in sclerostin levels]	-0.0045441	0.06088411	0.94050497
Glucose	2	Beta [SD change/SD decrease in sclerostin levels]	0.08939238	0.06360014	0.15986151
Coronary artery disease	2	OR / SD decrease in sclerostin levels	1.84897728	0.2572956	1.69E-02
Myocardial infarction	2	OR / SD decrease in sclerostin levels	1.37578563	0.31152161	0.30579464
Ischaemic stroke	2	OR / SD decrease in sclerostin levels	1.28494794	0.50747985	0.62127395
Hypertension	2	OR / SD decrease in sclerostin levels	1.18101945	0.13033265	0.20175612
Type 2 diabetes	2	OR / SD decrease in sclerostin levels	1.62002558	0.22238731	3.01E-02
Coronary artery disease	2	HR / SD decrease in sclerostin levels	1.770027	0.2437155	0.01913579
Myocardial infarction	2	HR / SD decrease in sclerostin levels	1.369607	0.30190278	0.29750167
Ischaemic stroke	2	HR / SD decrease in sclerostin levels	1.285523	0.50198067	0.6168292
Hypertension	2	HR / SD decrease in sclerostin levels	1.120222	0.10221314	0.26670209
Type 2 diabetes	2	HR / SD decrease in sclerostin levels	1.668812	0.28629863	0.07365778

Supplementary Table 7. Study information of the cohorts used for the GWAS meta-analysis of sclerostin.

**Article reference**	**Study**	**Outcome**	**N**	**Age (SD)**	**% Female**	**Study population**
**Sun et al., 2018**	INTERVAL study	Sclerostin	3,301	43 (14)	49	European (England)
**Ferkingstad et al., 2021**	deCODE genetics	Sclerostin	35,559	55 (17)	57	European (Iceland)
**Pietzner et al., 2021**	Fenland study	Sclerostin	10,708	49 (7)	53	European (England)

Supplementary Table 8. Study information of the cohorts used in the published GWAS summary statistic of the outcomes. Note: LDL cholesterol, HDL cholesterol, and HbA1c mean and standard values were not reported by the original publication and were estimated as weighted (by the sample sizes) average of the means reported in the contributing studies. Glucose mean value could not be estimated.

Article reference	Study	Outcome	N	Mean (SD)<br>N cases (%)	Study population
**Morris et al., 2019**	UK Biobank	Heel bone mineral density	426,824	0.54 (0.12) [g/cm^2]	European
**Nethander et al., 2022**	Meta-analysis	Hip fracture	735,354	11,516 (2)	European
**Aragam et al., 2022**	Meta-analysis	Coronary artery disease	1,165,690	181,522 (16)	European
**Hartiala et al., 2021**	Meta-analysis	Myocardial infarction	~639,000	~62,000 (10)	European
**Mishra et al., 2022**	Meta-analysis	Ischaemic stroke	1,847,683	612,875 (33)	European
**Elsworth et al., 2018**	UK Biobank	Hypertension	462,933	119,731 (26)	European
**Mahajan et al., 2022**	Meta-analysis	Type II diabetes mellitus	933,970	80,154 (9)	European
**Graham et al., 2021**	Meta-analysis	LDL-Cholesterol	1,228,508	131 [mg/dL]	European
**Graham et al., 2021**	Meta-analysis	HDL-Cholesterol	1,244,439	51.9 [mg/dL]	European
**Chen et al., 2021**	Meta-analysis	Fasting glucose	178,455	-	European
**Chen et al., 2021**	Meta-analysis	HbA1c	132,400	5.23 [mmol/l]	European

Supplementary Table 9. Sensitivity analyses results. Note: gIVW = generalised inverse variance weighted method, OR = Odds ratio, SD = Standard deviation, MR = Mendelian randomisation,.

Sensitivity analysis	Outcome	Source of outcome data	r^2 threshold	N instruments	N outcome	Type of MR estimate (units)	MR method	MR estimate	S.E of the MR estimate	P-Value of the MR estimate
Step-wise pruning	Heel bone mineral density	GWAS	$r^2 \leq 0.001$	1	426,824	Beta [SD change/SD decrease in sclerostin levels]	Wald ratio	0.932381	0.04582019	4.77E-92
			$r^2 \leq 0.1$	1	426,824	Beta [SD change/SD decrease in sclerostin levels]	gIVW	0.932381	0.04582019	4.77E-92
			$r^2 \leq 0.5$	2	426,824	Beta [SD change/SD decrease in sclerostin levels]	gIVW	1.00187063	0.04024844	9.05E-137
			$r^2 \leq 0.8$	6	426,824	Beta [SD change/SD decrease in sclerostin levels]	gIVW	0.53032519	0.19761409	0.00728254
Step-wise pruning	Hip fracture	GWAS	$r^2 \leq 0.001$	1	735,354	OR / SD decrease in sclerostin levels	Wald ratio	0.18412271	0.35952255	2.52E-06
			$r^2 \leq 0.1$	1	735,354	OR / SD decrease in sclerostin levels	gIVW	0.18412271	0.35952255	2.52E-06
			$r^2 \leq 0.5$	2	735,354	OR / SD decrease in sclerostin levels	gIVW	0.15877783	0.330056	2.47E-08
			$r^2 \leq 0.8$	6	735,354	OR / SD decrease in sclerostin levels	gIVW	0.37250533	0.39046181	0.01143663
Step-wise pruning	LDL cholesterol	GWAS	$r^2 \leq 0.001$	1	1,228,508	Beta [mg/dL change/SD decrease in sclerostin levels]	Wald ratio	0.02570922	0.0342136	0.45239234
			$r^2 \leq 0.1$	1	1,228,508	Beta [mg/dL change/SD decrease in sclerostin levels]	gIVW	0.02570922	0.0342136	0.45239234
			$r^2 \leq 0.5$	2	1,228,508	Beta [mg/dL change/SD decrease in sclerostin levels]	gIVW	0.01908919	0.03039105	0.52992621
			$r^2 \leq 0.8$	6	1,228,508	Beta [mg/dL change/SD decrease in sclerostin levels]	gIVW	-0.02232495	0.02297269	0.33114821
Step-wise pruning	HDL cholesterol	GWAS	$r^2 \leq 0.001$	1	1,244,439	Beta [mg/dL change/SD decrease in sclerostin levels]	Wald ratio	-0.166144	0.0339128	9.63E-07
			$r^2 \leq 0.1$	1	1,244,439	Beta [mg/dL change/SD decrease in sclerostin levels]	gIVW	-0.166144	0.0339128	9.63E-07
			$r^2 \leq 0.5$	2	1,244,439	Beta [mg/dL change/SD decrease in sclerostin levels]	gIVW	-0.14553469	0.03013333	1.37E-06
			$r^2 \leq 0.8$	6	1,244,439	Beta [mg/dL change/SD decrease in sclerostin levels]	gIVW	-0.15377985	0.08674871	0.07627765
Step-wise pruning	Fasting glucose	GWAS	$r^2 \leq 0.001$	1	178,455	Beta [mmol/dL change/SD decrease in sclerostin levels]	Wald ratio	0	0.04314271	1
			$r^2 \leq 0.1$	1	178,455	Beta [mmol/dL change/SD decrease in sclerostin levels]	gIVW	0	0.04314271	1
			$r^2 \leq 0.5$	2	178,455	Beta [mmol/dL change/SD decrease in sclerostin levels]	gIVW	0.02641789	0.03965886	0.50532907
			$r^2 \leq 0.8$	6	178,455	Beta [mmol/dL change/SD decrease in sclerostin levels]	gIVW	0.04282551	0.03192605	0.17979161

Step-wise pruning	HbA1c	GWAS	$r^2 \leq 0.001$	1	132,400	Beta [% change/SD decrease in sclerostin levels]	Wald ratio	0.02157135	0.03115862	0.48874412
			$r^2 \leq 0.1$	1	132,400	Beta [% change/SD decrease in sclerostin levels]	gIVW	0.02157135	0.03115862	0.48874412
			$r^2 \leq 0.5$	2	132,400	Beta [% change/SD decrease in sclerostin levels]	gIVW	0.02104062	0.02902738	0.46854154
			$r^2 \leq 0.8$	6	132,400	Beta [% change/SD decrease in sclerostin levels]	gIVW	0.00092934	0.02037851	0.96362612
Step-wise pruning	Coronary artery disease	GWAS	$r^2 \leq 0.001$	1	1,165,690	OR / SD decrease in sclerostin levels	Wald ratio	1.30240886	0.12446919	0.03377579
			$r^2 \leq 0.1$	1	1,165,690	OR / SD decrease in sclerostin levels	gIVW	1.30240886	0.12446919	0.03377579
			$r^2 \leq 0.5$	2	1,165,690	OR / SD decrease in sclerostin levels	gIVW	1.24958769	0.10954944	0.04196071
			$r^2 \leq 0.8$	6	1,165,690	OR / SD decrease in sclerostin levels	gIVW	1.05645197	0.09736719	0.57274715
Step-wise pruning	Myocardial infarction	GWAS	$r^2 \leq 0.001$	1	~639,000	OR / SD decrease in sclerostin levels	Wald ratio	1.28167323	0.18251762	0.17392968
			$r^2 \leq 0.1$	1	~639,000	OR / SD decrease in sclerostin levels	gIVW	1.28167323	0.18251762	0.17392968
			$r^2 \leq 0.5$	2	~639,000	OR / SD decrease in sclerostin levels	gIVW	1.35466428	0.16583496	0.06718173
			$r^2 \leq 0.8$	6	~639,000	OR / SD decrease in sclerostin levels	gIVW	1.21611639	0.10627912	0.06561792
Step-wise pruning	Ischaemic stroke	GWAS	$r^2 \leq 0.001$	1	1,847,683	OR / SD decrease in sclerostin levels	Wald ratio	1.32369569	0.17017401	0.0993758
			$r^2 \leq 0.1$	1	1,847,683	OR / SD decrease in sclerostin levels	gIVW	1.32369569	0.17017401	0.0993758
			$r^2 \leq 0.5$	2	1,847,683	OR / SD decrease in sclerostin levels	gIVW	1.18979215	0.15578975	0.2646495
			$r^2 \leq 0.8$	6	1,847,683	OR / SD decrease in sclerostin levels	gIVW	1.24937878	0.18716618	0.23421717
Step-wise pruning	Hypertension	GWAS	$r^2 \leq 0.001$	1	462,933	OR / SD decrease in sclerostin levels	Wald ratio	1.01383256	0.02224019	0.53677305
			$r^2 \leq 0.1$	1	462,933	OR / SD decrease in sclerostin levels	gIVW	1.01383256	0.02224019	0.53677305
			$r^2 \leq 0.5$	2	462,933	OR / SD decrease in sclerostin levels	gIVW	1.0264745	0.01958368	0.18211216
			$r^2 \leq 0.8$	6	462,933	OR / SD decrease in sclerostin levels	gIVW	1.00711378	0.0124633	0.56952081
Step-wise pruning	Type 2 diabetes	GWAS	$r^2 \leq 0.001$	1	933,970	OR / SD decrease in sclerostin levels	Wald ratio	1.56549925	0.15818992	0.00460653
			$r^2 \leq 0.1$	1	933,970	OR / SD decrease in sclerostin levels	gIVW	1.56549925	0.15818992	0.00460653
			$r^2 \leq 0.5$	2	933,970	OR / SD decrease in sclerostin levels	gIVW	1.44983479	0.1384198	0.00728559
			$r^2 \leq 0.8$	6	933,970	OR / SD decrease in sclerostin levels	gIVW	1.18970371	0.10298299	0.09165567
Step-wise pruning	Cholesterol	UK Biobank	$r^2 \leq 0.001$	1	263,285	Beta [SD change/SD decrease in sclerostin levels]	Wald ratio	0.04102183	0.06778907	0.54508646
			$r^2 \leq 0.1$	1	263,285	Beta [SD change/SD decrease in sclerostin levels]	gIVW	0.04102183	0.06778907	0.54508646
			$r^2 \leq 0.5$	2	263,285	Beta [SD change/SD decrease in sclerostin levels]	gIVW	0.04556569	0.05993822	0.4471285
			$r^2 \leq 0.8$	6	263,285	Beta [SD change/SD decrease in sclerostin levels]	gIVW	-0.01860144	0.03810697	0.62545248
Step-wise pruning	LDL cholesterol	UK Biobank	$r^2 \leq 0.001$	1	262,794	Beta [SD change/SD decrease in sclerostin levels]	Wald ratio	0.05613282	0.06873121	0.41409954

			$r^2 \leq 0.1$	1	262,794	Beta [SD change/SD decrease in sclerostin levels]	gIVW	0.05613282	0.06873121	0.41409954
			$r^2 \leq 0.5$	2	262,794	Beta [SD change/SD decrease in sclerostin levels]	gIVW	0.06152508	0.06077499	0.31137453
			$r^2 \leq 0.8$	6	262,794	Beta [SD change/SD decrease in sclerostin levels]	gIVW	-0.0089142	0.03863295	0.81751619
Step-wise pruning	HDL cholesterol	UK Biobank	$r^2 \leq 0.001$	1	241,065	Beta [SD change/SD decrease in sclerostin levels]	Wald ratio	-0.14513568	0.06545376	0.02659731
			$r^2 \leq 0.1$	1	241,065	Beta [SD change/SD decrease in sclerostin levels]	gIVW	-0.14513568	0.06545376	0.02659731
			$r^2 \leq 0.5$	2	241,065	Beta [SD change/SD decrease in sclerostin levels]	gIVW	-0.12915149	0.05785454	0.02559196
			$r^2 \leq 0.8$	6	241,065	Beta [SD change/SD decrease in sclerostin levels]	gIVW	-0.14913587	0.0863536	0.08416102
Step-wise pruning	Triglycerides	UK Biobank	$r^2 \leq 0.001$	1	263,067	Beta [SD change/SD decrease in sclerostin levels]	Wald ratio	0.2810236	0.06733437	3.00E-05
			$r^2 \leq 0.1$	1	263,067	Beta [SD change/SD decrease in sclerostin levels]	gIVW	0.2810236	0.06733437	3.00E-05
			$r^2 \leq 0.5$	2	263,067	Beta [SD change/SD decrease in sclerostin levels]	gIVW	0.26412892	0.05955772	9.21E-06
			$r^2 \leq 0.8$	6	263,067	Beta [SD change/SD decrease in sclerostin levels]	gIVW	0.20606198	0.0788878	0.00899907
Step-wise pruning	Apolipoprotein-A	UK Biobank	$r^2 \leq 0.001$	1	239,707	Beta [SD change/SD decrease in sclerostin levels]	Wald ratio	-0.08536802	0.06636566	0.19832857
			$r^2 \leq 0.1$	1	239,707	Beta [SD change/SD decrease in sclerostin levels]	gIVW	-0.08536802	0.06636566	0.19832857
			$r^2 \leq 0.5$	2	239,707	Beta [SD change/SD decrease in sclerostin levels]	gIVW	-0.07523629	0.05865662	0.19961243
			$r^2 \leq 0.8$	6	239,707	Beta [SD change/SD decrease in sclerostin levels]	gIVW	-0.12005459	0.07950615	0.13104246
Step-wise pruning	Apolipoprotein-B	UK Biobank	$r^2 \leq 0.001$	1	261,986	Beta [SD change/SD decrease in sclerostin levels]	Wald ratio	0.10292112	0.06903513	0.13600051
			$r^2 \leq 0.1$	1	261,986	Beta [SD change/SD decrease in sclerostin levels]	gIVW	0.10292112	0.06903513	0.13600051
			$r^2 \leq 0.5$	2	261,986	Beta [SD change/SD decrease in sclerostin levels]	gIVW	0.12061292	0.06103368	0.04813554
			$r^2 \leq 0.8$	6	261,986	Beta [SD change/SD decrease in sclerostin levels]	gIVW	0.04324013	0.03880648	0.26517177
Step-wise pruning	C-Reactive protein	UK Biobank	$r^2 \leq 0.001$	1	262,728	Beta [SD change/SD decrease in sclerostin levels]	Wald ratio	2.95E-05	0.06887731	0.99965865
			$r^2 \leq 0.1$	1	262,728	Beta [SD change/SD decrease in sclerostin levels]	gIVW	2.95E-05	0.06887731	0.99965865
			$r^2 \leq 0.5$	2	262,728	Beta [SD change/SD decrease in sclerostin levels]	gIVW	0.02183399	0.06090094	0.71995686
			$r^2 \leq 0.8$	6	262,728	Beta [SD change/SD decrease in sclerostin levels]	gIVW	0.07297988	0.03880145	0.05999173
Step-wise pruning	Lipoprotein (a)	UK Biobank	$r^2 \leq 0.001$	1	209,333	Beta [SD change/SD decrease in sclerostin levels]	Wald ratio	-0.01551003	0.07731104	0.84099688

			$r^2 \leq 0.1$	1	209,333	Beta [SD change/SD decrease in sclerostin levels]	gIVW	-0.01551003	0.07731104	0.84099688
			$r^2 \leq 0.5$	2	209,333	Beta [SD change/SD decrease in sclerostin levels]	gIVW	-0.03528168	0.06837289	0.60584144
			$r^2 \leq 0.8$	6	209,333	Beta [SD change/SD decrease in sclerostin levels]	gIVW	0.02018616	0.05290314	0.70278158
Step-wise pruning	Glucose	UK Biobank	$r^2 \leq 0.001$	1	240,909	Beta [SD change/SD decrease in sclerostin levels]	Wald ratio	0.07511635	0.07157228	0.29394009
			$r^2 \leq 0.1$	1	240,909	Beta [SD change/SD decrease in sclerostin levels]	gIVW	0.07511635	0.07157228	0.29394009
			$r^2 \leq 0.5$	2	240,909	Beta [SD change/SD decrease in sclerostin levels]	gIVW	0.09284299	0.06323605	0.14205071
			$r^2 \leq 0.8$	6	240,909	Beta [SD change/SD decrease in sclerostin levels]	gIVW	0.05817519	0.04019481	0.14780427
Step-wise pruning	HbA1c	UK Biobank	$r^2 \leq 0.001$	1	263,079	Beta [SD change/SD decrease in sclerostin levels]	Wald ratio	-0.02147077	0.06735942	0.74991662
			$r^2 \leq 0.1$	1	263,079	Beta [SD change/SD decrease in sclerostin levels]	gIVW	-0.02147077	0.06735942	0.74991662
			$r^2 \leq 0.5$	2	263,079	Beta [SD change/SD decrease in sclerostin levels]	gIVW	0.00511291	0.05955887	0.93158865
			$r^2 \leq 0.8$	6	263,079	Beta [SD change/SD decrease in sclerostin levels]	gIVW	-0.00676307	0.03773946	0.85777749
Step-wise pruning	Coronary artery disease	UK Biobank (categorical)	$r^2 \leq 0.001$	1	276,172	OR / SD decrease in sclerostin levels	Wald ratio	1.90037182	0.29201352	0.0278994
			$r^2 \leq 0.1$	1	276,172	OR / SD decrease in sclerostin levels	gIVW	1.90037182	0.29201352	0.0278994
			$r^2 \leq 0.5$	2	276,172	OR / SD decrease in sclerostin levels	gIVW	1.84897728	0.2572956	0.01690262
			$r^2 \leq 0.8$	6	276,172	OR / SD decrease in sclerostin levels	gIVW	1.18382334	0.2135353	0.42937344
Step-wise pruning	Myocardial infarction	UK Biobank (categorical)	$r^2 \leq 0.001$	1	276,065	OR / SD decrease in sclerostin levels	Wald ratio	1.94938433	0.35089193	0.05712752
			$r^2 \leq 0.1$	1	276,065	OR / SD decrease in sclerostin levels	gIVW	1.94938433	0.35089193	0.05712752
			$r^2 \leq 0.5$	2	276,065	OR / SD decrease in sclerostin levels	gIVW	1.37578563	0.31152161	0.30579464
			$r^2 \leq 0.8$	6	276,065	OR / SD decrease in sclerostin levels	gIVW	1.05222933	0.27652222	0.85392552
Step-wise pruning	Ischaemic stroke	UK Biobank (categorical)	$r^2 \leq 0.001$	1	276,172	OR / SD decrease in sclerostin levels	Wald ratio	1.06633701	0.57773916	0.91147858
			$r^2 \leq 0.1$	1	276,172	OR / SD decrease in sclerostin levels	gIVW	1.06633701	0.57773916	0.91147858
			$r^2 \leq 0.5$	2	276,172	OR / SD decrease in sclerostin levels	gIVW	1.28494794	0.50747985	0.62127395
			$r^2 \leq 0.8$	6	276,172	OR / SD decrease in sclerostin levels	gIVW	0.76487017	0.4980223	0.59042017
Step-wise pruning	Hypertension	UK Biobank (categorical)	$r^2 \leq 0.001$	1	271,359	OR / SD decrease in sclerostin levels	Wald ratio	1.10875316	0.14752171	0.48405046
			$r^2 \leq 0.1$	1	271,359	OR / SD decrease in sclerostin levels	gIVW	1.10875316	0.14752171	0.48405046
			$r^2 \leq 0.5$	2	271,359	OR / SD decrease in sclerostin levels	gIVW	1.18101945	0.13033265	0.20175612
			$r^2 \leq 0.8$	6	271,359	OR / SD decrease in sclerostin levels	gIVW	1.05420031	0.08289579	0.52429883
			$r^2 \leq 0.001$	1	266,268	OR / SD decrease in sclerostin levels	Wald ratio	1.53819406	0.25258953	0.08823586

Step-wise pruning	Type 2 diabetes	UK Biobank (categorical)	$r^2 \leq 0.1$	1	266,268	OR / SD decrease in sclerostin levels	gIVW	1.53819406	0.25258953	0.08823586
			$r^2 \leq 0.5$	2	266,268	OR / SD decrease in sclerostin levels	gIVW	1.62002558	0.22238731	0.03005406
			$r^2 \leq 0.8$	6	266,268	OR / SD decrease in sclerostin levels	gIVW	1.45213146	0.14176496	0.00850478
Step-wise pruning	Coronary artery disease	UK Biobank (survival)	$r^2 \leq 0.001$	1	276,172	HR / SD decrease in sclerostin levels	Wald ratio	1.81289761	0.27670977	0.03155508
			$r^2 \leq 0.1$	1	276,172	HR / SD decrease in sclerostin levels	gIVW	1.81289761	0.27670977	0.03155508
			$r^2 \leq 0.5$	2	276,172	HR / SD decrease in sclerostin levels	gIVW	1.7700273	0.2437155	0.01913579
			$r^2 \leq 0.8$	6	276,172	HR / SD decrease in sclerostin levels	gIVW	1.16853246	0.20485376	0.44708013
Step-wise pruning	Myocardial infarction	UK Biobank (survival)	$r^2 \leq 0.001$	1	276,172	HR / SD decrease in sclerostin levels	Wald ratio	1.91498016	0.33999829	0.05601572
			$r^2 \leq 0.1$	1	276,172	HR / SD decrease in sclerostin levels	gIVW	1.91498016	0.33999829	0.05601572
			$r^2 \leq 0.5$	2	276,172	HR / SD decrease in sclerostin levels	gIVW	1.36960736	0.30190278	0.29750167
			$r^2 \leq 0.8$	6	276,172	HR / SD decrease in sclerostin levels	gIVW	1.04821662	0.27170394	0.86240413
Step-wise pruning	Ischaemic stroke	UK Biobank (survival)	$r^2 \leq 0.001$	1	276,172	HR / SD decrease in sclerostin levels	Wald ratio	1.05794812	0.57161586	0.92149758
			$r^2 \leq 0.1$	1	276,172	HR / SD decrease in sclerostin levels	gIVW	1.05794812	0.57161586	0.92149758
			$r^2 \leq 0.5$	2	276,172	HR / SD decrease in sclerostin levels	gIVW	1.285523	0.50198067	0.6168292
			$r^2 \leq 0.8$	6	276,172	HR / SD decrease in sclerostin levels	gIVW	0.77599628	0.49390784	0.6076216
Step-wise pruning	Hypertension	UK Biobank (survival)	$r^2 \leq 0.001$	1	276,172	HR / SD decrease in sclerostin levels	Wald ratio	1.07213831	0.11585293	0.5476821
			$r^2 \leq 0.1$	1	276,172	HR / SD decrease in sclerostin levels	gIVW	1.07213831	0.11585293	0.5476821
			$r^2 \leq 0.5$	2	276,172	HR / SD decrease in sclerostin levels	gIVW	1.12022218	0.10221314	0.26670209
			$r^2 \leq 0.8$	6	276,172	HR / SD decrease in sclerostin levels	gIVW	1.03596301	0.06504502	0.58700288
Step-wise pruning	Type 2 diabetes	UK Biobank (survival)	$r^2 \leq 0.001$	1	276,172	HR / SD decrease in sclerostin levels	Wald ratio	1.56900258	0.32568088	0.16664279
			$r^2 \leq 0.1$	1	276,172	HR / SD decrease in sclerostin levels	gIVW	1.56900258	0.32568088	0.16664279
			$r^2 \leq 0.5$	2	276,172	HR / SD decrease in sclerostin levels	gIVW	1.66881184	0.28629863	0.07365778
			$r^2 \leq 0.8$	6	276,172	HR / SD decrease in sclerostin levels	gIVW	1.65459767	0.18232995	0.00574846
Random-effects method	Heel bone mineral density	GWAS	$r^2 \leq 0.3$	1	426,824	Beta [SD change/SD decrease in sclerostin levels]	Wald ratio	1.12486981	0.0552528	3.90E-92
Random-effects method	Hip fracture	GWAS	$r^2 \leq 0.3$	1	735,354	OR [SD change/SD decrease in sclerostin levels]	Wald ratio	0.12116527	0.4270796	7.74E-07
Random-effects method	LDL cholesterol	GWAS	$r^2 \leq 0.3$	1	1,228,508	Beta [mg/dL change/SD decrease in sclerostin levels]	Wald ratio	0.01730604	0.04112336	0.67387754
Random-effects method	HDL cholesterol	GWAS	$r^2 \leq 0.3$	1	1,244,439	Beta [mg/dL change/SD decrease in sclerostin levels]	Wald ratio	-0.30898245	0.04076048	3.44E-14

Random-effects method	Fasting glucose	GWAS	$r^2 \leq 0.3$	1	178,455	Beta [mmol/L change/SD decrease in sclerostin levels]	Wald ratio	-0.01102141	0.05235169	0.83325692
Random-effects method	HbA1c	GWAS	$r^2 \leq 0.3$	1	132,400	Beta [% change/SD decrease in sclerostin levels]	Wald ratio	0.01928747	0.03857493	0.61707508
Random-effects method	Coronary artery disease	GWAS	$r^2 \leq 0.3$	1	1,165,690	OR [SD change/SD decrease in sclerostin levels]	Wald ratio	1.50850935	0.1456142	0.00475225
Random-effects method	Myocardial infarction	GWAS	$r^2 \leq 0.3$	1	~639,000	OR [SD change/SD decrease in sclerostin levels]	Wald ratio	1.4619486	0.22020775	0.08459966
Random-effects method	Ischaemic stroke	GWAS	$r^2 \leq 0.3$	1	1,847,683	OR [SD change/SD decrease in sclerostin levels]	Wald ratio	1.11038121	0.20114072	0.60268172
Random-effects method	Hypertension	GWAS	$r^2 \leq 0.3$	1	462,933	OR [SD change/SD decrease in sclerostin levels]	Wald ratio	1.03209637	0.02681732	0.23877818
Random-effects method	Type 2 diabetes	GWAS	$r^2 \leq 0.3$	1	933,970	OR [SD change/SD decrease in sclerostin levels]	Wald ratio	1.81831717	0.18736395	0.00141695
Random-effects method	Cholesterol	UK Biobank	$r^2 \leq 0.3$	1	263,285	Beta [SD change/SD decrease in sclerostin levels]	Wald ratio	0.03712163	0.08183082	0.65008887
Random-effects method	LDL cholesterol	UK Biobank	$r^2 \leq 0.3$	1	262,794	Beta [SD change/SD decrease in sclerostin levels]	Wald ratio	0.07041799	0.08297635	0.39607537
Random-effects method	HDL cholesterol	UK Biobank	$r^2 \leq 0.3$	1	241,065	Beta [SD change/SD decrease in sclerostin levels]	Wald ratio	-0.30712607	0.07900578	0.00010133
Random-effects method	Triglycerides	UK Biobank	$r^2 \leq 0.3$	1	263,067	Beta [SD change/SD decrease in sclerostin levels]	Wald ratio	0.45840662	0.08130907	1.72E-08
Random-effects method	Apolipoprotein-A	UK Biobank	$r^2 \leq 0.3$	1	239,707	Beta [SD change/SD decrease in sclerostin levels]	Wald ratio	-0.22392764	0.08010977	0.0051858
Random-effects method	Apolipoprotein-B	UK Biobank	$r^2 \leq 0.3$	1	261,986	Beta [SD change/SD decrease in sclerostin levels]	Wald ratio	0.15026865	0.08333518	0.07135937
Random-effects method	C-Reactive protein	UK Biobank	$r^2 \leq 0.3$	1	262,728	Beta [SD change/SD decrease in sclerostin levels]	Wald ratio	0.04158791	0.0831949	0.61715578
Random-effects method	Lipoprotein (a)	UK Biobank	$r^2 \leq 0.3$	1	209,333	Beta [SD change/SD decrease in sclerostin levels]	Wald ratio	-0.03791987	0.09336314	0.684629

Random-effects method	Glucose	UK Biobank	$r^2 \leq 0.3$	1	240,909	Beta [SD change/SD decrease in sclerostin levels]	Wald ratio	0.065245	0.086256	0.44940274
Random-effects method	HbA1c	UK Biobank	$r^2 \leq 0.3$	1	263,079	Beta [SD change/SD decrease in sclerostin levels]	Wald ratio	0.00577451	0.08129073	0.94336968
Random-effects method	Coronary artery disease	UK Biobank (categorical)	$r^2 \leq 0.3$	1	276,172	OR [SD change/SD decrease in sclerostin levels]	Wald ratio	2.72020211	0.35153142	0.00441743
Random-effects method	Myocardial infarction	UK Biobank (categorical)	$r^2 \leq 0.3$	1	276,065	OR [SD change/SD decrease in sclerostin levels]	Wald ratio	2.66706624	0.42266155	0.02028919
Random-effects method	Ischaemic stroke	UK Biobank (categorical)	$r^2 \leq 0.3$	1	276,172	OR [SD change/SD decrease in sclerostin levels]	Wald ratio	2.93811502	0.69288847	0.11983383
Random-effects method	Hypertension	UK Biobank (categorical)	$r^2 \leq 0.3$	1	271,359	OR [SD change/SD decrease in sclerostin levels]	Wald ratio	1.2325754	0.1780945	0.24034363
Random-effects method	Type 2 diabetes	UK Biobank (categorical)	$r^2 \leq 0.3$	1	266,268	OR [SD change/SD decrease in sclerostin levels]	Wald ratio	1.50858669	0.30488516	0.17746009
Random-effects method	Coronary artery disease	UK Biobank (survival)	$r^2 \leq 0.3$	1	276,172	HR [SD change/SD decrease in sclerostin levels]	Wald ratio	2.58383609	0.33310728	0.00437525
Random-effects method	Myocardial infarction	UK Biobank (survival)	$r^2 \leq 0.3$	1	276,172	HR [SD change/SD decrease in sclerostin levels]	Wald ratio	2.61652135	0.40944941	0.01881785
Random-effects method	Ischaemic stroke	UK Biobank (survival)	$r^2 \leq 0.3$	1	276,172	HR [SD change/SD decrease in sclerostin levels]	Wald ratio	2.89519075	0.68559593	0.12100948
Random-effects method	Hypertension	UK Biobank (survival)	$r^2 \leq 0.3$	1	276,172	HR [SD change/SD decrease in sclerostin levels]	Wald ratio	1.18803669	0.13987401	0.21800965
Random-effects method	Type 2 diabetes	UK Biobank (survival)	$r^2 \leq 0.3$	1	276,172	HR [SD change/SD decrease in sclerostin levels]	Wald ratio	1.42239061	0.39325945	0.37028194
Principal components analysis	Heel bone mineral density	GWAS	$r^2 \leq 0.3$	76	426,824	Beta [SD change/SD decrease in sclerostin levels]	IVW	0.94154294	0.03766465	2.07E-121
Principal components analysis	Hip fracture	GWAS	$r^2 \leq 0.3$	76	735,354	OR [SD change/SD decrease in sclerostin levels]	IVW	0.19890407	0.29719333	9.40E-08

Principal components analysis	LDL cholesterol	GWAS	$r^2 \leq 0.3$	76	1,228,508	Beta [mg/dL change/SD decrease in sclerostin levels]	IVW	0.00208898	0.02869987	0.94706043
Principal components analysis	HDL cholesterol	GWAS	$r^2 \leq 0.3$	76	1,244,439	Beta [mg/dL change/SD decrease in sclerostin levels]	IVW	-0.11481814	0.02847307	6.40E-05
Principal components analysis	Fasting glucose	GWAS	$r^2 \leq 0.3$	76	178,455	Beta [mmol/L change/SD decrease in sclerostin levels]	IVW	0.03867948	0.03779884	0.31057976
Principal components analysis	HbA1c	GWAS	$r^2 \leq 0.3$	76	132,400	Beta [% change/SD decrease in sclerostin levels]	IVW	0.01175036	0.02868469	0.6952268
Principal components analysis	Coronary artery disease	GWAS	$r^2 \leq 0.3$	76	1,165,690	OR [SD change/SD decrease in sclerostin levels]	IVW	1.20602835	0.10233798	0.06677434
Principal components analysis	Myocardial infarction	GWAS	$r^2 \leq 0.3$	76	~639,000	OR [SD change/SD decrease in sclerostin levels]	IVW	1.30150483	0.15644801	0.09181376
Principal components analysis	Ischaemic stroke	GWAS	$r^2 \leq 0.3$	76	1,847,683	OR [SD change/SD decrease in sclerostin levels]	IVW	1.15602434	0.14550135	0.32383292
Principal components analysis	Hypertension	GWAS	$r^2 \leq 0.3$	76	462,933	OR [SD change/SD decrease in sclerostin levels]	IVW	1.02140871	0.01837844	0.25181914
Principal components analysis	Type 2 diabetes	GWAS	$r^2 \leq 0.3$	76	933,970	OR [SD change/SD decrease in sclerostin levels]	IVW	1.42863957	0.13029785	0.00621373
Survival analysis since UK Biobank enrolment	Coronary artery disease	UK Biobank (survival)	$r^2 \leq 0.3$	2	276,172	HR [SD change/SD decrease in sclerostin levels]	gIVW	1.82073661	0.29815486	0.04444898
Survival analysis since UK Biobank enrolment	Myocardial infarction	UK Biobank (survival)	$r^2 \leq 0.3$	2	276,172	HR [SD change/SD decrease in sclerostin levels]	gIVW	1.32316048	0.46505493	0.5470881
Survival analysis since UK Biobank enrolment	Ischaemic stroke	UK Biobank (survival)	$r^2 \leq 0.3$	2	276,172	HR [SD change/SD decrease in sclerostin levels]	gIVW	1.05959671	0.5518593	0.91645755
Survival analysis since UK Biobank enrolment	Hypertension	UK Biobank (survival)	$r^2 \leq 0.3$	2	276,172	HR [SD change/SD decrease in sclerostin levels]	gIVW	1.2575155	0.20022176	0.25244917
Survival analysis since UK Biobank enrolment	Type 2 diabetes	UK Biobank (survival)	$r^2 \leq 0.3$	2	276,172	HR [SD change/SD decrease in sclerostin levels]	gIVW	1.95012545	0.37133237	0.07207546

Supplementary Table 10. Phenotyping codes used for UK Biobank outcomes.

Continuous outcomes						
Source data: UK Biobank						
**Field**	**Code**	**Description**	**Extra information**	**As continuous outcome**		
3148.0.0		Heel bone mineral density (BMD)		Standarized to SD Value		
30690.0.0		Cholesterol		Standarized to SD Value		
30780.0.0		LDL direct		Standarized to SD Value		
30760.0.0		HDL cholesterol		Standarized to SD Valud		
30870.0.0		Triglycerides		Standarized to SD Value		
30630.0.0		Apolipoprotein A		Standarized to SD Value		
30640.0.0		Apolipoprotein B		Standarized to SD Value		
30710.0.0		C-Reactive protein		Standarized to SD Value		
30790.0.0		Lipoprotein A		Standarized to SD Value		
30750.0.0		Glycated haemoglobin (HbA1c)		Standarized to SD Value		
30740.0.0		Glucose		Standarized to SD Value		
**Fracture risk outcome**						
Source data: UK Biobank						
**Field**	**Code**	**Description**	**Extra information**	**As binary utcome**	**As survival outcome (since birth date)**	**As survival outcome (since enrolment date)**
3005		Fracture resulting from simple fall (from the last five years)		Patient included	Patient included, date of the event is the UK Biobank recruitment date (Field 53)	Patient excluded
20002	1632	fracture shoulder / scapula		Patient included	Patient included, years at the event is Field 20009 (Interpolated year/age of the event)	Patient excluded
20002	1633	fracture upper arm / humerus / elbow		Patient included	Patient included, years at the event is Field 20009 (Interpolated year/age of the event)	Patient excluded
20002	1634	fracture forearm / wrist		Patient included	Patient included, years at the event is Field 20009 (Interpolated year/age of the event)	Patient excluded
20002	1635	fracture radius		Patient included	Patient included, years at the event is Field 20009 (Interpolated year/age of the event)	Patient excluded
20002	1637	fracture wrist / colles fracture		Patient included	Patient included, years at the event is Field 20009 (Interpolated year/age of the event)	Patient excluded
20002	1644	fracture rib		Patient included	Patient included, years at the event is Field 20009 (Interpolated year/age of the event)	Patient excluded
20002	1646	fracture vertebra / crush fracture / vertebral collapse		Patient included	Patient included, years at the event is Field 20009 (Interpolated year/age of the event)	Patient excluded
20002	1647	fracture pelvis		Patient included	Patient included, years at the event is Field 20009 (Interpolated year/age of the event)	Patient excluded
20002	1648	fracture neck of femur / hip		Patient included	Patient included, years at the event is Field 20009 (Interpolated year/age of the event)	Patient excluded
Source data: Linkage to HES						
**ICD-10**	**Code**	**Description**	Extra information	As binary utcome	As survival outcome (since birth date)	As survival outcome (since enrolment date)
S720		Fracture of neck of femur		Patient included	Patient included, date of the event is the minimum between "epistart" and "admidate"	Patient included if the first event was after the UK Biobank recruitment date (Field 53), date of the event is the minimum between "epistart" and "admidate"
S721		Pertrochanteric fracture		Patient included	Patient included, date of the event is the minimum between "epistart" and "admidate"	Patient included if the first event was after the UK Biobank recruitment date (Field 53), date of the event is the minimum between "epistart" and "admidate"
S729		Fracture of femur, part unspecified		Patient included	Patient included, date of the event is the minimum between "epistart" and "admidate"	Patient included if the first event was after the UK Biobank recruitment date (Field 53), date of the event is the minimum between "epistart" and "admidate"
S722		Subtrochanteric fracture		Patient included	Patient included, date of the event is the minimum between "epistart" and "admidate"	Patient included if the first event was after the UK Biobank recruitment date (Field 53), date of the event is the minimum between "epistart" and "admidate"
S327		Multiple fractures of lumbar spine and pelvis		Patient included	Patient included, date of the event is the minimum between "epistart" and "admidate"	Patient included if the first event was after the UK Biobank recruitment date (Field 53), date of the event is the minimum between "epistart" and "admidate"
S321		Fracture of sacrum		Patient included	Patient included, date of the event is the minimum between "epistart" and "admidate"	Patient included if the first event was after the UK Biobank recruitment date (Field 53), date of the event is the minimum between "epistart" and "admidate"

S322		Fracture of coccyx		Patient included	Patient included, date of the event is the minimum between "epistart" and "admidate"	Patient included if the first event was after the UK Biobank recruitment date (Field 53), date of the event is the minimum between "epistart" and "admidate"
S323		Fracture of ilium		Patient included	Patient included, date of the event is the minimum between "epistart" and "admidate"	Patient included if the first event was after the UK Biobank recruitment date (Field 53), date of the event is the minimum between "epistart" and "admidate"
S324		Fracture of acetabulum		Patient included	Patient included, date of the event is the minimum between "epistart" and "admidate"	Patient included if the first event was after the UK Biobank recruitment date (Field 53), date of the event is the minimum between "epistart" and "admidate"
S325		Fracture of pubis		Patient included	Patient included, date of the event is the minimum between "epistart" and "admidate"	Patient included if the first event was after the UK Biobank recruitment date (Field 53), date of the event is the minimum between "epistart" and "admidate"
S328		Fracture of other and unspecified parts of lumbar spine and pelvis		Patient included	Patient included, date of the event is the minimum between "epistart" and "admidate"	Patient included if the first event was after the UK Biobank recruitment date (Field 53), date of the event is the minimum between "epistart" and "admidate"
S422		Fracture of upper end of humerus		Patient included	Patient included, date of the event is the minimum between "epistart" and "admidate"	Patient included if the first event was after the UK Biobank recruitment date (Field 53), date of the event is the minimum between "epistart" and "admidate"
S423		Fracture of shaft of humerus		Patient included	Patient included, date of the event is the minimum between "epistart" and "admidate"	Patient included if the first event was after the UK Biobank recruitment date (Field 53), date of the event is the minimum between "epistart" and "admidate"
S428		Fracture of other parts of shoulder and upper arm		Patient included	Patient included, date of the event is the minimum between "epistart" and "admidate"	Patient included if the first event was after the UK Biobank recruitment date (Field 53), date of the event is the minimum between "epistart" and "admidate"
S220		Fracture of thoracic vertebra		Patient included	Patient included, date of the event is the minimum between "epistart" and "admidate"	Patient included if the first event was after the UK Biobank recruitment date (Field 53), date of the event is the minimum between "epistart" and "admidate"
S221		Multiple fractures of thoracic spine		Patient included	Patient included, date of the event is the minimum between "epistart" and "admidate"	Patient included if the first event was after the UK Biobank recruitment date (Field 53), date of the event is the minimum between "epistart" and "admidate"
S320		Fracture of lumbar vertebra		Patient included	Patient included, date of the event is the minimum between "epistart" and "admidate"	Patient included if the first event was after the UK Biobank recruitment date (Field 53), date of the event is the minimum between "epistart" and "admidate"
S223		Fracture of rib		Patient included	Patient included, date of the event is the minimum between "epistart" and "admidate"	Patient included if the first event was after the UK Biobank recruitment date (Field 53), date of the event is the minimum between "epistart" and "admidate"
S224		Multiple fractures of ribs		Patient included	Patient included, date of the event is the minimum between "epistart" and "admidate"	Patient included if the first event was after the UK Biobank recruitment date (Field 53), date of the event is the minimum between "epistart" and "admidate"
S522		Fracture of shaft of ulna		Patient included	Patient included, date of the event is the minimum between "epistart" and "admidate"	Patient included if the first event was after the UK Biobank recruitment date (Field 53), date of the event is the minimum between "epistart" and "admidate"
S523		Fracture of shaft of radius		Patient included	Patient included, date of the event is the minimum between "epistart" and "admidate"	Patient included if the first event was after the UK Biobank recruitment date (Field 53), date of the event is the minimum between "epistart" and "admidate"
S524		Fracture of shafts of both ulna and radius		Patient included	Patient included, date of the event is the minimum between "epistart" and "admidate"	Patient included if the first event was after the UK Biobank recruitment date (Field 53), date of the event is the minimum between "epistart" and "admidate"
S525		Fracture of lower end of radius		Patient included	Patient included, date of the event is the minimum between "epistart" and "admidate"	Patient included if the first event was after the UK Biobank recruitment date (Field 53), date of the event is the minimum between "epistart" and "admidate"
S526		Fracture of lower end of both ulna and radius		Patient included	Patient included, date of the event is the minimum between "epistart" and "admidate"	Patient included if the first event was after the UK Biobank recruitment date (Field 53), date of the event is the minimum between "epistart" and "admidate"
S527		Multiple fractures of forearm		Patient included	Patient included, date of the event is the minimum between "epistart" and "admidate"	Patient included if the first event was after the UK Biobank recruitment date (Field 53), date of the event is the minimum between "epistart" and "admidate"
S528		Fracture of other parts of forearm		Patient included	Patient included, date of the event is the minimum between "epistart" and "admidate"	Patient included if the first event was after the UK Biobank recruitment date (Field 53), date of the event is the minimum between "epistart" and "admidate"

S529		Fracture of forearm, part unspecified		Patient included	Patient included, date of the event is the minimum between "epistart" and "admidate"	Patient included if the first event was after the UK Biobank recruitment date (Field 53), date of the event is the minimum between "epistart" and "admidate"
Source data: Linkage to GP						
**Read2 code**	**Code**	**Description**	**Extra information**	**As binary utcome**	**As survival outcome (since birth date)**	**As survival outcome (since enrolment date)**
7K1D0		Primary open reduction and internal fixation of proximal femoral fracture with screw/nail and plate device		Patient included	Patient included, date of the event is "event_dt"	Patient included if the first event was after the UK Biobank recruitment date (Field 53), date of the event is "event_dt"
7K1DE		Primary open reduction of fracture of neck of femur and open fixation using proximal femoral nail antirotation		Patient included	Patient included, date of the event is "event_dt"	Patient included if the first event was after the UK Biobank recruitment date (Field 53), date of the event is "event_dt"
7K1E6		Primary open reduction of fracture of elbow and fixation with Hook fixation plate		Patient included	Patient included, date of the event is "event_dt"	Patient included if the first event was after the UK Biobank recruitment date (Field 53), date of the event is "event_dt"
7K1J0		Closed reduction and internal fixation of proximal femoral fracture with screw/nail device alone		Patient included	Patient included, date of the event is "event_dt"	Patient included if the first event was after the UK Biobank recruitment date (Field 53), date of the event is "event_dt"
7K1Jd		Closed reduction of intracapsular fracture of neck of femur and internal fixation using a dynamic hip screw		Patient included	Patient included, date of the event is "event_dt"	Patient included if the first event was after the UK Biobank recruitment date (Field 53), date of the event is "event_dt"
7K1L4		Closed reduction of fracture of hip		Patient included	Patient included, date of the event is "event_dt"	Patient included if the first event was after the UK Biobank recruitment date (Field 53), date of the event is "event_dt"
7K1L5		Closed reduction of fracture of femur		Patient included	Patient included, date of the event is "event_dt"	Patient included if the first event was after the UK Biobank recruitment date (Field 53), date of the event is "event_dt"
7K1LE		Closed reduction of fracture of elbow		Patient included	Patient included, date of the event is "event_dt"	Patient included if the first event was after the UK Biobank recruitment date (Field 53), date of the event is "event_dt"
7K1LF		Closed reduction of fracture of humerus		Patient included	Patient included, date of the event is "event_dt"	Patient included if the first event was after the UK Biobank recruitment date (Field 53), date of the event is "event_dt"
7K1LG		Closed reduction of fracture of shoulder		Patient included	Patient included, date of the event is "event_dt"	Patient included if the first event was after the UK Biobank recruitment date (Field 53), date of the event is "event_dt"
7K1LL		Closed reduction of fracture of radius and or ulna		Patient included	Patient included, date of the event is "event_dt"	Patient included if the first event was after the UK Biobank recruitment date (Field 53), date of the event is "event_dt"
7K1LM		Closed reduction of fracture of wrist		Patient included	Patient included, date of the event is "event_dt"	Patient included if the first event was after the UK Biobank recruitment date (Field 53), date of the event is "event_dt"
N1y1.		Fatigue fracture of vertebra		Patient included	Patient included, date of the event is "event_dt"	Patient included if the first event was after the UK Biobank recruitment date (Field 53), date of the event is "event_dt"
N3310		Pathological fracture of thoracic vertebra		Patient included	Patient included, date of the event is "event_dt"	Patient included if the first event was after the UK Biobank recruitment date (Field 53), date of the event is "event_dt"
N3311		Pathological fracture of lumbar vertebra		Patient included	Patient included, date of the event is "event_dt"	Patient included if the first event was after the UK Biobank recruitment date (Field 53), date of the event is "event_dt"
N3318		Osteoporosis + pathological fracture lumbar vertebrae		Patient included	Patient included, date of the event is "event_dt"	Patient included if the first event was after the UK Biobank recruitment date (Field 53), date of the event is "event_dt"
N3319		Osteoporosis + pathological fracture thoracic vertebrae		Patient included	Patient included, date of the event is "event_dt"	Patient included if the first event was after the UK Biobank recruitment date (Field 53), date of the event is "event_dt"
S102.		Closed fracture thoracic vertebra		Patient included	Patient included, date of the event is "event_dt"	Patient included if the first event was after the UK Biobank recruitment date (Field 53), date of the event is "event_dt"
S1020		Closed fracture thoracic vertebra, burst		Patient included	Patient included, date of the event is "event_dt"	Patient included if the first event was after the UK Biobank recruitment date (Field 53), date of the event is "event_dt"
S1021		Closed fracture thoracic vertebra, wedge		Patient included	Patient included, date of the event is "event_dt"	Patient included if the first event was after the UK Biobank recruitment date (Field 53), date of the event is "event_dt"
S1022		Closed fracture thoracic vertebra, spondylolysis		Patient included	Patient included, date of the event is "event_dt"	Patient included if the first event was after the UK Biobank recruitment date (Field 53), date of the event is "event_dt"
S102y		Other specified closed fracture thoracic vertebra		Patient included	Patient included, date of the event is "event_dt"	Patient included if the first event was after the UK Biobank recruitment date (Field 53), date of the event is "event_dt"
S102z		Closed fracture thoracic vertebra not otherwise specified		Patient included	Patient included, date of the event is "event_dt"	Patient included if the first event was after the UK Biobank recruitment date (Field 53), date of the event is "event_dt"
S104.		Closed fracture lumbar vertebra		Patient included	Patient included, date of the event is "event_dt"	Patient included if the first event was after the UK Biobank recruitment date (Field 53), date of the event is "event_dt"
S1040		Closed fracture lumbar vertebra, burst		Patient included	Patient included, date of the event is "event_dt"	Patient included if the first event was after the UK Biobank recruitment date (Field 53), date of the event is "event_dt"

S1041		Closed fracture lumbar vertebra, wedge		Patient included	Patient included, date of the event is "event_dt"	Patient included if the first event was after the UK Biobank recruitment date (Field 53), date of the event is "event_dt"
S1042		Closed fracture lumbar vertebra, spondylolysis		Patient included	Patient included, date of the event is "event_dt"	Patient included if the first event was after the UK Biobank recruitment date (Field 53), date of the event is "event_dt"
S106.		Closed fracture sacrum		Patient included	Patient included, date of the event is "event_dt"	Patient included if the first event was after the UK Biobank recruitment date (Field 53), date of the event is "event_dt"
S1060		Closed compression fracture sacrum		Patient included	Patient included, date of the event is "event_dt"	Patient included if the first event was after the UK Biobank recruitment date (Field 53), date of the event is "event_dt"
S1061		Closed vertical fracture of sacrum		Patient included	Patient included, date of the event is "event_dt"	Patient included if the first event was after the UK Biobank recruitment date (Field 53), date of the event is "event_dt"
S108.		Closed fracture pelvis, coccyx		Patient included	Patient included, date of the event is "event_dt"	Patient included if the first event was after the UK Biobank recruitment date (Field 53), date of the event is "event_dt"
S10B6		Multiple fractures of lumbar spine and pelvis		Patient included	Patient included, date of the event is "event_dt"	Patient included if the first event was after the UK Biobank recruitment date (Field 53), date of the event is "event_dt"
S112.		Closed fracture of thoracic spine with spinal cord lesion		Patient included	Patient included, date of the event is "event_dt"	Patient included if the first event was after the UK Biobank recruitment date (Field 53), date of the event is "event_dt"
S112z		Closed fracture of thoracic spine with spinal cord lesion NOS		Patient included	Patient included, date of the event is "event_dt"	Patient included if the first event was after the UK Biobank recruitment date (Field 53), date of the event is "event_dt"
S114.		Closed fracture of lumbar spine with spinal cord lesion		Patient included	Patient included, date of the event is "event_dt"	Patient included if the first event was after the UK Biobank recruitment date (Field 53), date of the event is "event_dt"
S116.		Closed fracture of sacrum with spinal cord lesion		Patient included	Patient included, date of the event is "event_dt"	Patient included if the first event was after the UK Biobank recruitment date (Field 53), date of the event is "event_dt"
S1160		Closed fracture of sacrum with unspecified spinal cord lesion		Patient included	Patient included, date of the event is "event_dt"	Patient included if the first event was after the UK Biobank recruitment date (Field 53), date of the event is "event_dt"
S1161		Closed fracture of sacrum with complete cauda equina lesion		Patient included	Patient included, date of the event is "event_dt"	Patient included if the first event was after the UK Biobank recruitment date (Field 53), date of the event is "event_dt"
S1162		Closed fracture of sacrum with other cauda equina injury		Patient included	Patient included, date of the event is "event_dt"	Patient included if the first event was after the UK Biobank recruitment date (Field 53), date of the event is "event_dt"
S1163		Closed fracture of sacrum with other spinal cord injury		Patient included	Patient included, date of the event is "event_dt"	Patient included if the first event was after the UK Biobank recruitment date (Field 53), date of the event is "event_dt"
S116z		Closed fracture of sacrum with spinal cord lesion NOS		Patient included	Patient included, date of the event is "event_dt"	Patient included if the first event was after the UK Biobank recruitment date (Field 53), date of the event is "event_dt"
S118.		Closed fracture of coccyx with spinal cord lesion		Patient included	Patient included, date of the event is "event_dt"	Patient included if the first event was after the UK Biobank recruitment date (Field 53), date of the event is "event_dt"
S1180		Closed fracture of coccyx with unspecified spinal cord lesion		Patient included	Patient included, date of the event is "event_dt"	Patient included if the first event was after the UK Biobank recruitment date (Field 53), date of the event is "event_dt"
S1181		Closed fracture of coccyx with complete cauda equina lesion		Patient included	Patient included, date of the event is "event_dt"	Patient included if the first event was after the UK Biobank recruitment date (Field 53), date of the event is "event_dt"
S1182		Closed fracture of coccyx with other cauda equina injury		Patient included	Patient included, date of the event is "event_dt"	Patient included if the first event was after the UK Biobank recruitment date (Field 53), date of the event is "event_dt"
S1183		Closed fracture of coccyx with other spinal cord injury		Patient included	Patient included, date of the event is "event_dt"	Patient included if the first event was after the UK Biobank recruitment date (Field 53), date of the event is "event_dt"
S118z		Closed fracture of coccyx with spinal cord lesion NOS		Patient included	Patient included, date of the event is "event_dt"	Patient included if the first event was after the UK Biobank recruitment date (Field 53), date of the event is "event_dt"
S120.		Closed fracture rib		Patient included	Patient included, date of the event is "event_dt"	Patient included if the first event was after the UK Biobank recruitment date (Field 53), date of the event is "event_dt"
S1200		Closed fracture of rib, unspecified		Patient included	Patient included, date of the event is "event_dt"	Patient included if the first event was after the UK Biobank recruitment date (Field 53), date of the event is "event_dt"
S1201		Closed fracture of one rib		Patient included	Patient included, date of the event is "event_dt"	Patient included if the first event was after the UK Biobank recruitment date (Field 53), date of the event is "event_dt"
S1202		Closed fracture of two ribs		Patient included	Patient included, date of the event is "event_dt"	Patient included if the first event was after the UK Biobank recruitment date (Field 53), date of the event is "event_dt"
S1203		Closed fracture of three ribs		Patient included	Patient included, date of the event is "event_dt"	Patient included if the first event was after the UK Biobank recruitment date (Field 53), date of the event is "event_dt"
S1204		Closed fracture of four ribs		Patient included	Patient included, date of the event is "event_dt"	Patient included if the first event was after the UK Biobank recruitment date (Field 53), date of the event is "event_dt"
S1205		Closed fracture of five ribs		Patient included	Patient included, date of the event is "event_dt"	Patient included if the first event was after the UK Biobank recruitment date (Field 53), date of the event is "event_dt"
S1206		Closed fracture of six ribs		Patient included	Patient included, date of the event is "event_dt"	Patient included if the first event was after the UK Biobank recruitment date (Field 53), date of the event is "event_dt"

S1207		Closed fracture of seven ribs		Patient included	Patient included, date of the event is "event_dt"	Patient included if the first event was after the UK Biobank recruitment date (Field 53), date of the event is "event_dt"
S1208		Closed fracture of eight or more ribs		Patient included	Patient included, date of the event is "event_dt"	Patient included if the first event was after the UK Biobank recruitment date (Field 53), date of the event is "event_dt"
S1209		Closed fracture multiple ribs		Patient included	Patient included, date of the event is "event_dt"	Patient included if the first event was after the UK Biobank recruitment date (Field 53), date of the event is "event_dt"
S1270		Multiple fractures of ribs		Patient included	Patient included, date of the event is "event_dt"	Patient included if the first event was after the UK Biobank recruitment date (Field 53), date of the event is "event_dt"
S1271		Cough fracture of ribs		Patient included	Patient included, date of the event is "event_dt"	Patient included if the first event was after the UK Biobank recruitment date (Field 53), date of the event is "event_dt"
S130.		Closed fracture acetabulum		Patient included	Patient included, date of the event is "event_dt"	Patient included if the first event was after the UK Biobank recruitment date (Field 53), date of the event is "event_dt"
S1300		Closed fracture acetabulum, anterior lip alone		Patient included	Patient included, date of the event is "event_dt"	Patient included if the first event was after the UK Biobank recruitment date (Field 53), date of the event is "event_dt"
S1301		Closed fracture acetabulum, posterior lip alone		Patient included	Patient included, date of the event is "event_dt"	Patient included if the first event was after the UK Biobank recruitment date (Field 53), date of the event is "event_dt"
S1302		Closed fracture acetabulum, anterior column		Patient included	Patient included, date of the event is "event_dt"	Patient included if the first event was after the UK Biobank recruitment date (Field 53), date of the event is "event_dt"
S1303		Closed fracture acetabulum, posterior column		Patient included	Patient included, date of the event is "event_dt"	Patient included if the first event was after the UK Biobank recruitment date (Field 53), date of the event is "event_dt"
S1304		Closed fracture acetabulum, floor		Patient included	Patient included, date of the event is "event_dt"	Patient included if the first event was after the UK Biobank recruitment date (Field 53), date of the event is "event_dt"
S1305		Closed fracture acetabulum, double column transverse		Patient included	Patient included, date of the event is "event_dt"	Patient included if the first event was after the UK Biobank recruitment date (Field 53), date of the event is "event_dt"
S1306		Closed fracture acetabulum, double column unspecified		Patient included	Patient included, date of the event is "event_dt"	Patient included if the first event was after the UK Biobank recruitment date (Field 53), date of the event is "event_dt"
S130y		Other specified closed fracture acetabulum		Patient included	Patient included, date of the event is "event_dt"	Patient included if the first event was after the UK Biobank recruitment date (Field 53), date of the event is "event_dt"
S130z		Closed fracture acetabulum NOS		Patient included	Patient included, date of the event is "event_dt"	Patient included if the first event was after the UK Biobank recruitment date (Field 53), date of the event is "event_dt"
S132.		Closed fracture pubis		Patient included	Patient included, date of the event is "event_dt"	Patient included if the first event was after the UK Biobank recruitment date (Field 53), date of the event is "event_dt"
S1320		Closed fracture pelvis, single pubic ramus		Patient included	Patient included, date of the event is "event_dt"	Patient included if the first event was after the UK Biobank recruitment date (Field 53), date of the event is "event_dt"
S1321		Closed fracture pelvis, multiple pubic rami - stable		Patient included	Patient included, date of the event is "event_dt"	Patient included if the first event was after the UK Biobank recruitment date (Field 53), date of the event is "event_dt"
S1322		Closed fracture pelvis, multiple pubic rami - unstable		Patient included	Patient included, date of the event is "event_dt"	Patient included if the first event was after the UK Biobank recruitment date (Field 53), date of the event is "event_dt"
S132y		Other specified closed fracture pubis		Patient included	Patient included, date of the event is "event_dt"	Patient included if the first event was after the UK Biobank recruitment date (Field 53), date of the event is "event_dt"
S132z		Closed fracture pubis NOS		Patient included	Patient included, date of the event is "event_dt"	Patient included if the first event was after the UK Biobank recruitment date (Field 53), date of the event is "event_dt"
S1340		Closed fracture of ilium, unspecified		Patient included	Patient included, date of the event is "event_dt"	Patient included if the first event was after the UK Biobank recruitment date (Field 53), date of the event is "event_dt"
S1341		Closed fracture pelvis, ischium		Patient included	Patient included, date of the event is "event_dt"	Patient included if the first event was after the UK Biobank recruitment date (Field 53), date of the event is "event_dt"
S1342		Closed multiple disruptions of pelvis		Patient included	Patient included, date of the event is "event_dt"	Patient included if the first event was after the UK Biobank recruitment date (Field 53), date of the event is "event_dt"
S1343		Closed fracture pelvis, ischial tuberosity		Patient included	Patient included, date of the event is "event_dt"	Patient included if the first event was after the UK Biobank recruitment date (Field 53), date of the event is "event_dt"
S1344		Closed fracture pelvis, anterior superior iliac spine		Patient included	Patient included, date of the event is "event_dt"	Patient included if the first event was after the UK Biobank recruitment date (Field 53), date of the event is "event_dt"
S1345		Closed fracture pelvis, anterior inferior iliac spine		Patient included	Patient included, date of the event is "event_dt"	Patient included if the first event was after the UK Biobank recruitment date (Field 53), date of the event is "event_dt"
S1346		Closed fracture pelvis, iliac wing		Patient included	Patient included, date of the event is "event_dt"	Patient included if the first event was after the UK Biobank recruitment date (Field 53), date of the event is "event_dt"
S1347		Closed vertical fracture of ilium		Patient included	Patient included, date of the event is "event_dt"	Patient included if the first event was after the UK Biobank recruitment date (Field 53), date of the event is "event_dt"
S1348		Closed fracture dislocation of sacro-iliac joint		Patient included	Patient included, date of the event is "event_dt"	Patient included if the first event was after the UK Biobank recruitment date (Field 53), date of the event is "event_dt"

S134z		Other or multiple closed fracture of pelvis NOS		Patient included	Patient included, date of the event is "event_dt"	Patient included if the first event was after the UK Biobank recruitment date (Field 53), date of the event is "event_dt"
S150.		Multiple fractures of thoracic spine		Patient included	Patient included, date of the event is "event_dt"	Patient included if the first event was after the UK Biobank recruitment date (Field 53), date of the event is "event_dt"
S1500		Closed multiple fractures of thoracic spine		Patient included	Patient included, date of the event is "event_dt"	Patient included if the first event was after the UK Biobank recruitment date (Field 53), date of the event is "event_dt"
S210.		Closed fracture of scapula		Patient included	Patient included, date of the event is "event_dt"	Patient included if the first event was after the UK Biobank recruitment date (Field 53), date of the event is "event_dt"
S2100		Closed fracture of scapula, unspecified part		Patient included	Patient included, date of the event is "event_dt"	Patient included if the first event was after the UK Biobank recruitment date (Field 53), date of the event is "event_dt"
S2101		Closed fracture scapula, acromion		Patient included	Patient included, date of the event is "event_dt"	Patient included if the first event was after the UK Biobank recruitment date (Field 53), date of the event is "event_dt"
S2102		Closed fracture scapula, coracoid		Patient included	Patient included, date of the event is "event_dt"	Patient included if the first event was after the UK Biobank recruitment date (Field 53), date of the event is "event_dt"
S2103		Closed fracture scapula, glenoid		Patient included	Patient included, date of the event is "event_dt"	Patient included if the first event was after the UK Biobank recruitment date (Field 53), date of the event is "event_dt"
S2104		Closed fracture scapula, blade		Patient included	Patient included, date of the event is "event_dt"	Patient included if the first event was after the UK Biobank recruitment date (Field 53), date of the event is "event_dt"
S2105		Closed fracture scapula, spine		Patient included	Patient included, date of the event is "event_dt"	Patient included if the first event was after the UK Biobank recruitment date (Field 53), date of the event is "event_dt"
S2106		Closed fracture scapula, neck		Patient included	Patient included, date of the event is "event_dt"	Patient included if the first event was after the UK Biobank recruitment date (Field 53), date of the event is "event_dt"
S210z		Closed fracture of scapula NOS		Patient included	Patient included, date of the event is "event_dt"	Patient included if the first event was after the UK Biobank recruitment date (Field 53), date of the event is "event_dt"
S220.		Closed fracture of the proximal humerus		Patient included	Patient included, date of the event is "event_dt"	Patient included if the first event was after the UK Biobank recruitment date (Field 53), date of the event is "event_dt"
S2200		Closed fracture of proximal humerus, unspecified part		Patient included	Patient included, date of the event is "event_dt"	Patient included if the first event was after the UK Biobank recruitment date (Field 53), date of the event is "event_dt"
S2201		Closed fracture proximal humerus, neck		Patient included	Patient included, date of the event is "event_dt"	Patient included if the first event was after the UK Biobank recruitment date (Field 53), date of the event is "event_dt"
S2202		Closed fracture of proximal humerus, anatomical neck		Patient included	Patient included, date of the event is "event_dt"	Patient included if the first event was after the UK Biobank recruitment date (Field 53), date of the event is "event_dt"
S2203		Closed fracture proximal humerus, greater tuberosity		Patient included	Patient included, date of the event is "event_dt"	Patient included if the first event was after the UK Biobank recruitment date (Field 53), date of the event is "event_dt"
S2204		Closed fracture proximal humerus, head		Patient included	Patient included, date of the event is "event_dt"	Patient included if the first event was after the UK Biobank recruitment date (Field 53), date of the event is "event_dt"
S2205		Closed fracture of humerus, upper epiphysis		Patient included	Patient included, date of the event is "event_dt"	Patient included if the first event was after the UK Biobank recruitment date (Field 53), date of the event is "event_dt"
S2206		Closed fracture proximal humerus, three part		Patient included	Patient included, date of the event is "event_dt"	Patient included if the first event was after the UK Biobank recruitment date (Field 53), date of the event is "event_dt"
S2207		Closed fracture proximal humerus, four part		Patient included	Patient included, date of the event is "event_dt"	Patient included if the first event was after the UK Biobank recruitment date (Field 53), date of the event is "event_dt"
S220z		Closed fracture of proximal humerus not otherwise specified		Patient included	Patient included, date of the event is "event_dt"	Patient included if the first event was after the UK Biobank recruitment date (Field 53), date of the event is "event_dt"
S222.		Closed fracture of humerus, shaft or unspecified part		Patient included	Patient included, date of the event is "event_dt"	Patient included if the first event was after the UK Biobank recruitment date (Field 53), date of the event is "event_dt"
S2220		Closed fracture of humerus NOS		Patient included	Patient included, date of the event is "event_dt"	Patient included if the first event was after the UK Biobank recruitment date (Field 53), date of the event is "event_dt"
S2221		Closed fracture of humerus, shaft		Patient included	Patient included, date of the event is "event_dt"	Patient included if the first event was after the UK Biobank recruitment date (Field 53), date of the event is "event_dt"
S222z		Closed fracture of humerus, shaft or unspecified part NOS		Patient included	Patient included, date of the event is "event_dt"	Patient included if the first event was after the UK Biobank recruitment date (Field 53), date of the event is "event_dt"
S230.		Closed fracture of proximal radius and ulna		Patient included	Patient included, date of the event is "event_dt"	Patient included if the first event was after the UK Biobank recruitment date (Field 53), date of the event is "event_dt"
S2300		Closed fracture of proximal forearm, unspecified part		Patient included	Patient included, date of the event is "event_dt"	Patient included if the first event was after the UK Biobank recruitment date (Field 53), date of the event is "event_dt"
S2306		Closed fracture radius, head		Patient included	Patient included, date of the event is "event_dt"	Patient included if the first event was after the UK Biobank recruitment date (Field 53), date of the event is "event_dt"
S2307		Closed fracture radius, neck		Patient included	Patient included, date of the event is "event_dt"	Patient included if the first event was after the UK Biobank recruitment date (Field 53), date of the event is "event_dt"

S230A		Closed fracture radius and ulna, proximal		Patient included	Patient included, date of the event is "event_dt"	Patient included if the first event was after the UK Biobank recruitment date (Field 53), date of the event is "event_dt"
S230z		Closed fracture of proximal forearm not otherwise specified		Patient included	Patient included, date of the event is "event_dt"	Patient included if the first event was after the UK Biobank recruitment date (Field 53), date of the event is "event_dt"
S234.		Closed fracture of radius and ulna, lower end		Patient included	Patient included, date of the event is "event_dt"	Patient included if the first event was after the UK Biobank recruitment date (Field 53), date of the event is "event_dt"
S2340		Closed fracture of forearm, lower end, unspecified		Patient included	Patient included, date of the event is "event_dt"	Patient included if the first event was after the UK Biobank recruitment date (Field 53), date of the event is "event_dt"
S2342		Closed fracture of the distal radius, unspecified		Patient included	Patient included, date of the event is "event_dt"	Patient included if the first event was after the UK Biobank recruitment date (Field 53), date of the event is "event_dt"
S2346		Closed fracture radius and ulna, distal		Patient included	Patient included, date of the event is "event_dt"	Patient included if the first event was after the UK Biobank recruitment date (Field 53), date of the event is "event_dt"
S234C		Closed fracture distal radius, intra-articular, die-punch		Patient included	Patient included, date of the event is "event_dt"	Patient included if the first event was after the UK Biobank recruitment date (Field 53), date of the event is "event_dt"
S234D		Closed fracture distal radius, extra-articular, other type		Patient included	Patient included, date of the event is "event_dt"	Patient included if the first event was after the UK Biobank recruitment date (Field 53), date of the event is "event_dt"
S234E		Closed fracture distal radius, intra-articular, other type		Patient included	Patient included, date of the event is "event_dt"	Patient included if the first event was after the UK Biobank recruitment date (Field 53), date of the event is "event_dt"
S234z		Closed fracture of forearm, lower end, NOS		Patient included	Patient included, date of the event is "event_dt"	Patient included if the first event was after the UK Biobank recruitment date (Field 53), date of the event is "event_dt"
S300.		Closed fracture proximal femur, transcervical		Patient included	Patient included, date of the event is "event_dt"	Patient included if the first event was after the UK Biobank recruitment date (Field 53), date of the event is "event_dt"
S3000		Closed fracture proximal femur, intracapsular section, unspecified		Patient included	Patient included, date of the event is "event_dt"	Patient included if the first event was after the UK Biobank recruitment date (Field 53), date of the event is "event_dt"
S3001		Closed fracture proximal femur, transepiphyseal		Patient included	Patient included, date of the event is "event_dt"	Patient included if the first event was after the UK Biobank recruitment date (Field 53), date of the event is "event_dt"
S3002		Closed fracture proximal femur, midcervical section		Patient included	Patient included, date of the event is "event_dt"	Patient included if the first event was after the UK Biobank recruitment date (Field 53), date of the event is "event_dt"
S3003		Closed fracture proximal femur, basicervical		Patient included	Patient included, date of the event is "event_dt"	Patient included if the first event was after the UK Biobank recruitment date (Field 53), date of the event is "event_dt"
S3004		Closed fracture head of femur		Patient included	Patient included, date of the event is "event_dt"	Patient included if the first event was after the UK Biobank recruitment date (Field 53), date of the event is "event_dt"
S3005		Closed fracture proximal femur, subcapital, Garden grade unspecified		Patient included	Patient included, date of the event is "event_dt"	Patient included if the first event was after the UK Biobank recruitment date (Field 53), date of the event is "event_dt"
S3006		Closed fracture proximal femur, subcapital, Garden grade I		Patient included	Patient included, date of the event is "event_dt"	Patient included if the first event was after the UK Biobank recruitment date (Field 53), date of the event is "event_dt"
S3007		Closed fracture proximal femur, subcapital, Garden grade II		Patient included	Patient included, date of the event is "event_dt"	Patient included if the first event was after the UK Biobank recruitment date (Field 53), date of the event is "event_dt"
S3008		Closed fracture proximal femur, subcapital, Garden grade III		Patient included	Patient included, date of the event is "event_dt"	Patient included if the first event was after the UK Biobank recruitment date (Field 53), date of the event is "event_dt"
S3009		Closed fracture proximal femur, subcapital, Garden grade IV		Patient included	Patient included, date of the event is "event_dt"	Patient included if the first event was after the UK Biobank recruitment date (Field 53), date of the event is "event_dt"
S300A		Closed fracture of femur, upper epiphysis		Patient included	Patient included, date of the event is "event_dt"	Patient included if the first event was after the UK Biobank recruitment date (Field 53), date of the event is "event_dt"
S300y		Closed fracture proximal femur, other transcervical		Patient included	Patient included, date of the event is "event_dt"	Patient included if the first event was after the UK Biobank recruitment date (Field 53), date of the event is "event_dt"
S300z		Closed fracture proximal femur, transcervical, not otherwise specified		Patient included	Patient included, date of the event is "event_dt"	Patient included if the first event was after the UK Biobank recruitment date (Field 53), date of the event is "event_dt"
S302.		Closed fracture of proximal femur, pertrochanteric		Patient included	Patient included, date of the event is "event_dt"	Patient included if the first event was after the UK Biobank recruitment date (Field 53), date of the event is "event_dt"
S3020		Closed fracture of proximal femur, trochanteric section, unspecified		Patient included	Patient included, date of the event is "event_dt"	Patient included if the first event was after the UK Biobank recruitment date (Field 53), date of the event is "event_dt"
S3021		Closed fracture proximal femur, intertrochanteric, two part		Patient included	Patient included, date of the event is "event_dt"	Patient included if the first event was after the UK Biobank recruitment date (Field 53), date of the event is "event_dt"
S3022		Closed fracture proximal femur, subtrochanteric		Patient included	Patient included, date of the event is "event_dt"	Patient included if the first event was after the UK Biobank recruitment date (Field 53), date of the event is "event_dt"
S3023		Closed fracture proximal femur, intertrochanteric, comminuted		Patient included	Patient included, date of the event is "event_dt"	Patient included if the first event was after the UK Biobank recruitment date (Field 53), date of the event is "event_dt"
S3024		Closed fracture of femur, intertrochanteric		Patient included	Patient included, date of the event is "event_dt"	Patient included if the first event was after the UK Biobank recruitment date (Field 53), date of the event is "event_dt"

S302z		Closed fracture of proximal femur, pertrochanteric section, not otherwise specified		Patient included	Patient included, date of the event is "event_dt"	Patient included if the first event was after the UK Biobank recruitment date (Field 53), date of the event is "event_dt"
S304.		Pertrochanteric fracture		Patient included	Patient included, date of the event is "event_dt"	Patient included if the first event was after the UK Biobank recruitment date (Field 53), date of the event is "event_dt"
S305.		Subtrochanteric fracture		Patient included	Patient included, date of the event is "event_dt"	Patient included if the first event was after the UK Biobank recruitment date (Field 53), date of the event is "event_dt"
S30w.		Closed fracture of unspecified proximal femur		Patient included	Patient included, date of the event is "event_dt"	Patient included if the first event was after the UK Biobank recruitment date (Field 53), date of the event is "event_dt"
S30y.		Closed fracture of neck of femur NOS		Patient included	Patient included, date of the event is "event_dt"	Patient included if the first event was after the UK Biobank recruitment date (Field 53), date of the event is "event_dt"
S31..		Other fracture of femur		Patient included	Patient included, date of the event is "event_dt"	Patient included if the first event was after the UK Biobank recruitment date (Field 53), date of the event is "event_dt"
S310.		Closed fracture of femur, shaft or unspecified part		Patient included	Patient included, date of the event is "event_dt"	Patient included if the first event was after the UK Biobank recruitment date (Field 53), date of the event is "event_dt"
S3100		Closed fracture of femur, unspecified part		Patient included	Patient included, date of the event is "event_dt"	Patient included if the first event was after the UK Biobank recruitment date (Field 53), date of the event is "event_dt"
S3101		Closed fracture shaft of femur		Patient included	Patient included, date of the event is "event_dt"	Patient included if the first event was after the UK Biobank recruitment date (Field 53), date of the event is "event_dt"
S312.		Closed fracture distal femur		Patient included	Patient included, date of the event is "event_dt"	Patient included if the first event was after the UK Biobank recruitment date (Field 53), date of the event is "event_dt"
S3120		Closed fracture of distal femur, unspecified		Patient included	Patient included, date of the event is "event_dt"	Patient included if the first event was after the UK Biobank recruitment date (Field 53), date of the event is "event_dt"
S3122		Closed fracture of femur, lower epiphysis		Patient included	Patient included, date of the event is "event_dt"	Patient included if the first event was after the UK Biobank recruitment date (Field 53), date of the event is "event_dt"
S3123		Closed fracture distal femur, supracondylar		Patient included	Patient included, date of the event is "event_dt"	Patient included if the first event was after the UK Biobank recruitment date (Field 53), date of the event is "event_dt"
S3124		Closed fracture distal femur, medial condyle		Patient included	Patient included, date of the event is "event_dt"	Patient included if the first event was after the UK Biobank recruitment date (Field 53), date of the event is "event_dt"
S3125		Closed fracture distal femur, lateral condyle		Patient included	Patient included, date of the event is "event_dt"	Patient included if the first event was after the UK Biobank recruitment date (Field 53), date of the event is "event_dt"
S3126		Closed fracture distal femur, bicondylar (T-Y fracture)		Patient included	Patient included, date of the event is "event_dt"	Patient included if the first event was after the UK Biobank recruitment date (Field 53), date of the event is "event_dt"
S312x		Closed fracture distal femur, comminuted/intra-articular		Patient included	Patient included, date of the event is "event_dt"	Patient included if the first event was after the UK Biobank recruitment date (Field 53), date of the event is "event_dt"
S312z		Closed fracture of distal femur not otherwise specified		Patient included	Patient included, date of the event is "event_dt"	Patient included if the first event was after the UK Biobank recruitment date (Field 53), date of the event is "event_dt"
S4A0.		Closed fracture-dislocation shoulder		Patient included	Patient included, date of the event is "event_dt"	Patient included if the first event was after the UK Biobank recruitment date (Field 53), date of the event is "event_dt"
S4A00		Closed fracture-dislocation shoulder joint		Patient included	Patient included, date of the event is "event_dt"	Patient included if the first event was after the UK Biobank recruitment date (Field 53), date of the event is "event_dt"
S4A01		Closed fracture-dislocation acromio-clavicular joint		Patient included	Patient included, date of the event is "event_dt"	Patient included if the first event was after the UK Biobank recruitment date (Field 53), date of the event is "event_dt"
S4A2.		Closed fracture-subluxation shoulder		Patient included	Patient included, date of the event is "event_dt"	Patient included if the first event was after the UK Biobank recruitment date (Field 53), date of the event is "event_dt"
S4A20		Closed fracture-subluxation shoulder joint		Patient included	Patient included, date of the event is "event_dt"	Patient included if the first event was after the UK Biobank recruitment date (Field 53), date of the event is "event_dt"
S4A21		Closed fracture-subluxation acromio-clavicular joint		Patient included	Patient included, date of the event is "event_dt"	Patient included if the first event was after the UK Biobank recruitment date (Field 53), date of the event is "event_dt"
S4B0.		Closed fracture-dislocation elbow		Patient included	Patient included, date of the event is "event_dt"	Patient included if the first event was after the UK Biobank recruitment date (Field 53), date of the event is "event_dt"
S4B00		Closed fracture-dislocation elbow joint		Patient included	Patient included, date of the event is "event_dt"	Patient included if the first event was after the UK Biobank recruitment date (Field 53), date of the event is "event_dt"
S4B01		Closed fracture-dislocation superior radio-ulnar joint		Patient included	Patient included, date of the event is "event_dt"	Patient included if the first event was after the UK Biobank recruitment date (Field 53), date of the event is "event_dt"
S4B2.		Closed fracture-subluxation elbow		Patient included	Patient included, date of the event is "event_dt"	Patient included if the first event was after the UK Biobank recruitment date (Field 53), date of the event is "event_dt"
S4B20		Closed fracture-subluxation elbow joint		Patient included	Patient included, date of the event is "event_dt"	Patient included if the first event was after the UK Biobank recruitment date (Field 53), date of the event is "event_dt"
S4B21		Closed fracture-subluxation superior radio-ulnar joint		Patient included	Patient included, date of the event is "event_dt"	Patient included if the first event was after the UK Biobank recruitment date (Field 53), date of the event is "event_dt"

S4C..		Fracture-dislocation or subluxation of wrist		Patient included	Patient included, date of the event is "event_dt"	Patient included if the first event was after the UK Biobank recruitment date (Field 53), date of the event is "event_dt"
S4C0.		Closed fracture dislocation of wrist		Patient included	Patient included, date of the event is "event_dt"	Patient included if the first event was after the UK Biobank recruitment date (Field 53), date of the event is "event_dt"
S4C00		Closed fracture-dislocation distal radio-ulnar joint		Patient included	Patient included, date of the event is "event_dt"	Patient included if the first event was after the UK Biobank recruitment date (Field 53), date of the event is "event_dt"
S4C01		Closed fracture-dislocation radiocarpal joint		Patient included	Patient included, date of the event is "event_dt"	Patient included if the first event was after the UK Biobank recruitment date (Field 53), date of the event is "event_dt"
S4C02		Closed fracture-dislocation mid carpal		Patient included	Patient included, date of the event is "event_dt"	Patient included if the first event was after the UK Biobank recruitment date (Field 53), date of the event is "event_dt"
S4C03		Closed fracture-dislocation, carpometacarpal joint		Patient included	Patient included, date of the event is "event_dt"	Patient included if the first event was after the UK Biobank recruitment date (Field 53), date of the event is "event_dt"
S4E..		Fracture-dislocation or subluxation hip		Patient included	Patient included, date of the event is "event_dt"	Patient included if the first event was after the UK Biobank recruitment date (Field 53), date of the event is "event_dt"
S4E0.		Closed fracture-dislocation, hip joint		Patient included	Patient included, date of the event is "event_dt"	Patient included if the first event was after the UK Biobank recruitment date (Field 53), date of the event is "event_dt"
S4E2.		Closed fracture-subluxation, hip joint		Patient included	Patient included, date of the event is "event_dt"	Patient included if the first event was after the UK Biobank recruitment date (Field 53), date of the event is "event_dt"
S4J01		Closed fracture-dislocation of pelvis		Patient included	Patient included, date of the event is "event_dt"	Patient included if the first event was after the UK Biobank recruitment date (Field 53), date of the event is "event_dt"
S4J21		Closed fracture-subluxation of pelvis		Patient included	Patient included, date of the event is "event_dt"	Patient included if the first event was after the UK Biobank recruitment date (Field 53), date of the event is "event_dt"
Syu42		[X]Multiple fractures of clavicle, scapula and humerus		Patient included	Patient included, date of the event is "event_dt"	Patient included if the first event was after the UK Biobank recruitment date (Field 53), date of the event is "event_dt"
S10..		Fracture of spine without mention of spinal cord injury		Patient included	Patient included, date of the event is "event_dt"	Patient included if the first event was after the UK Biobank recruitment date (Field 53), date of the event is "event_dt"
S10B.		Fracture of lumbar spine and pelvis		Patient included	Patient included, date of the event is "event_dt"	Patient included if the first event was after the UK Biobank recruitment date (Field 53), date of the event is "event_dt"
S10B0		Fracture of lumbar vertebra		Patient included	Patient included, date of the event is "event_dt"	Patient included if the first event was after the UK Biobank recruitment date (Field 53), date of the event is "event_dt"
S10B1		Fracture of sacrum		Patient included	Patient included, date of the event is "event_dt"	Patient included if the first event was after the UK Biobank recruitment date (Field 53), date of the event is "event_dt"
S10B2		Fracture of coccyx		Patient included	Patient included, date of the event is "event_dt"	Patient included if the first event was after the UK Biobank recruitment date (Field 53), date of the event is "event_dt"
S10B3		Fracture of ilium		Patient included	Patient included, date of the event is "event_dt"	Patient included if the first event was after the UK Biobank recruitment date (Field 53), date of the event is "event_dt"
S10B4		Fracture of acetabulum		Patient included	Patient included, date of the event is "event_dt"	Patient included if the first event was after the UK Biobank recruitment date (Field 53), date of the event is "event_dt"
S10B5		Fracture of pubis		Patient included	Patient included, date of the event is "event_dt"	Patient included if the first event was after the UK Biobank recruitment date (Field 53), date of the event is "event_dt"
S11..		Fracture of spine with spinal cord lesion		Patient included	Patient included, date of the event is "event_dt"	Patient included if the first event was after the UK Biobank recruitment date (Field 53), date of the event is "event_dt"
S127.		Fracture of rib		Patient included	Patient included, date of the event is "event_dt"	Patient included if the first event was after the UK Biobank recruitment date (Field 53), date of the event is "event_dt"
S13..		Fracture or disruption of pelvis		Patient included	Patient included, date of the event is "event_dt"	Patient included if the first event was after the UK Biobank recruitment date (Field 53), date of the event is "event_dt"
S15..		Fracture of thoracic vertebra		Patient included	Patient included, date of the event is "event_dt"	Patient included if the first event was after the UK Biobank recruitment date (Field 53), date of the event is "event_dt"
S21..		Fracture of scapula		Patient included	Patient included, date of the event is "event_dt"	Patient included if the first event was after the UK Biobank recruitment date (Field 53), date of the event is "event_dt"
S21z.		Fracture of scapula NOS		Patient included	Patient included, date of the event is "event_dt"	Patient included if the first event was after the UK Biobank recruitment date (Field 53), date of the event is "event_dt"
S22..		Fracture of humerus		Patient included	Patient included, date of the event is "event_dt"	Patient included if the first event was after the UK Biobank recruitment date (Field 53), date of the event is "event_dt"
S226.		Fracture of upper end of humerus		Patient included	Patient included, date of the event is "event_dt"	Patient included if the first event was after the UK Biobank recruitment date (Field 53), date of the event is "event_dt"
S227.		Fracture of shaft of humerus		Patient included	Patient included, date of the event is "event_dt"	Patient included if the first event was after the UK Biobank recruitment date (Field 53), date of the event is "event_dt"
S228.		Fracture of lower end of humerus		Patient included	Patient included, date of the event is "event_dt"	Patient included if the first event was after the UK Biobank recruitment date (Field 53), date of the event is "event_dt"

S22z.		Fracture of humerus NOS		Patient included	Patient included, date of the event is "event_dt"	Patient included if the first event was after the UK Biobank recruitment date (Field 53), date of the event is "event_dt"
S23..		Fracture of radius and ulna		Patient included	Patient included, date of the event is "event_dt"	Patient included if the first event was after the UK Biobank recruitment date (Field 53), date of the event is "event_dt"
S236.		Fracture of upper end of ulna		Patient included	Patient included, date of the event is "event_dt"	Patient included if the first event was after the UK Biobank recruitment date (Field 53), date of the event is "event_dt"
S237.		Fracture of upper end of radius		Patient included	Patient included, date of the event is "event_dt"	Patient included if the first event was after the UK Biobank recruitment date (Field 53), date of the event is "event_dt"
S238.		Fracture of shaft of ulna		Patient included	Patient included, date of the event is "event_dt"	Patient included if the first event was after the UK Biobank recruitment date (Field 53), date of the event is "event_dt"
S239.		Fracture of shaft of radius		Patient included	Patient included, date of the event is "event_dt"	Patient included if the first event was after the UK Biobank recruitment date (Field 53), date of the event is "event_dt"
S23A.		Fracture of shafts of both ulna and radius		Patient included	Patient included, date of the event is "event_dt"	Patient included if the first event was after the UK Biobank recruitment date (Field 53), date of the event is "event_dt"
S23B.		Fracture of lower end of radius		Patient included	Patient included, date of the event is "event_dt"	Patient included if the first event was after the UK Biobank recruitment date (Field 53), date of the event is "event_dt"
S23C.		Fracture of lower end of both ulna and radius		Patient included	Patient included, date of the event is "event_dt"	Patient included if the first event was after the UK Biobank recruitment date (Field 53), date of the event is "event_dt"
S23x.		Closed fracture of radius and ulna, unspecified part		Patient included	Patient included, date of the event is "event_dt"	Patient included if the first event was after the UK Biobank recruitment date (Field 53), date of the event is "event_dt"
S23x1		Fracture of radius NOS		Patient included	Patient included, date of the event is "event_dt"	Patient included if the first event was after the UK Biobank recruitment date (Field 53), date of the event is "event_dt"
S23z.		Fracture of radius and ulna, NOS		Patient included	Patient included, date of the event is "event_dt"	Patient included if the first event was after the UK Biobank recruitment date (Field 53), date of the event is "event_dt"
S242.		Fracture at wrist and hand level		Patient included	Patient included, date of the event is "event_dt"	Patient included if the first event was after the UK Biobank recruitment date (Field 53), date of the event is "event_dt"
S30..		Fracture of neck of femur		Patient included	Patient included, date of the event is "event_dt"	Patient included if the first event was after the UK Biobank recruitment date (Field 53), date of the event is "event_dt"
S314.		Fracture of shaft of femur		Patient included	Patient included, date of the event is "event_dt"	Patient included if the first event was after the UK Biobank recruitment date (Field 53), date of the event is "event_dt"
S315.		Fracture of lower end of femur		Patient included	Patient included, date of the event is "event_dt"	Patient included if the first event was after the UK Biobank recruitment date (Field 53), date of the event is "event_dt"
S31z.		Fracture of femur, NOS		Patient included	Patient included, date of the event is "event_dt"	Patient included if the first event was after the UK Biobank recruitment date (Field 53), date of the event is "event_dt"
S4A..		Fracture-dislocation or subluxation shoulder		Patient included	Patient included, date of the event is "event_dt"	Patient included if the first event was after the UK Biobank recruitment date (Field 53), date of the event is "event_dt"
S4B..		Fracture-dislocation or subluxation elbow		Patient included	Patient included, date of the event is "event_dt"	Patient included if the first event was after the UK Biobank recruitment date (Field 53), date of the event is "event_dt"
S4C..		Fracture-dislocation or subluxation of wrist		Patient included	Patient included, date of the event is "event_dt"	Patient included if the first event was after the UK Biobank recruitment date (Field 53), date of the event is "event_dt"
S4E..		Fracture-dislocation or subluxation hip		Patient included	Patient included, date of the event is "event_dt"	Patient included if the first event was after the UK Biobank recruitment date (Field 53), date of the event is "event_dt"
SR11.		Fractures involving thorax with lower back and pelvis		Patient included	Patient included, date of the event is "event_dt"	Patient included if the first event was after the UK Biobank recruitment date (Field 53), date of the event is "event_dt"
SR16.		Fractures involving thorax with lower back and pelvis with limb(s)		Patient included	Patient included, date of the event is "event_dt"	Patient included if the first event was after the UK Biobank recruitment date (Field 53), date of the event is "event_dt"
Syu34		[X]Fracture of other and unspecified parts of lumbar spine and pelvis		Patient included	Patient included, date of the event is "event_dt"	Patient included if the first event was after the UK Biobank recruitment date (Field 53), date of the event is "event_dt"
Syu43		[X]Fracture of other parts of shoulder and upper arm		Patient included	Patient included, date of the event is "event_dt"	Patient included if the first event was after the UK Biobank recruitment date (Field 53), date of the event is "event_dt"
Syu44		[X]Fracture of shoulder and upper arm, unspecified		Patient included	Patient included, date of the event is "event_dt"	Patient included if the first event was after the UK Biobank recruitment date (Field 53), date of the event is "event_dt"
Syu53		[X]Fracture of other parts of forearm		Patient included	Patient included, date of the event is "event_dt"	Patient included if the first event was after the UK Biobank recruitment date (Field 53), date of the event is "event_dt"
Syu54		[X]Fracture of forearm, unspecified		Patient included	Patient included, date of the event is "event_dt"	Patient included if the first event was after the UK Biobank recruitment date (Field 53), date of the event is "event_dt"
Syu65		[X]Fracture of other and unspecified parts of wrist and hand		Patient included	Patient included, date of the event is "event_dt"	Patient included if the first event was after the UK Biobank recruitment date (Field 53), date of the event is "event_dt"
S2341		Closed Colles' fracture		Patient included	Patient included, date of the event is "event_dt"	Patient included if the first event was after the UK Biobank recruitment date (Field 53), date of the event is "event_dt"

S2351		Open Colles' fracture		Patient included	Patient included, date of the event is "event_dt"	Patient included if the first event was after the UK Biobank recruitment date (Field 53), date of the event is "event_dt"
**Coronary artery disease outcome**						
Source data: Linkage to HES						
**ICD10**	**Code**	**Description**	**Extra information**	**As binary utcome**	**As survival outcome (since birth date)**	**As survival outcome (since enrolment date)**
I251		Atherosclerotic heart disease		Patient included	Patient included, date of the event is the Minimum between "epistart" and "admidate	Patient included if the first event was after the UK Biobank recruitment date (Field 53), date of the event is the minimum between "epistart" and "admidate".
Source data: Linkage to GP						
**Read2 code**	**Code**	**Description**	**Extra information**	**As binary utcome**	**As survival outcome (since birth date)**	**As survival outcome (since enrolment date)**
G340.		Coronary atherosclerosis		Patient included	Patient included, date of the event is "event_dt"	Patient included if the first event was after the UK Biobank recruitment date (Field 53), date of the event is "event_dt"
G3400		Single coronary vessel disease		Patient included	Patient included, date of the event is "event_dt"	Patient included if the first event was after the UK Biobank recruitment date (Field 53), date of the event is "event_dt"
G3401		Double coronary vessel disease		Patient included	Patient included, date of the event is "event_dt"	Patient included if the first event was after the UK Biobank recruitment date (Field 53), date of the event is "event_dt"
G704.		Non-obstructive coronary atherosclerosis		Patient included	Patient included, date of the event is "event_dt"	Patient included if the first event was after the UK Biobank recruitment date (Field 53), date of the event is "event_dt"
**Myocardial infarction outcome**						
Source data: UK Biobank						
**Field**	**Code**	**Description**	**Extra information**	**As binary utcome**	**As survival outcome (since birth date)**	**As survival outcome (since enrolment date)**
3894		Age heart attack diagnosed		Patient included	Patient included, years at the event is provided by the same field	Patient excluded
20002	1075	Non-cancer illness code, self-reported - Heart attack/Myocardial infarction		Patient included	Patient included, years at the event is Field 20009 (Interpolated year/age of the event)	Patient excluded
Source data: Linkage to HES						
**ICD10**	**Code**	**Description**	**Extra information**	**As binary utcome**	**As survival outcome (since birth date)**	**As survival outcome (since enrolment date)**
I21		Acute myocardial infarction		Patient included	Patient included, date of the event is the minimum between "epistart" and "admidate"	Patient included if the first event was after the UK Biobank recruitment date (Field 53), date of the event is the minimum between "epistart" and "admidate"
I210		Acute transmural myocardial infarction of anterior wall		Patient included	Patient included, date of the event is the minimum between "epistart" and "admidate"	Patient included if the first event was after the UK Biobank recruitment date (Field 53), date of the event is the minimum between "epistart" and "admidate"
I211		Acute transmural myocardial infarction of inferior wall		Patient included	Patient included, date of the event is the minimum between "epistart" and "admidate"	Patient included if the first event was after the UK Biobank recruitment date (Field 53), date of the event is the minimum between "epistart" and "admidate"
I212		Acute transmural myocardial infarction of other sites		Patient included	Patient included, date of the event is the minimum between "epistart" and "admidate"	Patient included if the first event was after the UK Biobank recruitment date (Field 53), date of the event is the minimum between "epistart" and "admidate"
I213		Acute transmural myocardial infarction of unspecified site		Patient included	Patient included, date of the event is the minimum between "epistart" and "admidate"	Patient included if the first event was after the UK Biobank recruitment date (Field 53), date of the event is the minimum between "epistart" and "admidate"
I214		Acute subendocardial myocardial infarction		Patient included	Patient included, date of the event is the minimum between "epistart" and "admidate"	Patient included if the first event was after the UK Biobank recruitment date (Field 53), date of the event is the minimum between "epistart" and "admidate"
I219		Acute myocardial infarction, unspecified		Patient included	Patient included, date of the event is the minimum between "epistart" and "admidate"	Patient included if the first event was after the UK Biobank recruitment date (Field 53), date of the event is the minimum between "epistart" and "admidate"
I22		Subsequent myocardial infarction		Patient included	Patient included, date of the event is the minimum between "epistart" and "admidate"	Patient included if the first event was after the UK Biobank recruitment date (Field 53), date of the event is the minimum between "epistart" and "admidate"
I220		Subsequent myocardial infarction of anterior wall		Patient included	Patient included, date of the event is the minimum between "epistart" and "admidate"	Patient included if the first event was after the UK Biobank recruitment date (Field 53), date of the event is the minimum between "epistart" and "admidate"
I221		Subsequent myocardial infarction of inferior wall		Patient included	Patient included, date of the event is the minimum between "epistart" and "admidate"	Patient included if the first event was after the UK Biobank recruitment date (Field 53), date of the event is the minimum between "epistart" and "admidate"
I228		Subsequent myocardial infarction of other sites		Patient included	Patient included, date of the event is the minimum between "epistart" and "admidate"	Patient included if the first event was after the UK Biobank recruitment date (Field 53), date of the event is the minimum between "epistart" and "admidate"

I229		Subsequent myocardial infarction of unspecified site		Patient included	Patient included, date of the event is the minimum between "epistart" and "admidate"	Patient included if the first event was after the UK Biobank recruitment date (Field 53), date of the event is the minimum between "epistart" and "admidate"
I23		Certain current complications following acute myocardial infarction		Patient included	Patient included, date of the event is the minimum between "epistart" and "admidate"	Patient included if the first event was after the UK Biobank recruitment date (Field 53), date of the event is the minimum between "epistart" and "admidate"
I230		Haemopericardium as current complication following acute myocardial infarction		Patient included	Patient included, date of the event is the minimum between "epistart" and "admidate"	Patient included if the first event was after the UK Biobank recruitment date (Field 53), date of the event is the minimum between "epistart" and "admidate"
I231		Atrial septal defect as current complication following acute myocardial infarction		Patient included	Patient included, date of the event is the minimum between "epistart" and "admidate"	Patient included if the first event was after the UK Biobank recruitment date (Field 53), date of the event is the minimum between "epistart" and "admidate"
I232		Ventricular septal defect as current complication following acute myocardial infarction		Patient included	Patient included, date of the event is the minimum between "epistart" and "admidate"	Patient included if the first event was after the UK Biobank recruitment date (Field 53), date of the event is the minimum between "epistart" and "admidate"
I233		Rupture of cardiac wall without haemopericardium as current complication following acute myocardial infarction		Patient included	Patient included, date of the event is the minimum between "epistart" and "admidate"	Patient included if the first event was after the UK Biobank recruitment date (Field 53), date of the event is the minimum between "epistart" and "admidate"
I234		Rupture of chordae tendineae as current complication following acute myocardial infarction		Patient included	Patient included, date of the event is the minimum between "epistart" and "admidate"	Patient included if the first event was after the UK Biobank recruitment date (Field 53), date of the event is the minimum between "epistart" and "admidate"
I235		Rupture of papillary muscle as current complication following acute myocardial infarction		Patient included	Patient included, date of the event is the minimum between "epistart" and "admidate"	Patient included if the first event was after the UK Biobank recruitment date (Field 53), date of the event is the minimum between "epistart" and "admidate"
I236		Thrombosis of atrium, auricular appendage, and ventricle as current complications following acute myocardial infarction		Patient included	Patient included, date of the event is the minimum between "epistart" and "admidate"	Patient included if the first event was after the UK Biobank recruitment date (Field 53), date of the event is the minimum between "epistart" and "admidate"
I238		Other current complications following acute myocardial infarction		Patient included	Patient included, date of the event is the minimum between "epistart" and "admidate"	Patient included if the first event was after the UK Biobank recruitment date (Field 53), date of the event is the minimum between "epistart" and "admidate"
Source data: Linkage to GP						
**Read2 code**	**Code**	**Description**	**Extra information**	**As binary utcome**	**As survival outcome (since birth date)**	**As survival outcome (since enrolment date)**
G300.		Acute anterolateral infarction		Patient included	Patient included, date of the event is "event_dt"	Patient included if the first event was after the UK Biobank recruitment date (Field 53), date of the event is "event_dt"
G301.		Other specified anterior myocardial infarction		Patient included	Patient included, date of the event is "event_dt"	Patient included if the first event was after the UK Biobank recruitment date (Field 53), date of the event is "event_dt"
G3010		Acute anteroapical infarction		Patient included	Patient included, date of the event is "event_dt"	Patient included if the first event was after the UK Biobank recruitment date (Field 53), date of the event is "event_dt"
G3011		Acute anteroseptal infarction		Patient included	Patient included, date of the event is "event_dt"	Patient included if the first event was after the UK Biobank recruitment date (Field 53), date of the event is "event_dt"
G301z		Anterior myocardial infarction NOS		Patient included	Patient included, date of the event is "event_dt"	Patient included if the first event was after the UK Biobank recruitment date (Field 53), date of the event is "event_dt"
G302.		Acute inferolateral infarction		Patient included	Patient included, date of the event is "event_dt"	Patient included if the first event was after the UK Biobank recruitment date (Field 53), date of the event is "event_dt"
G303.		Acute inferoposterior infarction		Patient included	Patient included, date of the event is "event_dt"	Patient included if the first event was after the UK Biobank recruitment date (Field 53), date of the event is "event_dt"
G304.		Posterior myocardial infarction NOS		Patient included	Patient included, date of the event is "event_dt"	Patient included if the first event was after the UK Biobank recruitment date (Field 53), date of the event is "event_dt"
G305.		Lateral myocardial infarction NOS		Patient included	Patient included, date of the event is "event_dt"	Patient included if the first event was after the UK Biobank recruitment date (Field 53), date of the event is "event_dt"
G306.		True posterior myocardial infarction		Patient included	Patient included, date of the event is "event_dt"	Patient included if the first event was after the UK Biobank recruitment date (Field 53), date of the event is "event_dt"
G307.		Acute subendocardial infarction		Patient included	Patient included, date of the event is "event_dt"	Patient included if the first event was after the UK Biobank recruitment date (Field 53), date of the event is "event_dt"
G3070		Acute non-Q wave infarction		Patient included	Patient included, date of the event is "event_dt"	Patient included if the first event was after the UK Biobank recruitment date (Field 53), date of the event is "event_dt"
G3071		Acute non-ST segment elevation myocardial infarction		Patient included	Patient included, date of the event is "event_dt"	Patient included if the first event was after the UK Biobank recruitment date (Field 53), date of the event is "event_dt"

G308.		Inferior myocardial infarction NOS		Patient included	Patient included, date of the event is "event_dt"	Patient included if the first event was after the UK Biobank recruitment date (Field 53), date of the event is "event_dt"
G309.		Acute Q-wave infarct		Patient included	Patient included, date of the event is "event_dt"	Patient included if the first event was after the UK Biobank recruitment date (Field 53), date of the event is "event_dt"
G30A.		Mural thrombosis		Patient included	Patient included, date of the event is "event_dt"	Patient included if the first event was after the UK Biobank recruitment date (Field 53), date of the event is "event_dt"
G30B.		Acute posterolateral myocardial infarction		Patient included	Patient included, date of the event is "event_dt"	Patient included if the first event was after the UK Biobank recruitment date (Field 53), date of the event is "event_dt"
G30X.		Acute transmural myocardial infarction of unspecified site		Patient included	Patient included, date of the event is "event_dt"	Patient included if the first event was after the UK Biobank recruitment date (Field 53), date of the event is "event_dt"
G30X0		Acute ST segment elevation myocardial infarction		Patient included	Patient included, date of the event is "event_dt"	Patient included if the first event was after the UK Biobank recruitment date (Field 53), date of the event is "event_dt"
G30y.		Other acute myocardial infarction		Patient included	Patient included, date of the event is "event_dt"	Patient included if the first event was after the UK Biobank recruitment date (Field 53), date of the event is "event_dt"
G30y0		Acute atrial infarction		Patient included	Patient included, date of the event is "event_dt"	Patient included if the first event was after the UK Biobank recruitment date (Field 53), date of the event is "event_dt"
G30y1		Acute papillary muscle infarction		Patient included	Patient included, date of the event is "event_dt"	Patient included if the first event was after the UK Biobank recruitment date (Field 53), date of the event is "event_dt"
G30y2		Acute septal infarction		Patient included	Patient included, date of the event is "event_dt"	Patient included if the first event was after the UK Biobank recruitment date (Field 53), date of the event is "event_dt"
G30yz		Other acute myocardial infarction NOS		Patient included	Patient included, date of the event is "event_dt"	Patient included if the first event was after the UK Biobank recruitment date (Field 53), date of the event is "event_dt"
G30z.		Acute myocardial infarction NOS		Patient included	Patient included, date of the event is "event_dt"	Patient included if the first event was after the UK Biobank recruitment date (Field 53), date of the event is "event_dt"
G35..		Subsequent myocardial infarction		Patient included	Patient included, date of the event is "event_dt"	Patient included if the first event was after the UK Biobank recruitment date (Field 53), date of the event is "event_dt"
G350.		Subsequent myocardial infarction of anterior wall		Patient included	Patient included, date of the event is "event_dt"	Patient included if the first event was after the UK Biobank recruitment date (Field 53), date of the event is "event_dt"
G351.		Subsequent myocardial infarction of inferior wall		Patient included	Patient included, date of the event is "event_dt"	Patient included if the first event was after the UK Biobank recruitment date (Field 53), date of the event is "event_dt"
G353.		Subsequent myocardial infarction of other sites		Patient included	Patient included, date of the event is "event_dt"	Patient included if the first event was after the UK Biobank recruitment date (Field 53), date of the event is "event_dt"
G35X.		Subsequent myocardial infarction of unspecified site		Patient included	Patient included, date of the event is "event_dt"	Patient included if the first event was after the UK Biobank recruitment date (Field 53), date of the event is "event_dt"
G36..		Certain current complications following acute myocardial infarction		Patient included	Patient included, date of the event is "event_dt"	Patient included if the first event was after the UK Biobank recruitment date (Field 53), date of the event is "event_dt"
G360.		Haemopericardium as current complication following acute myocardial infarction		Patient included	Patient included, date of the event is "event_dt"	Patient included if the first event was after the UK Biobank recruitment date (Field 53), date of the event is "event_dt"
G361.		Atrial septal defect as current complication following acute myocardial infarction		Patient included	Patient included, date of the event is "event_dt"	Patient included if the first event was after the UK Biobank recruitment date (Field 53), date of the event is "event_dt"
G362.		Ventricular septal defect as current complication following acute myocardial infarction		Patient included	Patient included, date of the event is "event_dt"	Patient included if the first event was after the UK Biobank recruitment date (Field 53), date of the event is "event_dt"
G363.		Rupture of cardiac wall without haemopericardium as current complication following acute myocardial infarction		Patient included	Patient included, date of the event is "event_dt"	Patient included if the first event was after the UK Biobank recruitment date (Field 53), date of the event is "event_dt"
G364.		Rupture of chordae tendinae as current complication following acute myocardial infarction		Patient included	Patient included, date of the event is "event_dt"	Patient included if the first event was after the UK Biobank recruitment date (Field 53), date of the event is "event_dt"
G365.		Rupture of papillary muscle as current complication following acute myocardial infarction		Patient included	Patient included, date of the event is "event_dt"	Patient included if the first event was after the UK Biobank recruitment date (Field 53), date of the event is "event_dt"
G366.		Thrombosis of atrium, auricular appendage, and ventricle as current complications following acute myocardial infarction		Patient included	Patient included, date of the event is "event_dt"	Patient included if the first event was after the UK Biobank recruitment date (Field 53), date of the event is "event_dt"
G38..		Postoperative myocardial infarction		Patient included	Patient included, date of the event is "event_dt"	Patient included if the first event was after the UK Biobank recruitment date (Field 53), date of the event is "event_dt"

G380.		Postoperative transmural myocardial infarction of anterior wall		Patient included	Patient included, date of the event is "event_dt"	Patient included if the first event was after the UK Biobank recruitment date (Field 53), date of the event is "event_dt"
G381.		Postoperative transmural myocardial infarction of inferior wall		Patient included	Patient included, date of the event is "event_dt"	Patient included if the first event was after the UK Biobank recruitment date (Field 53), date of the event is "event_dt"
G382.		Postoperative transmural myocardial infarction of other sites		Patient included	Patient included, date of the event is "event_dt"	Patient included if the first event was after the UK Biobank recruitment date (Field 53), date of the event is "event_dt"
G383.		Postoperative transmural myocardial infarction of unspecified site		Patient included	Patient included, date of the event is "event_dt"	Patient included if the first event was after the UK Biobank recruitment date (Field 53), date of the event is "event_dt"
G384.		Postoperative subendocardial myocardial infarction		Patient included	Patient included, date of the event is "event_dt"	Patient included if the first event was after the UK Biobank recruitment date (Field 53), date of the event is "event_dt"
G38z.		Postoperative myocardial infarction, unspecified		Patient included	Patient included, date of the event is "event_dt"	Patient included if the first event was after the UK Biobank recruitment date (Field 53), date of the event is "event_dt"
G501.		Post infarction pericarditis		Patient included	Patient included, date of the event is "event_dt"	Patient included if the first event was after the UK Biobank recruitment date (Field 53), date of the event is "event_dt"
Gyu31		[X]Other current complications following acute myocardial infarction		Patient included	Patient included, date of the event is "event_dt"	Patient included if the first event was after the UK Biobank recruitment date (Field 53), date of the event is "event_dt"
Gyu34		[X]Acute transmural myocardial infarction of unspecified site		Patient included	Patient included, date of the event is "event_dt"	Patient included if the first event was after the UK Biobank recruitment date (Field 53), date of the event is "event_dt"
Gyu35		[X]Subsequent myocardial infarction of other sites		Patient included	Patient included, date of the event is "event_dt"	Patient included if the first event was after the UK Biobank recruitment date (Field 53), date of the event is "event_dt"
Gyu36		[X]Subsequent myocardial infarction of unspecified site		Patient included	Patient included, date of the event is "event_dt"	Patient included if the first event was after the UK Biobank recruitment date (Field 53), date of the event is "event_dt"
**Ischaemic stroke outcome**						
Source data: UK Biobank						
**Field**	**Code**	**Description**	**Extra information**	**As binary utcome**	**As survival outcome (since birth date)**	**As survival outcome (since enrolment date)**
20002	1583	Ischaemic stroke		Patient included	Patient included, years at the event is provided by Field 20009 (Interpolated year/age of the event)	Patient excluded
Source data: Linkage to HES						
**ICD10**	**Code**	**Description**	**Extra information**	**As binary utcome**	**As survival outcome (since birth date)**	**As survival outcome (since enrolment date)**
I63		I63 Cerebral infarction		Patient included	Patient included, date of the event is the minimum between "epistart" and "admidate"	Patient included if the first event was after the UK Biobank recruitment date (Field 53), date of the event is the minimum between "epistart" and "admidate"
I630		I63.0 Cerebral infarction due to thrombosis of precerebral arteries		Patient included	Patient included, date of the event is the minimum between "epistart" and "admidate"	Patient included if the first event was after the UK Biobank recruitment date (Field 53), date of the event is the minimum between "epistart" and "admidate"
I631		I63.1 Cerebral infarction due to embolism of precerebral arteries		Patient included	Patient included, date of the event is the minimum between "epistart" and "admidate"	Patient included if the first event was after the UK Biobank recruitment date (Field 53), date of the event is the minimum between "epistart" and "admidate"
I632		I63.2 Cerebral infarction due to unspecified occlusion or stenosis of precerebral arteries		Patient included	Patient included, date of the event is the minimum between "epistart" and "admidate"	Patient included if the first event was after the UK Biobank recruitment date (Field 53), date of the event is the minimum between "epistart" and "admidate"
I633		I63.3 Cerebral infarction due to thrombosis of cerebral arteries		Patient included	Patient included, date of the event is the minimum between "epistart" and "admidate"	Patient included if the first event was after the UK Biobank recruitment date (Field 53), date of the event is the minimum between "epistart" and "admidate"
I634		I63.4 Cerebral infarction due to embolism of cerebral arteries		Patient included	Patient included, date of the event is the minimum between "epistart" and "admidate"	Patient included if the first event was after the UK Biobank recruitment date (Field 53), date of the event is the minimum between "epistart" and "admidate"
I635		I63.5 Cerebral infarction due to unspecified occlusion or stenosis of cerebral arteries		Patient included	Patient included, date of the event is the minimum between "epistart" and "admidate"	Patient included if the first event was after the UK Biobank recruitment date (Field 53), date of the event is the minimum between "epistart" and "admidate"
I636		I63.6 Cerebral infarction due to cerebral venous thrombosis, nonpyogenic		Patient included	Patient included, date of the event is the minimum between "epistart" and "admidate"	Patient included if the first event was after the UK Biobank recruitment date (Field 53), date of the event is the minimum between "epistart" and "admidate"
I638		I63.8 Other cerebral infarction		Patient included	Patient included, date of the event is the minimum between "epistart" and "admidate"	Patient included if the first event was after the UK Biobank recruitment date (Field 53), date of the event is the minimum between "epistart" and "admidate"
I639		I63.9 Cerebral infarction, unspecified		Patient included	Patient included, date of the event is the minimum between "epistart" and "admidate"	Patient included if the first event was after the UK Biobank recruitment date (Field 53), date of the event is the minimum between "epistart" and "admidate"
Source data: Linkage to GP						

Read2 code	Code	Description	Extra information	As binary utcome	As survival outcome (since birth date)	As survival outcome (since enrolment date)
G63y0		Cerebral infarct due to thrombosis of precerebral arteries		Patient included	Patient included, date of the event is "event_dt"	Patient included if the first event was after the UK Biobank recruitment date (Field 53), date of the event is "event_dt"
G63y1		Cerebral infarction due to embolism of precerebral arteries		Patient included	Patient included, date of the event is "event_dt"	Patient included if the first event was after the UK Biobank recruitment date (Field 53), date of the event is "event_dt"
G6400		Cerebral infarction due to thrombosis of cerebral arteries		Patient included	Patient included, date of the event is "event_dt"	Patient included if the first event was after the UK Biobank recruitment date (Field 53), date of the event is "event_dt"
G6410		Cerebral infarction due to embolism of cerebral arteries		Patient included	Patient included, date of the event is "event_dt"	Patient included if the first event was after the UK Biobank recruitment date (Field 53), date of the event is "event_dt"
G64z.		Cerebral infarction NOS		Patient included	Patient included, date of the event is "event_dt"	Patient included if the first event was after the UK Biobank recruitment date (Field 53), date of the event is "event_dt"
G64z0		Brainstem infarction		Patient included	Patient included, date of the event is "event_dt"	Patient included if the first event was after the UK Biobank recruitment date (Field 53), date of the event is "event_dt"
G64z2		Left sided cerebral infarction		Patient included	Patient included, date of the event is "event_dt"	Patient included if the first event was after the UK Biobank recruitment date (Field 53), date of the event is "event_dt"
G64z3		Right sided cerebral infarction		Patient included	Patient included, date of the event is "event_dt"	Patient included if the first event was after the UK Biobank recruitment date (Field 53), date of the event is "event_dt"
G64z4		Infarction of basal ganglia		Patient included	Patient included, date of the event is "event_dt"	Patient included if the first event was after the UK Biobank recruitment date (Field 53), date of the event is "event_dt"
G6760		Cerebral infarction due to cerebral venous thrombosis, nonpyogenic		Patient included	Patient included, date of the event is "event_dt"	Patient included if the first event was after the UK Biobank recruitment date (Field 53), date of the event is "event_dt"
G6W..		Cerebral infarction due to unspecified occlusion or stenosis of precerebral arteries		Patient included	Patient included, date of the event is "event_dt"	Patient included if the first event was after the UK Biobank recruitment date (Field 53), date of the event is "event_dt"
G6X..		Cerebral infarction due to unspecified occlusion or stenosis of cerebral arteries		Patient included	Patient included, date of the event is "event_dt"	Patient included if the first event was after the UK Biobank recruitment date (Field 53), date of the event is "event_dt"
Gyu63		[X]Cerebral infarction due to unspecified occlusion or stenosis of cerebral arteries		Patient included	Patient included, date of the event is "event_dt"	Patient included if the first event was after the UK Biobank recruitment date (Field 53), date of the event is "event_dt"
Gyu64		[X]Other cerebral infarction		Patient included	Patient included, date of the event is "event_dt"	Patient included if the first event was after the UK Biobank recruitment date (Field 53), date of the event is "event_dt"
Gyu6G		[X]Cerebral infarction due to unspecified occlusion or stenosis of precerebral arteries		Patient included	Patient included, date of the event is "event_dt"	Patient included if the first event was after the UK Biobank recruitment date (Field 53), date of the event is "event_dt"
**Hypertension outcome**						
Source data: UK Biobank						
**Field**	**Code**	**Description**	**Extra information**	**As binary utcome**	**As survival outcome (since birth date)**	**As survival outcome (since enrolment date)**
20002	1065	Non-cancer illness code, self-reported - Hypertension		Patient included	Patient included, years at the event is Field 20009 (Interpolated year/age of the event)	Patient excluded
Source data: Linkage to HES						
**ICD10**	**Code**	**Description**	**Extra information**	**As binary utcome**	**As survival outcome (since birth date)**	**As survival outcome (since enrolment date)**
I10		Essential (primary) hypertension		Patient included	Patient included, date of the event is the minimum between "epistart" and "admidate"	Patient included if the first event was after the UK Biobank recruitment date (Field 53), date of the event is the minimum between "epistart" and "admidate"
I11		Hypertensive heart disease		Patient included	Patient included, date of the event is the minimum between "epistart" and "admidate"	Patient included if the first event was after the UK Biobank recruitment date (Field 53), date of the event is the minimum between "epistart" and "admidate"
I110		Hypertensive heart disease with (congestive) heart failure		Patient included	Patient included, date of the event is the minimum between "epistart" and "admidate"	Patient included if the first event was after the UK Biobank recruitment date (Field 53), date of the event is the minimum between "epistart" and "admidate"
I119		Hypertensive heart disease without (congestive) heart failure		Patient included	Patient included, date of the event is the minimum between "epistart" and "admidate"	Patient included if the first event was after the UK Biobank recruitment date (Field 53), date of the event is the minimum between "epistart" and "admidate"
I12		Hypertensive renal disease		Patient included	Patient included, date of the event is the minimum between "epistart" and "admidate"	Patient included if the first event was after the UK Biobank recruitment date (Field 53), date of the event is the minimum between "epistart" and "admidate"
I120		Hypertensive renal disease with renal failure		Patient included	Patient included, date of the event is the minimum between "epistart" and "admidate"	Patient included if the first event was after the UK Biobank recruitment date (Field 53), date of the event is the minimum between "epistart" and "admidate"
I129		Hypertensive renal disease without renal failure		Patient included	Patient included, date of the event is the minimum between "epistart" and "admidate"	Patient included if the first event was after the UK Biobank recruitment date (Field 53), date of the event is the minimum between "epistart" and "admidate"

I13		Hypertensive heart and renal disease		Patient included	Patient included, date of the event is the minimum between "epistart" and "admidate"	Patient included if the first event was after the UK Biobank recruitment date (Field 53), date of the event is the minimum between "epistart" and "admidate"
I130		Hypertensive heart and renal disease with (congestive) heart failure		Patient included	Patient included, date of the event is the minimum between "epistart" and "admidate"	Patient included if the first event was after the UK Biobank recruitment date (Field 53), date of the event is the minimum between "epistart" and "admidate"
I131		Hypertensive heart and renal disease with renal failure		Patient included	Patient included, date of the event is the minimum between "epistart" and "admidate"	Patient included if the first event was after the UK Biobank recruitment date (Field 53), date of the event is the minimum between "epistart" and "admidate"
I132		Hypertensive heart and renal disease with both (congestive) heart failure and renal failure		Patient included	Patient included, date of the event is the minimum between "epistart" and "admidate"	Patient included if the first event was after the UK Biobank recruitment date (Field 53), date of the event is the minimum between "epistart" and "admidate"
I139		Hypertensive heart and renal disease, unspecified		Patient included	Patient included, date of the event is the minimum between "epistart" and "admidate"	Patient included if the first event was after the UK Biobank recruitment date (Field 53), date of the event is the minimum between "epistart" and "admidate"
I15		Secondary hypertension		Patient included	Patient included, date of the event is the minimum between "epistart" and "admidate"	Patient included if the first event was after the UK Biobank recruitment date (Field 53), date of the event is the minimum between "epistart" and "admidate"
I150		Renovascular hypertension		Patient included	Patient included, date of the event is the minimum between "epistart" and "admidate"	Patient included if the first event was after the UK Biobank recruitment date (Field 53), date of the event is the minimum between "epistart" and "admidate"
I151		Hypertension secondary to other renal disorders		Patient included	Patient included, date of the event is the minimum between "epistart" and "admidate"	Patient included if the first event was after the UK Biobank recruitment date (Field 53), date of the event is the minimum between "epistart" and "admidate"
I152		Hypertension secondary to endocrine disorders		Patient included	Patient included, date of the event is the minimum between "epistart" and "admidate"	Patient included if the first event was after the UK Biobank recruitment date (Field 53), date of the event is the minimum between "epistart" and "admidate"
I158		Other secondary hypertension		Patient included	Patient included, date of the event is the minimum between "epistart" and "admidate"	Patient included if the first event was after the UK Biobank recruitment date (Field 53), date of the event is the minimum between "epistart" and "admidate"
I159		Secondary hypertension, unspecified		Patient included	Patient included, date of the event is the minimum between "epistart" and "admidate"	Patient included if the first event was after the UK Biobank recruitment date (Field 53), date of the event is the minimum between "epistart" and "admidate"
Source data: Linkage to GP						
**Read2 code**	**Code**	**Description**	**Extra information**	**As binary utcome**	**As survival outcome (since birth date)**	**As survival outcome (since enrolment date)**
G21..		Hypertensive heart disease		Patient included	Patient included, date of the event is event_dt	Patient included if the first event was after the UK Biobank recruitment date (Field 53), date of the event is "event_dt"
G210.		Malignant hypertensive heart disease		Patient included	Patient included, date of the event is event_dt	Patient included if the first event was after the UK Biobank recruitment date (Field 53), date of the event is "event_dt"
G2100		Malignant hypertensive heart disease without congestive cardiac failure		Patient included	Patient included, date of the event is event_dt	Patient included if the first event was after the UK Biobank recruitment date (Field 53), date of the event is "event_dt"
G2101		Malignant hypertensive heart disease with congestive cardiac failure		Patient included	Patient included, date of the event is event_dt	Patient included if the first event was after the UK Biobank recruitment date (Field 53), date of the event is "event_dt"
G210z		Malignant hypertensive heart disease NOS		Patient included	Patient included, date of the event is event_dt	Patient included if the first event was after the UK Biobank recruitment date (Field 53), date of the event is "event_dt"
G211.		Benign hypertensive heart disease		Patient included	Patient included, date of the event is event_dt	Patient included if the first event was after the UK Biobank recruitment date (Field 53), date of the event is "event_dt"
G2110		Benign hypertensive heart disease without congestive cardiac failure		Patient included	Patient included, date of the event is event_dt	Patient included if the first event was after the UK Biobank recruitment date (Field 53), date of the event is "event_dt"
G2111		Benign hypertensive heart disease with congestive cardiac failure		Patient included	Patient included, date of the event is event_dt	Patient included if the first event was after the UK Biobank recruitment date (Field 53), date of the event is "event_dt"
G211z		Benign hypertensive heart disease NOS		Patient included	Patient included, date of the event is event_dt	Patient included if the first event was after the UK Biobank recruitment date (Field 53), date of the event is "event_dt"
G21z.		Hypertensive heart disease NOS		Patient included	Patient included, date of the event is event_dt	Patient included if the first event was after the UK Biobank recruitment date (Field 53), date of the event is "event_dt"
G21z0		Hypertensive heart disease NOS without congestive cardic failure		Patient included	Patient included, date of the event is event_dt	Patient included if the first event was after the UK Biobank recruitment date (Field 53), date of the event is "event_dt"
G21z1		Hypertensive heart disease NOS with congestive cardiac failure		Patient included	Patient included, date of the event is event_dt	Patient included if the first event was after the UK Biobank recruitment date (Field 53), date of the event is "event_dt"
G21zz		Hypertensive heart disease NOS		Patient included	Patient included, date of the event is event_dt	Patient included if the first event was after the UK Biobank recruitment date (Field 53), date of the event is "event_dt"

G22..		Hypertensive renal disease		Patient included	Patient included, date of the event is event_dt	Patient included if the first event was after the UK Biobank recruitment date (Field 53), date of the event is "event_dt"
G220.		Malignant hypertensive renal disease		Patient included	Patient included, date of the event is event_dt	Patient included if the first event was after the UK Biobank recruitment date (Field 53), date of the event is "event_dt"
G221.		Benign hypertensive renal disease		Patient included	Patient included, date of the event is event_dt	Patient included if the first event was after the UK Biobank recruitment date (Field 53), date of the event is "event_dt"
G222.		Hypertensive renal disease with renal failure		Patient included	Patient included, date of the event is event_dt	Patient included if the first event was after the UK Biobank recruitment date (Field 53), date of the event is "event_dt"
G22z.		Hypertensive renal disease NOS		Patient included	Patient included, date of the event is event_dt	Patient included if the first event was after the UK Biobank recruitment date (Field 53), date of the event is "event_dt"
G23..		Hypertensive heart and renal disease		Patient included	Patient included, date of the event is event_dt	Patient included if the first event was after the UK Biobank recruitment date (Field 53), date of the event is "event_dt"
G230.		Malignant hypertensive heart and renal disease		Patient included	Patient included, date of the event is event_dt	Patient included if the first event was after the UK Biobank recruitment date (Field 53), date of the event is "event_dt"
G231.		Benign hypertensive heart and renal disease		Patient included	Patient included, date of the event is event_dt	Patient included if the first event was after the UK Biobank recruitment date (Field 53), date of the event is "event_dt"
G232.		Hypertensive heart and renal disease with (congestive) heart failure		Patient included	Patient included, date of the event is event_dt	Patient included if the first event was after the UK Biobank recruitment date (Field 53), date of the event is "event_dt"
G233.		Hypertensive heart and renal disease with renal failure		Patient included	Patient included, date of the event is event_dt	Patient included if the first event was after the UK Biobank recruitment date (Field 53), date of the event is "event_dt"
G234.		Hypertensive heart and renal disease with both (congestive) heart failure and renal failure		Patient included	Patient included, date of the event is event_dt	Patient included if the first event was after the UK Biobank recruitment date (Field 53), date of the event is "event_dt"
G23z.		Hypertensive heart and renal disease NOS		Patient included	Patient included, date of the event is event_dt	Patient included if the first event was after the UK Biobank recruitment date (Field 53), date of the event is "event_dt"
G24..		Secondary hypertension		Patient included	Patient included, date of the event is event_dt	Patient included if the first event was after the UK Biobank recruitment date (Field 53), date of the event is "event_dt"
G240.		Secondary malignant hypertension		Patient included	Patient included, date of the event is event_dt	Patient included if the first event was after the UK Biobank recruitment date (Field 53), date of the event is "event_dt"
G2400		Secondary malignant renovascular hypertension		Patient included	Patient included, date of the event is event_dt	Patient included if the first event was after the UK Biobank recruitment date (Field 53), date of the event is "event_dt"
G240z		Secondary malignant hypertension NOS		Patient included	Patient included, date of the event is event_dt	Patient included if the first event was after the UK Biobank recruitment date (Field 53), date of the event is "event_dt"
G241.		Secondary benign hypertension		Patient included	Patient included, date of the event is event_dt	Patient included if the first event was after the UK Biobank recruitment date (Field 53), date of the event is "event_dt"
G2410		Secondary benign renovascular hypertension		Patient included	Patient included, date of the event is event_dt	Patient included if the first event was after the UK Biobank recruitment date (Field 53), date of the event is "event_dt"
G241z		Secondary benign hypertension NOS		Patient included	Patient included, date of the event is event_dt	Patient included if the first event was after the UK Biobank recruitment date (Field 53), date of the event is "event_dt"
G244.		Hypertension secondary to endocrine disorders		Patient included	Patient included, date of the event is event_dt	Patient included if the first event was after the UK Biobank recruitment date (Field 53), date of the event is "event_dt"
G24z.		Secondary hypertension NOS		Patient included	Patient included, date of the event is event_dt	Patient included if the first event was after the UK Biobank recruitment date (Field 53), date of the event is "event_dt"
G24z0		Secondary renovascular hypertension NOS		Patient included	Patient included, date of the event is event_dt	Patient included if the first event was after the UK Biobank recruitment date (Field 53), date of the event is "event_dt"
G24z1		Hypertension secondary to drug		Patient included	Patient included, date of the event is event_dt	Patient included if the first event was after the UK Biobank recruitment date (Field 53), date of the event is "event_dt"
G24zz		Secondary hypertension NOS		Patient included	Patient included, date of the event is event_dt	Patient included if the first event was after the UK Biobank recruitment date (Field 53), date of the event is "event_dt"
Gyu20				Patient included	Patient included, date of the event is event_dt	Patient included if the first event was after the UK Biobank recruitment date (Field 53), date of the event is "event_dt"
Gyu21		[X]Hypertension secondary to other renal disorders		Patient included	Patient included, date of the event is event_dt	Patient included if the first event was after the UK Biobank recruitment date (Field 53), date of the event is "event_dt"
K138.		Vascular disorders of kidney		Patient included	Patient included, date of the event is event_dt	Patient included if the first event was after the UK Biobank recruitment date (Field 53), date of the event is "event_dt"
K138.		Vascular disorders of kidney		Patient included	Patient included, date of the event is event_dt	Patient included if the first event was after the UK Biobank recruitment date (Field 53), date of the event is "event_dt"
K1386		Renal vascular disease		Patient included	Patient included, date of the event is event_dt	Patient included if the first event was after the UK Biobank recruitment date (Field 53), date of the event is "event_dt"
**Type 2 diabetes outcome**						
Source data: UK Biobank						

Field	Code	Description	Extra information	As binary utcome	As survival outcome (since birth date)	As survival outcome (since enrolment date)
2443		Diabetes diagnosed by a doctor		Patient included	Patient included, years at the event is provided by Field 2976 (Age diabetes diagnosed)	Patient excluded
20002	1223	Non-cancer illness code, self-reported - Type 2 diabetes		Patient included	Patient included, years at the event is provided by field 20009 (Interpolated year/age of the event)	Patient excluded
20003	1140874646	Glipizide		Patient included	Patient excluded	Patient excluded
20003	1140874664	Tolazamide		Patient included	Patient excluded	Patient excluded
20003	1140874674	Tolbutamide		Patient included	Patient excluded	Patient excluded
20003	1140874686	Glucophage		Patient included	Patient excluded	Patient excluded
20003	1140874706	Chlorpropamide)		Patient included	Patient excluded	Patient excluded
20003	1140874718	Glibenclamide		Patient included	Patient excluded	Patient excluded
20003	1140874744	Gliclazide		Patient included	Patient excluded	Patient excluded
20003	1141189090	Rosiglitazone/Metformin		Patient included	Patient excluded	Patient excluded
20003	1141171646	Pioglitazone		Patient included	Patient excluded	Patient excluded
20003	1140868902	Acarbose		Patient included	Patient excluded	Patient excluded
20003	1141168660	Repaglinide		Patient included	Patient excluded	Patient excluded
20003	1141152590	Glimepiride		Patient included	Patient excluded	Patient excluded
20003	1140874650	Glibenese		Patient included	Patient excluded	Patient excluded
20003	1140874652	Minodiab		Patient included	Patient excluded	Patient excluded
20003	1140874658	Gliquidone		Patient included	Patient excluded	Patient excluded
20003	1140874660	Glurenorm		Patient included	Patient excluded	Patient excluded
20003	1140874666	Tolanase		Patient included	Patient excluded	Patient excluded
20003	1140874678	Glyconon		Patient included	Patient excluded	Patient excluded
20003	1140874680	Rastinon		Patient included	Patient excluded	Patient excluded
20003	1140874690	Orabet		Patient included	Patient excluded	Patient excluded
20003	1140874712	Diabinese		Patient included	Patient excluded	Patient excluded
20003	1140874716	Glymese		Patient included	Patient excluded	Patient excluded
20003	1140874724	Daonil		Patient included	Patient excluded	Patient excluded
20003	1140874726	Semi-Daonil		Patient included	Patient excluded	Patient excluded
20003	1140874728	Euglucon		Patient included	Patient excluded	Patient excluded
20003	1140874732	Malix		Patient included	Patient excluded	Patient excluded
20003	1140874736	Diabetamide		Patient included	Patient excluded	Patient excluded
20003	1140874740	Calabren		Patient included	Patient excluded	Patient excluded
20003	1140874746	Diamicron		Patient included	Patient excluded	Patient excluded
20003	1141171652	Actos		Patient included	Patient excluded	Patient excluded
20003	1140868908	Glucobay		Patient included	Patient excluded	Patient excluded
20003	1141168668	Novonorm		Patient included	Patient excluded	Patient excluded
20003	1141157284	Glipizide product		Patient included	Patient excluded	Patient excluded
30750	HbA1c	HbA1c greater than 48 mmol/mol is diagnosed as diabetes		Patient included	Patient excluded	Patient excluded
Source data: Linkage to HES						
**ICD10**	**Code**	**Description**	**Extra information**	**As binary utcome**	**As survival outcome (since birth date)**	**As survival outcome (since enrolment date)**
E11		Non-insulin-dependent diabetes mellitus		Patients included	Patient included, date of the event is the minimum between "epistart" and "admidate"	Patient included if the first event was after the UK Biobank recruitment date (Field 53), date of the event is the minimum between "epistart" and "admidate"
E110		With coma		Patients included	Patient included, date of the event is the minimum between "epistart" and "admidate"	Patient included if the first event was after the UK Biobank recruitment date (Field 53), date of the event is the minimum between "epistart" and "admidate"
E111		With ketoacidosis		Patients included	Patient included, date of the event is the minimum between "epistart" and "admidate"	Patient included if the first event was after the UK Biobank recruitment date (Field 53), date of the event is the minimum between "epistart" and "admidate"
E112		With renal complications		Patients included	Patient included, date of the event is the minimum between "epistart" and "admidate"	Patient included if the first event was after the UK Biobank recruitment date (Field 53), date of the event is the minimum between "epistart" and "admidate"
E113		With ophthalmic complications		Patients included	Patient included, date of the event is the minimum between "epistart" and "admidate"	Patient included if the first event was after the UK Biobank recruitment date (Field 53), date of the event is the minimum between "epistart" and "admidate"

E114		With neurological complications		Patients included	Patient included, date of the event is the minimum between "epistart" and "admidate"	Patient included if the first event was after the UK Biobank recruitment date (Field 53), date of the event is the minimum between "epistart" and "admidate"
E115		With peripheral circulatory complications		Patients included	Patient included, date of the event is the minimum between "epistart" and "admidate"	Patient included if the first event was after the UK Biobank recruitment date (Field 53), date of the event is the minimum between "epistart" and "admidate"
E116		With other specified complications		Patients included	Patient included, date of the event is the minimum between "epistart" and "admidate"	Patient included if the first event was after the UK Biobank recruitment date (Field 53), date of the event is the minimum between "epistart" and "admidate"
E117		With multiple complications		Patients included	Patient included, date of the event is the minimum between "epistart" and "admidate"	Patient included if the first event was after the UK Biobank recruitment date (Field 53), date of the event is the minimum between "epistart" and "admidate"
E118		With unspecified complications		Patients included	Patient included, date of the event is the minimum between "epistart" and "admidate"	Patient included if the first event was after the UK Biobank recruitment date (Field 53), date of the event is the minimum between "epistart" and "admidate"
E119		Without complications		Patients included	Patient included, date of the event is the minimum between "epistart" and "admidate"	Patient included if the first event was after the UK Biobank recruitment date (Field 53), date of the event is the minimum between "epistart" and "admidate"
Source data: Linkage to GP						
**Read2 code**	**Code**	**Description**	**Extra information**	**As binary utcome**	**As survival outcome (since birth date)**	**As survival outcome (since enrolment date)**
C109.	E110	Type 2 diabetes mellitus		Patients included	Patient included, date of the event is "event_dt"	Patient included if the first event was after the UK Biobank recruitment date (Field 53), date of the event is "event_dt"
C1001	E119	Diabetes mellitus, adult onset, with no mention of complication		Patients included	Patient included, date of the event is "event_dt"	Patient included if the first event was after the UK Biobank recruitment date (Field 53), date of the event is "event_dt"
C1011	E111	Diabetes mellitus, adult onset, with ketoacidosis		Patients included	Patient included, date of the event is "event_dt"	Patient included if the first event was after the UK Biobank recruitment date (Field 53), date of the event is "event_dt"
C1021	E110	Diabetes mellitus, adult onset, with hyperosmolar coma		Patients included	Patient included, date of the event is "event_dt"	Patient included if the first event was after the UK Biobank recruitment date (Field 53), date of the event is "event_dt"
C1031	E110	Diabetes mellitus, adult onset, with ketoacidotic coma		Patients included	Patient included, date of the event is "event_dt"	Patient included if the first event was after the UK Biobank recruitment date (Field 53), date of the event is "event_dt"
C1071	E115	Diabetes mellitus, adult onset, with peripheral circulatory disorder		Patients included	Patient included, date of the event is "event_dt"	Patient included if the first event was after the UK Biobank recruitment date (Field 53), date of the event is "event_dt"
C1072	E115	Diabetes mellitus, adult with gangrene		Patients included	Patient included, date of the event is "event_dt"	Patient included if the first event was after the UK Biobank recruitment date (Field 53), date of the event is "event_dt"
C1074	E115	NIDDM with peripheral circulatory disorder		Patients included	Patient included, date of the event is "event_dt"	Patient included if the first event was after the UK Biobank recruitment date (Field 53), date of the event is "event_dt"
C1091	E113	Non-insulin-dependent diabetes mellitus with ophthalmic complications		Patients included	Patient included, date of the event is "event_dt"	Patient included if the first event was after the UK Biobank recruitment date (Field 53), date of the event is "event_dt"
C1093	E117	Non-insulin-dependent diabetes mellitus with multiple complications		Patients included	Patient included, date of the event is "event_dt"	Patient included if the first event was after the UK Biobank recruitment date (Field 53), date of the event is "event_dt"
C1094	E115	Non-insulin dependent diabetes mellitus with ulcer		Patients included	Patient included, date of the event is "event_dt"	Patient included if the first event was after the UK Biobank recruitment date (Field 53), date of the event is "event_dt"
C1094	E115	Non-insulin dependent diabetes mellitus with ulcer		Patients included	Patient included, date of the event is "event_dt"	Patient included if the first event was after the UK Biobank recruitment date (Field 53), date of the event is "event_dt"
C1095	E115	Non-insulin dependent diabetes mellitus with gangrene		Patients included	Patient included, date of the event is "event_dt"	Patient included if the first event was after the UK Biobank recruitment date (Field 53), date of the event is "event_dt"
C1095	E115	Non-insulin dependent diabetes mellitus with gangrene		Patients included	Patient included, date of the event is "event_dt"	Patient included if the first event was after the UK Biobank recruitment date (Field 53), date of the event is "event_dt"
C1097	E119	Non-insulin dependent diabetes mellitus - poor control		Patients included	Patient included, date of the event is "event_dt"	Patient included if the first event was after the UK Biobank recruitment date (Field 53), date of the event is "event_dt"
C1097	E119	Non-insulin dependent diabetes mellitus - poor control		Patients included	Patient included, date of the event is "event_dt"	Patient included if the first event was after the UK Biobank recruitment date (Field 53), date of the event is "event_dt"
C1099	E119	Non-insulin-dependent diabetes mellitus without complication		Patients included	Patient included, date of the event is "event_dt"	Patient included if the first event was after the UK Biobank recruitment date (Field 53), date of the event is "event_dt"
C109D	E110	Non-insulin dependent diabetes mellitus with hypoglycaemic coma		Patients included	Patient included, date of the event is "event_dt"	Patient included if the first event was after the UK Biobank recruitment date (Field 53), date of the event is "event_dt"
C109J	E119	Insulin treated Type 2 diabetes mellitus		Patients included	Patient included, date of the event is "event_dt"	Patient included if the first event was after the UK Biobank recruitment date (Field 53), date of the event is "event_dt"
C109K	E119	Hyperosmolar non-ketotic state in type 2 diabetes mellitus		Patients included	Patient included, date of the event is "event_dt"	Patient included if the first event was after the UK Biobank recruitment date (Field 53), date of the event is "event_dt"

C10F3	E117	Type 2 diabetes mellitus with multiple complications		Patients included	Patient included, date of the event is "event_dt"	Patient included if the first event was after the UK Biobank recruitment date (Field 53), date of the event is "event_dt"
C10F4	E115	Type 2 diabetes mellitus with ulcer		Patients included	Patient included, date of the event is "event_dt"	Patient included if the first event was after the UK Biobank recruitment date (Field 53), date of the event is "event_dt"
C10F5	E115	Type 2 diabetes mellitus with gangrene		Patients included	Patient included, date of the event is "event_dt"	Patient included if the first event was after the UK Biobank recruitment date (Field 53), date of the event is "event_dt"
C10F7	E119	Type 2 diabetes mellitus - poor control		Patients included	Patient included, date of the event is "event_dt"	Patient included if the first event was after the UK Biobank recruitment date (Field 53), date of the event is "event_dt"
C10F8	E116	Reaven's syndrome		Patients included	Patient included, date of the event is "event_dt"	Patient included if the first event was after the UK Biobank recruitment date (Field 53), date of the event is "event_dt"
C10F9	E119	Type 2 diabetes mellitus without complication		Patients included	Patient included, date of the event is "event_dt"	Patient included if the first event was after the UK Biobank recruitment date (Field 53), date of the event is "event_dt"
C10FD	E110	Type 2 diabetes mellitus with hypoglycaemic coma		Patients included	Patient included, date of the event is "event_dt"	Patient included if the first event was after the UK Biobank recruitment date (Field 53), date of the event is "event_dt"
C10FJ	E119	Insulin treated Type 2 diabetes mellitus		Patients included	Patient included, date of the event is "event_dt"	Patient included if the first event was after the UK Biobank recruitment date (Field 53), date of the event is "event_dt"
C10FK	E119	Hyperosmolar non-ketotic state in type 2 diabetes mellitus		Patients included	Patient included, date of the event is "event_dt"	Patient included if the first event was after the UK Biobank recruitment date (Field 53), date of the event is "event_dt"
C10FN	E111	Type 2 diabetes mellitus with ketoacidosis		Patients included	Patient included, date of the event is "event_dt"	Patient included if the first event was after the UK Biobank recruitment date (Field 53), date of the event is "event_dt"
C10FP	E110	Type 2 diabetes mellitus with ketoacidotic coma		Patients included	Patient included, date of the event is "event_dt"	Patient included if the first event was after the UK Biobank recruitment date (Field 53), date of the event is "event_dt"
C10FS	E119	Maternally inherited diabetes mellitus		Patients included	Patient included, date of the event is "event_dt"	Patient included if the first event was after the UK Biobank recruitment date (Field 53), date of the event is "event_dt"
C10P1	E119	Type II diabetes mellitus in remission		Patients included	Patient included, date of the event is "event_dt"	Patient included if the first event was after the UK Biobank recruitment date (Field 53), date of the event is "event_dt"
C10Q.	E119	Maturity onset diabetes of the young type 5		Patients included	Patient included, date of the event is "event_dt"	Patient included if the first event was after the UK Biobank recruitment date (Field 53), date of the event is "event_dt"
C10y1	E116	Diabetes mellitus, adult onset, with other specified manifestation		Patients included	Patient included, date of the event is "event_dt"	Patient included if the first event was after the UK Biobank recruitment date (Field 53), date of the event is "event_dt"
C10z1	E118	Diabetes mellitus, adult onset, with unspecified complication		Patients included	Patient included, date of the event is "event_dt"	Patient included if the first event was after the UK Biobank recruitment date (Field 53), date of the event is "event_dt"
f36..	Drugs	Glipizide		Patients included	Patient included, date of the event is "event_dt"	Patient included if the first event was after the UK Biobank recruitment date (Field 53), date of the event is "event_dt"
f361.	Drugs	Glipizide	5mg tablets	Patients included	Patient included, date of the event is "event_dt"	Patient included if the first event was after the UK Biobank recruitment date (Field 53), date of the event is "event_dt"
f36y.	Drugs	Glipizide	*5mg tablets	Patients included	Patient included, date of the event is "event_dt"	Patient included if the first event was after the UK Biobank recruitment date (Field 53), date of the event is "event_dt"
f36z.	Drugs	Glipizide	2.5mg tablets	Patients included	Patient included, date of the event is "event_dt"	Patient included if the first event was after the UK Biobank recruitment date (Field 53), date of the event is "event_dt"
f39..	Drugs	Tolazamide		Patients included	Patient included, date of the event is "event_dt"	Patient included if the first event was after the UK Biobank recruitment date (Field 53), date of the event is "event_dt"
f39y.	Drugs	Tolazamide	100mg tablets	Patients included	Patient included, date of the event is "event_dt"	Patient included if the first event was after the UK Biobank recruitment date (Field 53), date of the event is "event_dt"
f39z.	Drugs	Tolazamide	250mg tablets	Patients included	Patient included, date of the event is "event_dt"	Patient included if the first event was after the UK Biobank recruitment date (Field 53), date of the event is "event_dt"
f3a..	Drugs	Tolbutamide		Patients included	Patient included, date of the event is "event_dt"	Patient included if the first event was after the UK Biobank recruitment date (Field 53), date of the event is "event_dt"
f3a1.	Drugs	Tolbutamide	500mg tablets	Patients included	Patient included, date of the event is "event_dt"	Patient included if the first event was after the UK Biobank recruitment date (Field 53), date of the event is "event_dt"
f411.	Drugs	Glucophage	500mg tablets	Patients included	Patient included, date of the event is "event_dt"	Patient included if the first event was after the UK Biobank recruitment date (Field 53), date of the event is "event_dt"
f412.	Drugs	Glucophage	850mg tablets	Patients included	Patient included, date of the event is "event_dt"	Patient included if the first event was after the UK Biobank recruitment date (Field 53), date of the event is "event_dt"
f417.	Drugs	Glucophage	SR 500mg m/r tablets	Patients included	Patient included, date of the event is "event_dt"	Patient included if the first event was after the UK Biobank recruitment date (Field 53), date of the event is "event_dt"
f419.	Drugs	Glucophage	SR 750mg m/r tablets	Patients included	Patient included, date of the event is "event_dt"	Patient included if the first event was after the UK Biobank recruitment date (Field 53), date of the event is "event_dt"
f41A.	Drugs	Glucophage	SR 1000mg m/r tablets	Patients included	Patient included, date of the event is "event_dt"	Patient included if the first event was after the UK Biobank recruitment date (Field 53), date of the event is "event_dt"

f41C.	Drugs	Glucophage	500mg/sachet oral powder	Patients included	Patient included, date of the event is "event_dt"	Patient included if the first event was after the UK Biobank recruitment date (Field 53), date of the event is "event_dt"
f41D.	Drugs	Glucophage	1000mg/sachet oral powder	Patients included	Patient included, date of the event is "event_dt"	Patient included if the first event was after the UK Biobank recruitment date (Field 53), date of the event is "event_dt"
f32..	Drugs	Chlorpropamide		Patients included	Patient included, date of the event is "event_dt"	Patient included if the first event was after the UK Biobank recruitment date (Field 53), date of the event is "event_dt"
f321.	Drugs	Chlorpropamide	100ma tablets	Patients included	Patient included, date of the event is "event_dt"	Patient included if the first event was after the UK Biobank recruitment date (Field 53), date of the event is "event_dt"
f322.	Drugs	Chlorpropamide	250mg tablets	Patients included	Patient included, date of the event is "event_dt"	Patient included if the first event was after the UK Biobank recruitment date (Field 53), date of the event is "event_dt"
f33..	Drugs	Glibenclamide		Patients included	Patient included, date of the event is "event_dt"	Patient included if the first event was after the UK Biobank recruitment date (Field 53), date of the event is "event_dt"
f331.	Drugs	Glibenclamide	2.5mg tablets	Patients included	Patient included, date of the event is "event_dt"	Patient included if the first event was after the UK Biobank recruitment date (Field 53), date of the event is "event_dt"
f332.	Drugs	Glibenclamide	5mg tablets	Patients included	Patient included, date of the event is "event_dt"	Patient included if the first event was after the UK Biobank recruitment date (Field 53), date of the event is "event_dt"
f35..	Drugs	Gliclazide		Patients included	Patient included, date of the event is "event_dt"	Patient included if the first event was after the UK Biobank recruitment date (Field 53), date of the event is "event_dt"
f35w.	Drugs	Gliclazide	60mg m/r tablets	Patients included	Patient included, date of the event is "event_dt"	Patient included if the first event was after the UK Biobank recruitment date (Field 53), date of the event is "event_dt"
f35x.	Drugs	Gliclazide	40mg tablets	Patients included	Patient included, date of the event is "event_dt"	Patient included if the first event was after the UK Biobank recruitment date (Field 53), date of the event is "event_dt"
f35y.	Drugs	Gliclazide	30mg m/r tablets	Patients included	Patient included, date of the event is "event_dt"	Patient included if the first event was after the UK Biobank recruitment date (Field 53), date of the event is "event_dt"
f35z.	Drugs	Gliclazide	80mg tablets	Patients included	Patient included, date of the event is "event_dt"	Patient included if the first event was after the UK Biobank recruitment date (Field 53), date of the event is "event_dt"
ft7..	Drugs	Metformin	Metformin + Pioglitazone	Patients included	Patient included, date of the event is "event_dt"	Patient included if the first event was after the UK Biobank recruitment date (Field 53), date of the event is "event_dt"
ft7z.	Drugs	Metformin	Metformin 850mg/Pioglitazone 15mg tablets	Patients included	Patient included, date of the event is "event_dt"	Patient included if the first event was after the UK Biobank recruitment date (Field 53), date of the event is "event_dt"
ftb..	Drugs	Metformin	Metformin + Vildagliptin	Patients included	Patient included, date of the event is "event_dt"	Patient included if the first event was after the UK Biobank recruitment date (Field 53), date of the event is "event_dt"
ftby.	Drugs	Metformin	Vildagliptin/Metformin 50mg/1000mg tablets	Patients included	Patient included, date of the event is "event_dt"	Patient included if the first event was after the UK Biobank recruitment date (Field 53), date of the event is "event_dt"
ftbz.	Drugs	Metformin	Vildagliptin/Metformin 50mg/850mg tablets	Patients included	Patient included, date of the event is "event_dt"	Patient included if the first event was after the UK Biobank recruitment date (Field 53), date of the event is "event_dt"
fte..	Drugs	Metformin	Metformin + Sitagliptin	Patients included	Patient included, date of the event is "event_dt"	Patient included if the first event was after the UK Biobank recruitment date (Field 53), date of the event is "event_dt"
ftez.	Drugs	Metformin	Sitagliptin/Metformin Hydrochloride 50mg/1000mg tablets	Patients included	Patient included, date of the event is "event_dt"	Patient included if the first event was after the UK Biobank recruitment date (Field 53), date of the event is "event_dt"
ftg..	Drugs	Metformin	Metformin + Linagliptin	Patients included	Patient included, date of the event is "event_dt"	Patient included if the first event was after the UK Biobank recruitment date (Field 53), date of the event is "event_dt"
ftg2.	Drugs	Metformin	Linagliptin/Metformin Hydrochloride 2.5mg/850mg tablets	Patients included	Patient included, date of the event is "event_dt"	Patient included if the first event was after the UK Biobank recruitment date (Field 53), date of the event is "event_dt"
ftg4.	Drugs	Metformin	Linagliptin/Metformin Hydrochloride 2.5mg/1000mg tablets	Patients included	Patient included, date of the event is "event_dt"	Patient included if the first event was after the UK Biobank recruitment date (Field 53), date of the event is "event_dt"
fti..	Drugs	Metformin	Metformin + Saxagliptin	Patients included	Patient included, date of the event is "event_dt"	Patient included if the first event was after the UK Biobank recruitment date (Field 53), date of the event is "event_dt"
ft4u.	Drugs	Rosiglitazone/Metformin	Rosiglitazone 2mg / Metformin 1000mg tablets	Patients included	Patient included, date of the event is "event_dt"	Patient included if the first event was after the UK Biobank recruitment date (Field 53), date of the event is "event_dt"
ft4v.	Drugs	Rosiglitazone/Metformin	Rosiglitazone 4mg / Metformin 1000mg tablets	Patients included	Patient included, date of the event is "event_dt"	Patient included if the first event was after the UK Biobank recruitment date (Field 53), date of the event is "event_dt"
ft4w.	Drugs	Rosiglitazone/Metformin	Rosiglitazone 2mg / Metformin 500mg tablets	Patients included	Patient included, date of the event is "event_dt"	Patient included if the first event was after the UK Biobank recruitment date (Field 53), date of the event is "event_dt"

ft4x.	Drugs	Rosiglitazone/Metformin	Rosiglitazone 1mg / Metformin 500mg tablets	Patients included	Patient included, date of the event is "event_dt"	Patient included if the first event was after the UK Biobank recruitment date (Field 53), date of the event is "event_dt"
ft5..	Drugs	Pioglitazone		Patients included	Patient included, date of the event is "event_dt"	Patient included if the first event was after the UK Biobank recruitment date (Field 53), date of the event is "event_dt"
ft5x.	Drugs	Pioglitazone	45mg tablets	Patients included	Patient included, date of the event is "event_dt"	Patient included if the first event was after the UK Biobank recruitment date (Field 53), date of the event is "event_dt"
ft5y.	Drugs	Pioglitazone	30mg tablets	Patients included	Patient included, date of the event is "event_dt"	Patient included if the first event was after the UK Biobank recruitment date (Field 53), date of the event is "event_dt"
ft5z.	Drugs	Pioglitazone	15mg tablets	Patients included	Patient included, date of the event is "event_dt"	Patient included if the first event was after the UK Biobank recruitment date (Field 53), date of the event is "event_dt"
ft7..	Drugs	Pioglitazone	Metformin + Pioglitazone	Patients included	Patient included, date of the event is "event_dt"	Patient included if the first event was after the UK Biobank recruitment date (Field 53), date of the event is "event_dt"
ft7z.	Drugs	Pioglitazone	Metformin 850mg/Pioglitazone 15mg tablets	Patients included	Patient included, date of the event is "event_dt"	Patient included if the first event was after the UK Biobank recruitment date (Field 53), date of the event is "event_dt"
ft1..	Drugs	Acarbose		Patients included	Patient included, date of the event is "event_dt"	Patient included if the first event was after the UK Biobank recruitment date (Field 53), date of the event is "event_dt"
ft11.	Drugs	Acarbose	50mg tablets	Patients included	Patient included, date of the event is "event_dt"	Patient included if the first event was after the UK Biobank recruitment date (Field 53), date of the event is "event_dt"
ft12.	Drugs	Acarbose	100mg tablets	Patients included	Patient included, date of the event is "event_dt"	Patient included if the first event was after the UK Biobank recruitment date (Field 53), date of the event is "event_dt"
ft3..	Drugs	Repaglinide		Patients included	Patient included, date of the event is "event_dt"	Patient included if the first event was after the UK Biobank recruitment date (Field 53), date of the event is "event_dt"
ft31.	Drugs	Repaglinide	0.5mg tablets	Patients included	Patient included, date of the event is "event_dt"	Patient included if the first event was after the UK Biobank recruitment date (Field 53), date of the event is "event_dt"
ft32.	Drugs	Repaglinide	1mg tablets	Patients included	Patient included, date of the event is "event_dt"	Patient included if the first event was after the UK Biobank recruitment date (Field 53), date of the event is "event_dt"
ft33.	Drugs	Repaglinide	2mg tablets	Patients included	Patient included, date of the event is "event_dt"	Patient included if the first event was after the UK Biobank recruitment date (Field 53), date of the event is "event_dt"
f3A..	Drugs	Glimepiride		Patients included	Patient included, date of the event is "event_dt"	Patient included if the first event was after the UK Biobank recruitment date (Field 53), date of the event is "event_dt"
f3A1.	Drugs	Glimepiride	2mg tablets	Patients included	Patient included, date of the event is "event_dt"	Patient included if the first event was after the UK Biobank recruitment date (Field 53), date of the event is "event_dt"
f3A3.	Drugs	Glimepiride	1mg tablets	Patients included	Patient included, date of the event is "event_dt"	Patient included if the first event was after the UK Biobank recruitment date (Field 53), date of the event is "event_dt"
f3A4.	Drugs	Glimepiride	3mg tablets	Patients included	Patient included, date of the event is "event_dt"	Patient included if the first event was after the UK Biobank recruitment date (Field 53), date of the event is "event_dt"
f3A5.	Drugs	Glimepiride	4mg tablets	Patients included	Patient included, date of the event is "event_dt"	Patient included if the first event was after the UK Biobank recruitment date (Field 53), date of the event is "event_dt"
f362.	Drugs	Glibenese	*5mg tablets	Patients included	Patient included, date of the event is "event_dt"	Patient included if the first event was after the UK Biobank recruitment date (Field 53), date of the event is "event_dt"
f363.	Drugs	Miniodiab	*2.5mg tablets	Patients included	Patient included, date of the event is "event_dt"	Patient included if the first event was after the UK Biobank recruitment date (Field 53), date of the event is "event_dt"
f364.	Drugs	Miniodiab	5mg tablets	Patients included	Patient included, date of the event is "event_dt"	Patient included if the first event was after the UK Biobank recruitment date (Field 53), date of the event is "event_dt"
f37..	Drugs	Gliquidone		Patients included	Patient included, date of the event is "event_dt"	Patient included if the first event was after the UK Biobank recruitment date (Field 53), date of the event is "event_dt"
f37z.	Drugs	Gliquidone	*30mg tablets	Patients included	Patient included, date of the event is "event_dt"	Patient included if the first event was after the UK Biobank recruitment date (Field 53), date of the event is "event_dt"
f371.	Drugs	Glurenorm	*30mg tablets	Patients included	Patient included, date of the event is "event_dt"	Patient included if the first event was after the UK Biobank recruitment date (Field 53), date of the event is "event_dt"
f391.	Drugs	Tolanase	100mg tablets	Patients included	Patient included, date of the event is "event_dt"	Patient included if the first event was after the UK Biobank recruitment date (Field 53), date of the event is "event_dt"
f392.	Drugs	Tolanase	250mg tablets	Patients included	Patient included, date of the event is "event_dt"	Patient included if the first event was after the UK Biobank recruitment date (Field 53), date of the event is "event_dt"
f3a2.	Drugs	Glyconon	500mg tablets	Patients included	Patient included, date of the event is "event_dt"	Patient included if the first event was after the UK Biobank recruitment date (Field 53), date of the event is "event_dt"
f3a4.	Drugs	Rastinon	*500mg tablets	Patients included	Patient included, date of the event is "event_dt"	Patient included if the first event was after the UK Biobank recruitment date (Field 53), date of the event is "event_dt"
f413.	Drugs	Orabet	*500mg tablets	Patients included	Patient included, date of the event is "event_dt"	Patient included if the first event was after the UK Biobank recruitment date (Field 53), date of the event is "event_dt"

f414.	Drugs	Orabet	*850mg tablets	Patients included	Patient included, date of the event is "event_dt"	Patient included if the first event was after the UK Biobank recruitment date (Field 53), date of the event is "event_dt"
f323.	Drugs	Diabinese	*100mg tablets	Patients included	Patient included, date of the event is "event_dt"	Patient included if the first event was after the UK Biobank recruitment date (Field 53), date of the event is "event_dt"
f324.	Drugs	Diabinese	*250mg tablets	Patients included	Patient included, date of the event is "event_dt"	Patient included if the first event was after the UK Biobank recruitment date (Field 53), date of the event is "event_dt"
f325.	Drugs	Glymese	250mg tablets	Patients included	Patient included, date of the event is "event_dt"	Patient included if the first event was after the UK Biobank recruitment date (Field 53), date of the event is "event_dt"
f333.	Drugs	Daonil	*5mg tablets	Patients included	Patient included, date of the event is "event_dt"	Patient included if the first event was after the UK Biobank recruitment date (Field 53), date of the event is "event_dt"
f33b.	Drugs	Daonil	*CP 5mg tablets	Patients included	Patient included, date of the event is "event_dt"	Patient included if the first event was after the UK Biobank recruitment date (Field 53), date of the event is "event_dt"
f334.	Drugs	Semi-Daonil	*2.5mg tablets	Patients included	Patient included, date of the event is "event_dt"	Patient included if the first event was after the UK Biobank recruitment date (Field 53), date of the event is "event_dt"
f33c.	Drugs	Semi-Daonil	*CP 2.5mg tablets	Patients included	Patient included, date of the event is "event_dt"	Patient included if the first event was after the UK Biobank recruitment date (Field 53), date of the event is "event_dt"
f335.	Drugs	Euglucon	*2.5mg tablets	Patients included	Patient included, date of the event is "event_dt"	Patient included if the first event was after the UK Biobank recruitment date (Field 53), date of the event is "event_dt"
f336.	Drugs	Euglucon	*5mg tablets	Patients included	Patient included, date of the event is "event_dt"	Patient included if the first event was after the UK Biobank recruitment date (Field 53), date of the event is "event_dt"
f339.	Drugs	Malix	*2.5mg tablets	Patients included	Patient included, date of the event is "event_dt"	Patient included if the first event was after the UK Biobank recruitment date (Field 53), date of the event is "event_dt"
f33a.	Drugs	Malix	*5mg tablets	Patients included	Patient included, date of the event is "event_dt"	Patient included if the first event was after the UK Biobank recruitment date (Field 53), date of the event is "event_dt"
f33d.	Drugs	Diabetamide	*2.5mg tablets	Patients included	Patient included, date of the event is "event_dt"	Patient included if the first event was after the UK Biobank recruitment date (Field 53), date of the event is "event_dt"
f33e.	Drugs	Diabetamide	*5mg tablets	Patients included	Patient included, date of the event is "event_dt"	Patient included if the first event was after the UK Biobank recruitment date (Field 53), date of the event is "event_dt"
f33f.	Drugs	Calabren	*2.5mg tablets	Patients included	Patient included, date of the event is "event_dt"	Patient included if the first event was after the UK Biobank recruitment date (Field 53), date of the event is "event_dt"
f33g.	Drugs	Calabren	* 5mg tablets	Patients included	Patient included, date of the event is "event_dt"	Patient included if the first event was after the UK Biobank recruitment date (Field 53), date of the event is "event_dt"
f351.	Drugs	Diamicron	80mg tablets	Patients included	Patient included, date of the event is "event_dt"	Patient included if the first event was after the UK Biobank recruitment date (Field 53), date of the event is "event_dt"
f354.	Drugs	Diamicron	MR 30mg m/r tablets	Patients included	Patient included, date of the event is "event_dt"	Patient included if the first event was after the UK Biobank recruitment date (Field 53), date of the event is "event_dt"
ft51.	Drugs	Actos	15mg tablets	Patients included	Patient included, date of the event is "event_dt"	Patient included if the first event was after the UK Biobank recruitment date (Field 53), date of the event is "event_dt"
ft52.	Drugs	Actos	30mg tablets	Patients included	Patient included, date of the event is "event_dt"	Patient included if the first event was after the UK Biobank recruitment date (Field 53), date of the event is "event_dt"
ft53.	Drugs	Actos	45mg tablets	Patients included	Patient included, date of the event is "event_dt"	Patient included if the first event was after the UK Biobank recruitment date (Field 53), date of the event is "event_dt"
ft13.	Drugs	Glucobay	50 tablets	Patients included	Patient included, date of the event is "event_dt"	Patient included if the first event was after the UK Biobank recruitment date (Field 53), date of the event is "event_dt"
ft14.	Drugs	Glucobay	100 tablets	Patients included	Patient included, date of the event is "event_dt"	Patient included if the first event was after the UK Biobank recruitment date (Field 53), date of the event is "event_dt"
ft34.	Drugs	Novonorm	*0.5mg tablets	Patients included	Patient included, date of the event is "event_dt"	Patient included if the first event was after the UK Biobank recruitment date (Field 53), date of the event is "event_dt"
ft35.	Drugs	Novonorm	*1mg tablets	Patients included	Patient included, date of the event is "event_dt"	Patient included if the first event was after the UK Biobank recruitment date (Field 53), date of the event is "event_dt"
ft36.	Drugs	Novonorm	*2mg tablets	Patients included	Patient included, date of the event is "event_dt"	Patient included if the first event was after the UK Biobank recruitment date (Field 53), date of the event is "event_dt"

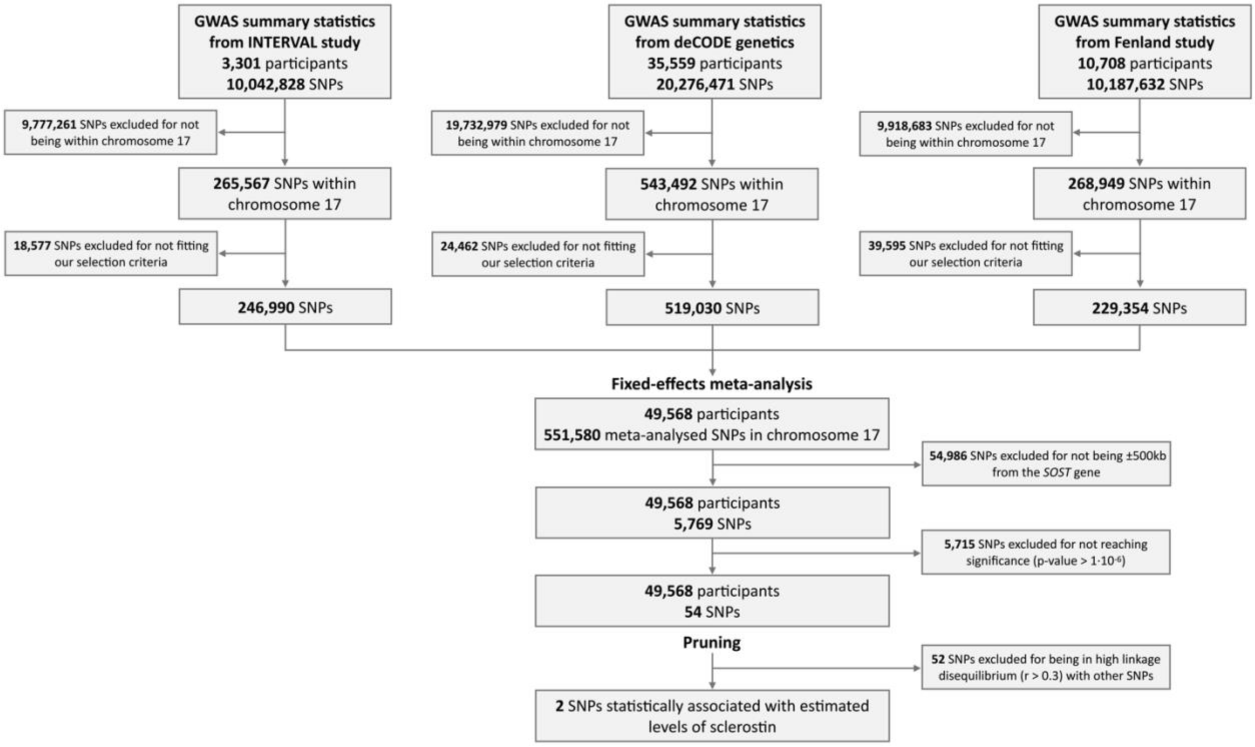

Supplementary Fig. 1: Flow chart of sclerostin meta-analysis and instrument selection.

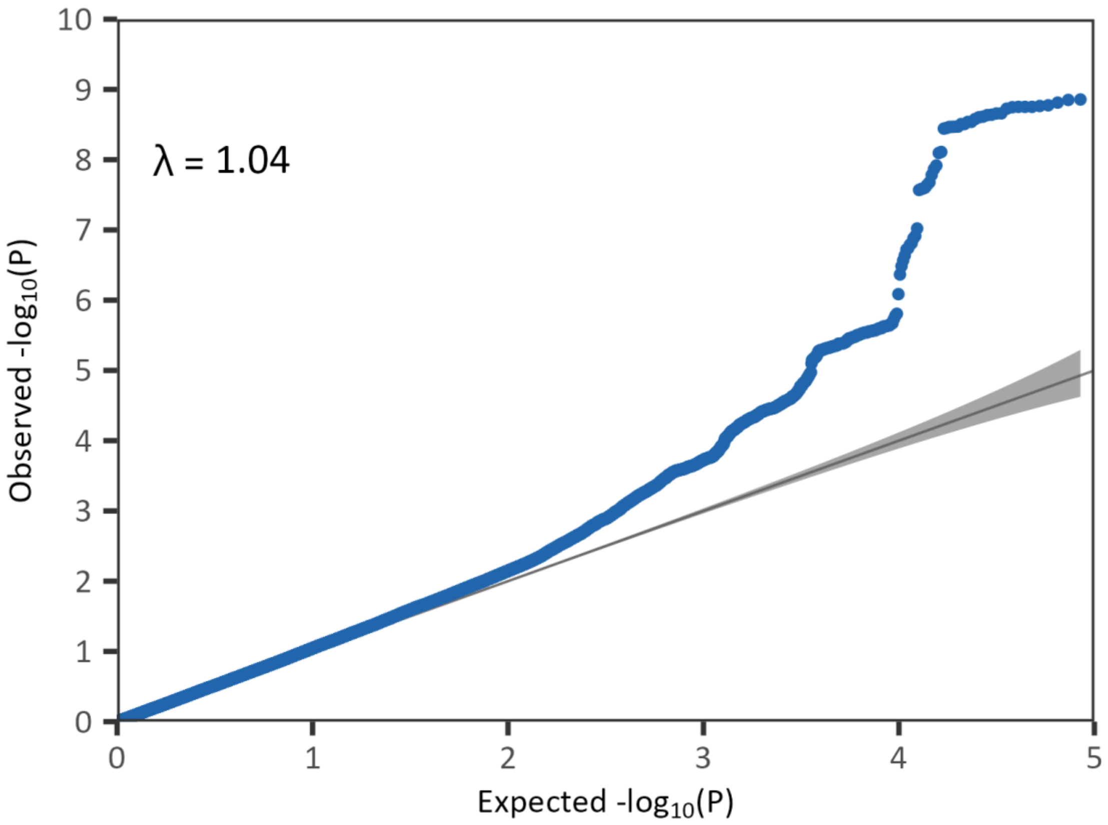

Supplementary Fig. 2: QQ plot of the fixed-effects meta-analysis results.

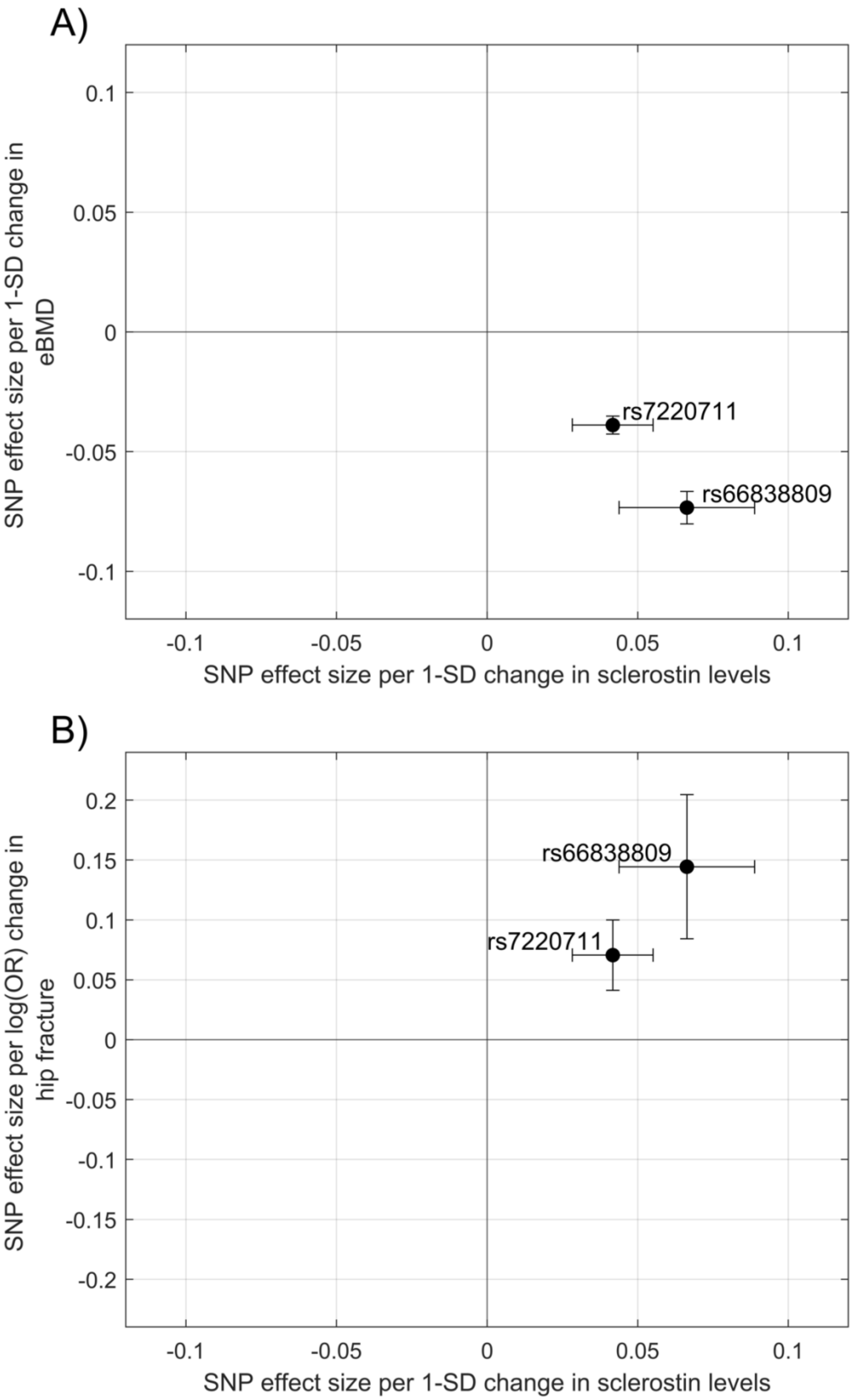

Supplementary Fig. 3: SNP effects on the positive control outcomes against SNP effects on the exposure. All SNPs with negative effects on the exposure are shown to be positive, with the sign of the effect on the outcome flipped. Error bars indicate the 95% confidence interval. **(A)** The vertical axis is the SNP effect size per 1-SD change in eBMD and, **(B)** the vertical axis is the SNP effect size for hip fracture.

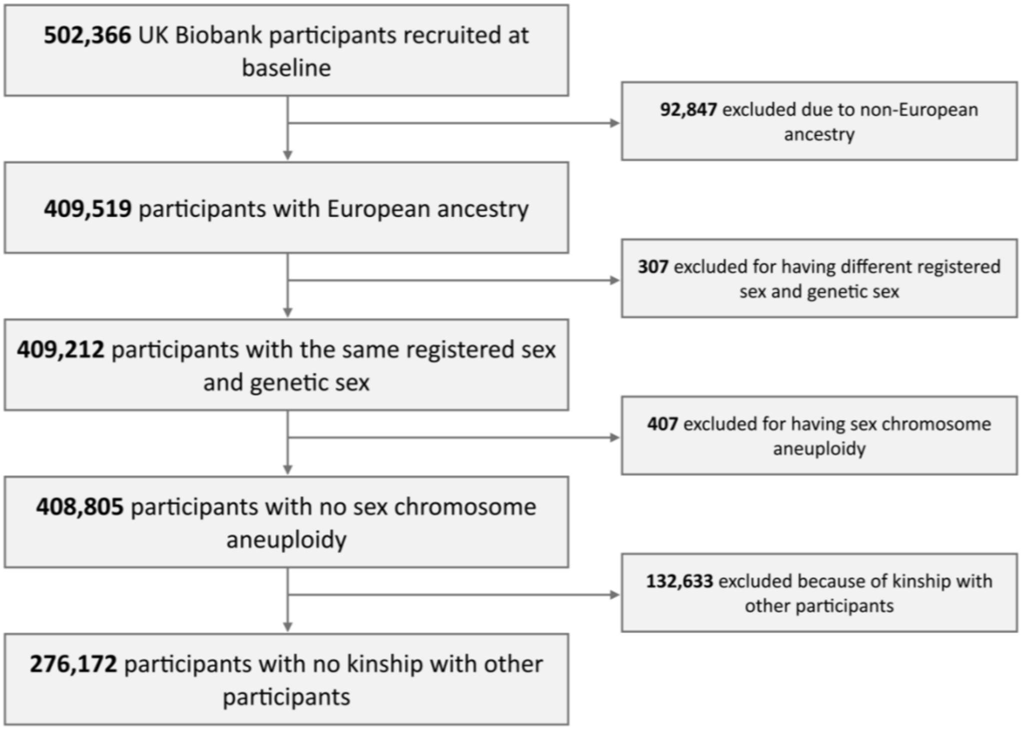

Supplementary Fig 4: Flow chart of the selection process of participants in the UK Biobank analysis.

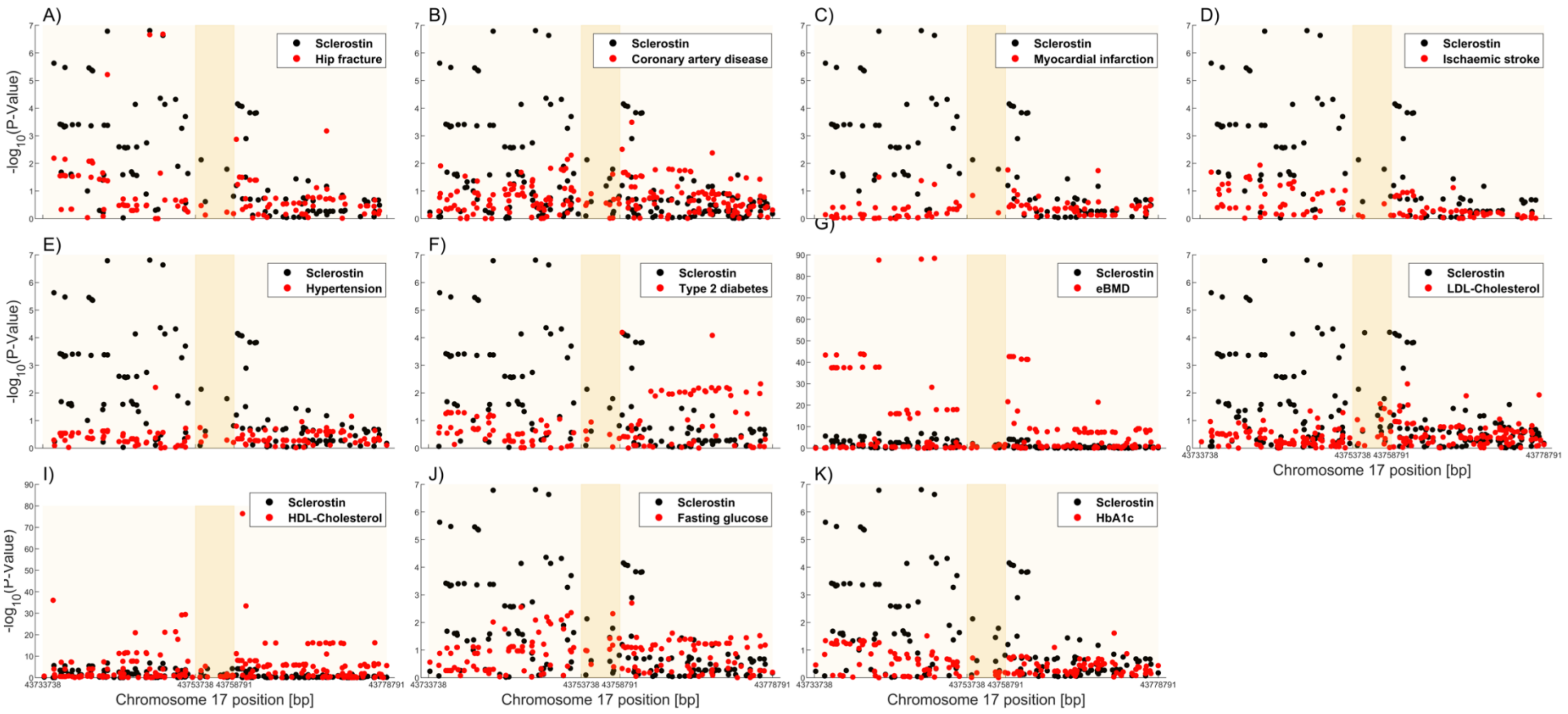

Supplementary Fig. 5: Colocalization analysis within ±20kb SOST gene. In dark yellow is the SOST gene. In light yellow is the area ±20kbp from the beginning and end of the SOST gene. **(A)** Regional plot for hip fracture, **(B)** Regional plot for coronary artery disease. **(C)** Regional plot for myocardial infarction. **(D)** Regional plot for ischaemic stroke. **(E)** Regional plot for hypertension. **(F)** Regional plot for type II diabetes mellitus. **(G)** Regional plot for estimated heel bone mineral density (eBMD). **(H)** Regional plot for LDL Cholesterol. **(I)** Regional plot for HDL Cholesterol. **(J)** Regional plot for fasting glucose. **(I)** Regional plot for HbA1c.

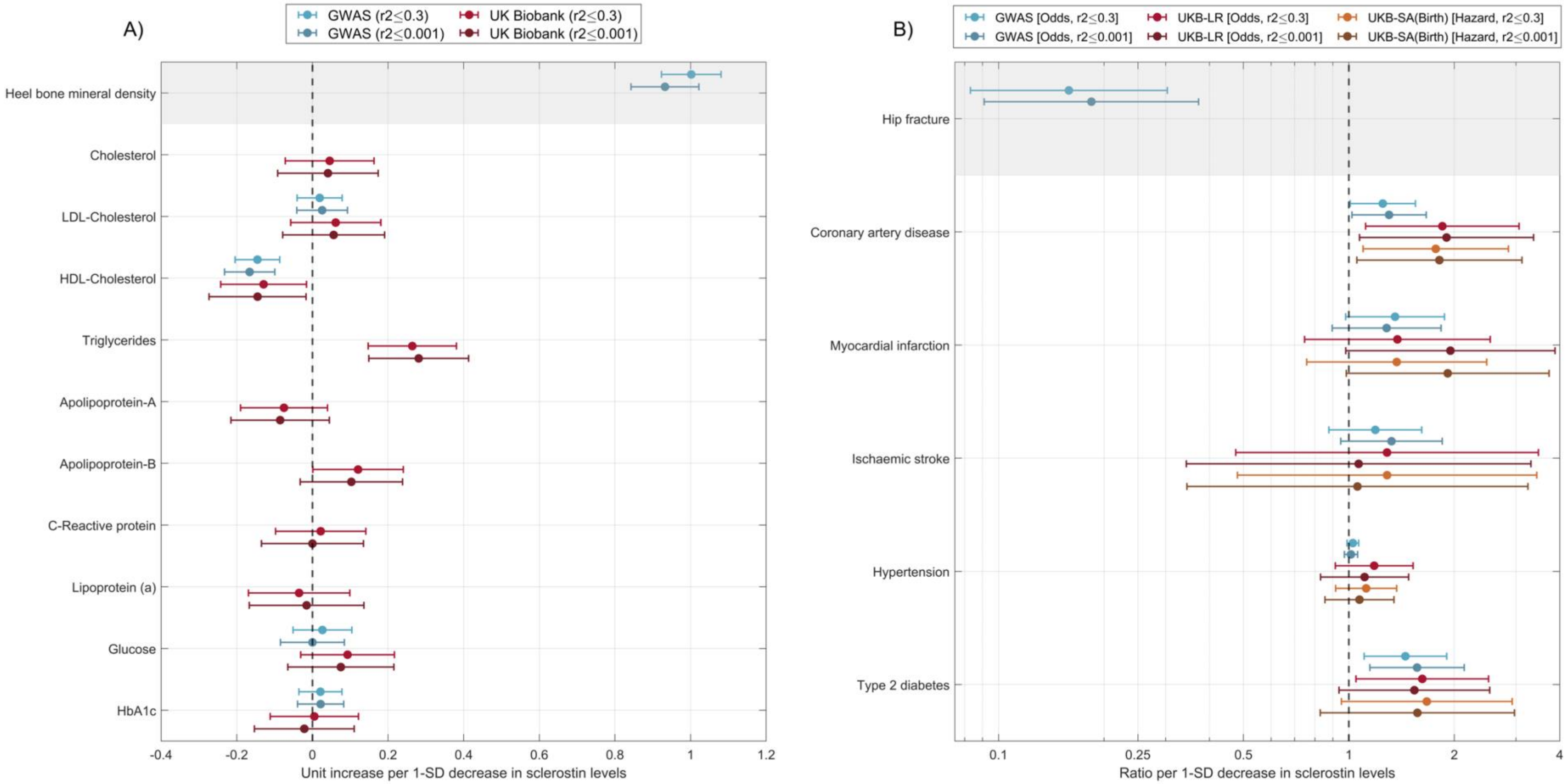

Supplementary Fig. 6: Stepwise pruning sensitivity analysis results using an $r^2 \leq 0.001$. Effect sizes were calculated using the Wald Ratio method. Results from the main analysis are represented using light colours, whereas those obtained using an $r^2 \leq 0.001$ are shown using dark colours. Blue indicates the results using published GWAS summary statistics as the outcome; maroon the results obtained using a linear regression (for continuous outcomes) and logistic regression (for categorical outcomes) on the UK Biobank outcomes; and orange indicates the results using cox regression for UK Biobank survival outcomes. Error bars indicate the 95% confidence interval. **(A)** The horizontal axis shows the unit increase (95% CI) per 1 SD decrease in sclerostin levels. GWAS results for LDL and HDL-Cholesterol are in mg/dL, mmol/L for GWAS results of fasting glucose, % change for HbA1c and SD increase for the other outcomes. **(B)** The horizontal axis shows the odds/hazard ratio (95% CI) per 1 SD decrease in sclerostin levels. Note: UKB-LR = UK Biobank logistic regression, UKB-SA = UK Biobank survival analysis. Stepwise pruning sensitivity analysis results using an $r^2 \leq 0.001$.

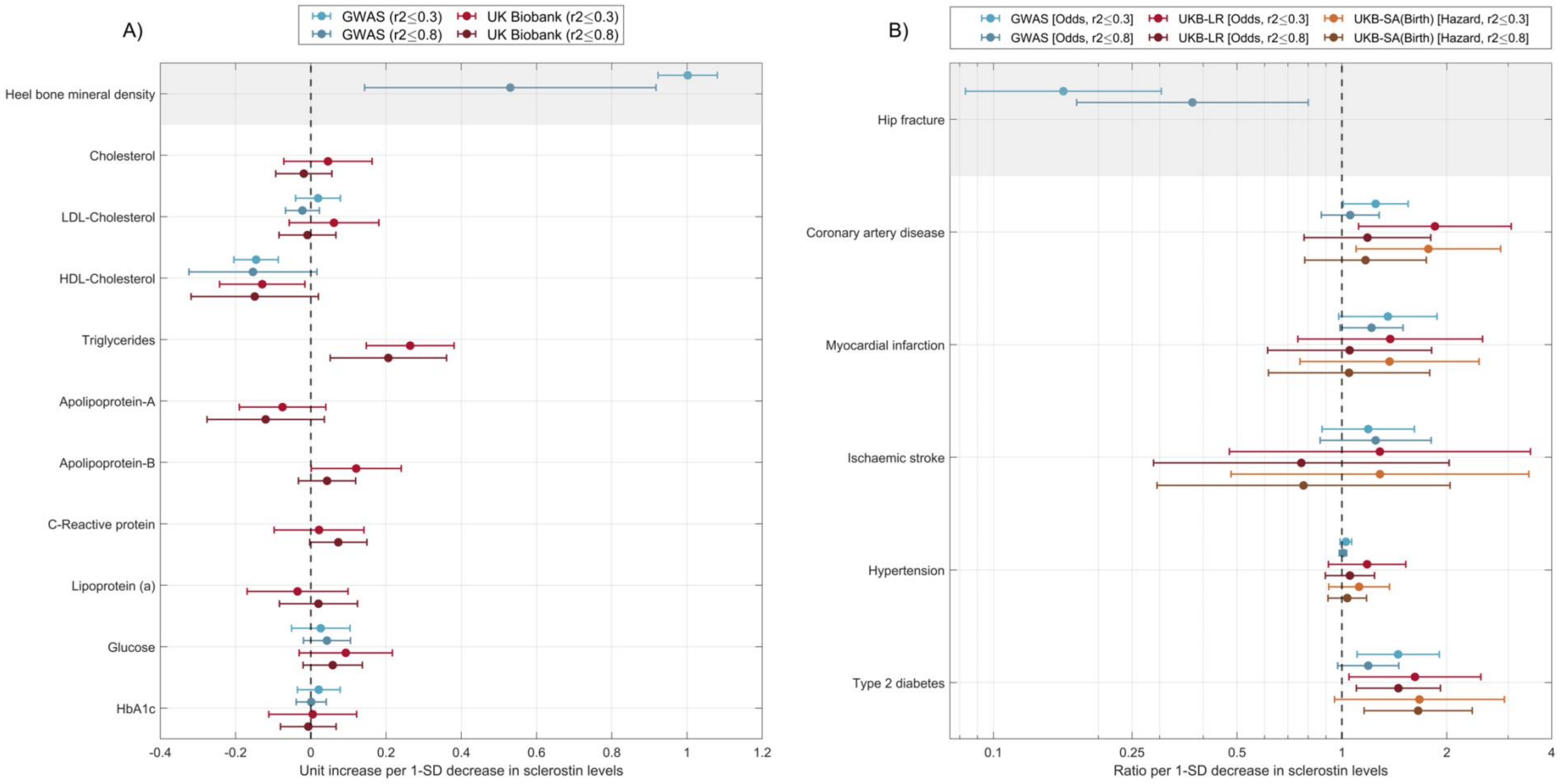

Supplementary Fig. 7: Stepwise pruning sensitivity analysis results using an $r^2 \leq 0.8$. Effect sizes were calculated using the generalised inverse variance weighted method. Results from the main analysis are represented using light colours, whereas those obtained using an $r^2 \leq 0.8$ are shown using dark colours. Blue indicates the results using published GWAS summary statistics as the outcome; maroon the results obtained using a linear regression (for continuous outcomes) and logistic regression (for categorical outcomes) on the UK Biobank outcomes; and orange indicates the results using cox regression for UK Biobank survival outcomes. Error bars indicate the 95% confidence interval. **(A)** The horizontal axis shows the unit increase (95% CI) per 1 SD decrease in sclerostin levels. GWAS results for LDL and HDL-Cholesterol are in mg/dL, mmol/L for GWAS results of fasting glucose, % change for HbA1c and SD increase for the other outcomes. **(B)** The horizontal axis shows the odds/hazard ratio (95% CI) per 1 SD decrease in sclerostin levels. Note: UKB-LR = UK Biobank logistic regression, UKB-SA = UK Biobank survival analysis.

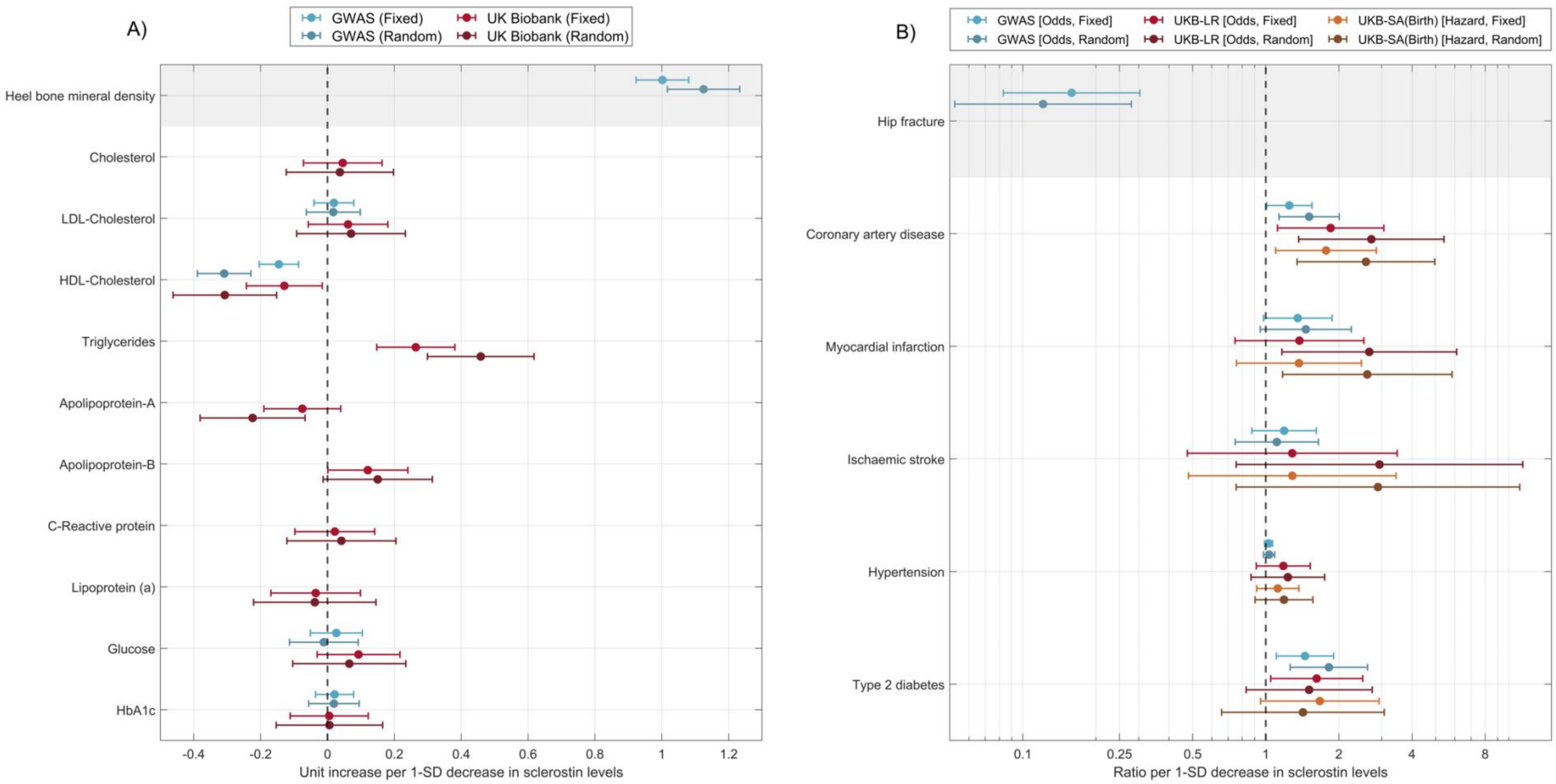

Supplementary Fig. 8: Random-effects sensitivity analysis results. Effect sizes were calculated using the generalised inverse variance weighted method. Results from the main analysis are represented using light colours, whereas random-effects results are shown using dark colours. Blue indicates the results using published GWAS summary statistics as the outcome; maroon the results obtained using a linear regression (for continuous outcomes) and logistic regression (for categorical outcomes) on the UK Biobank outcomes; and orange indicates the results using cox regression for UK Biobank survival outcomes. Error bars indicate the 95% confidence interval. **(A)** The horizontal axis shows the unit increase (95% CI) per 1 SD decrease in sclerostin levels. GWAS results for LDL and HDL-Cholesterol are in mg/dL, mmol/L for GWAS results of fasting glucose, % change for HbA1c and SD increase for the other outcomes. **(B)** The horizontal axis shows the odds/hazard ratio (95% CI) per 1 SD decrease in sclerostin levels. Note: UKB-LR = UK Biobank logistic regression, UKB-SA = UK Biobank survival analysis.

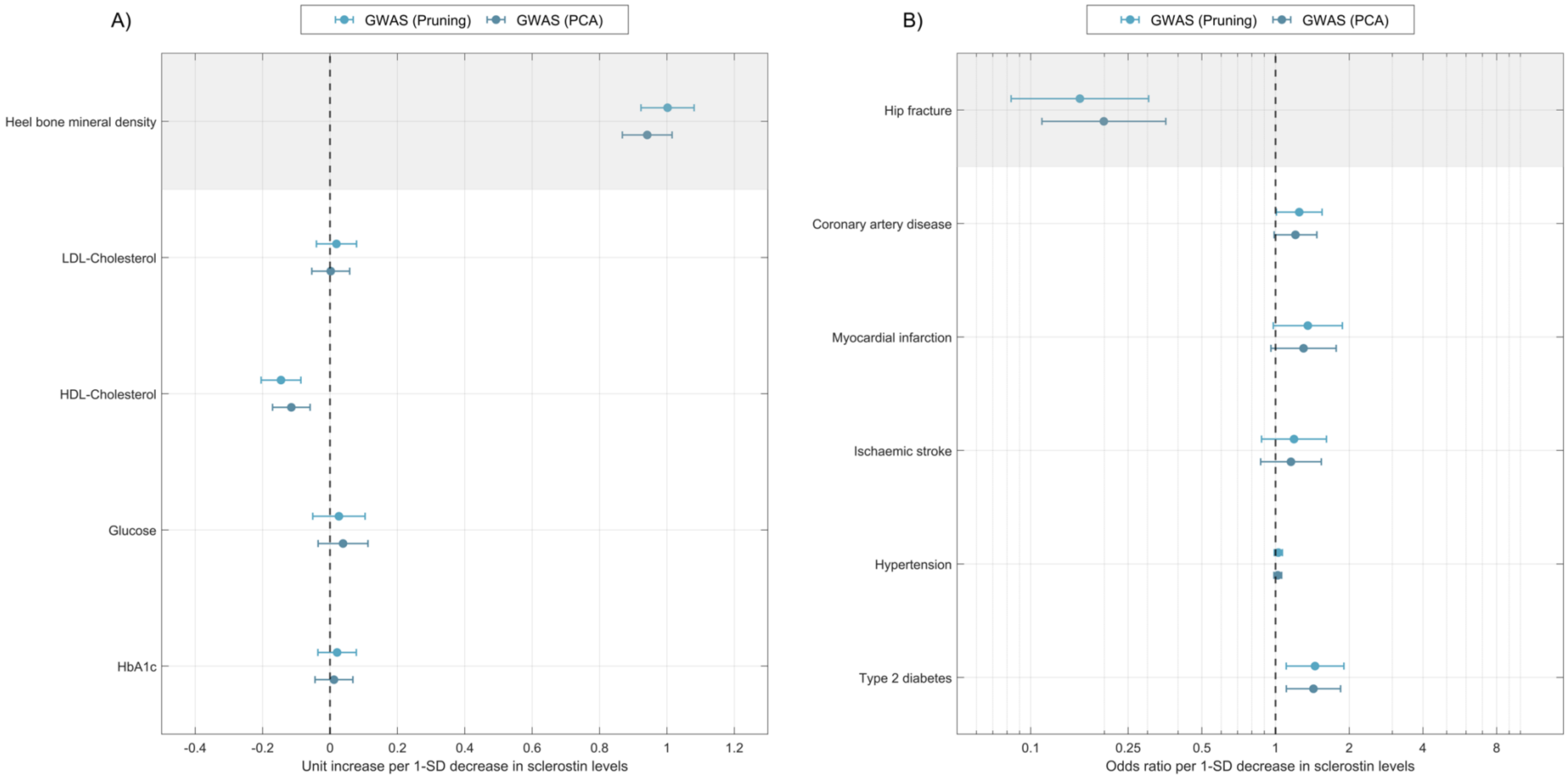

Supplementary Fig. 9: Principal components sensitivity analysis results. Results from the main analysis are shown using light colours, whereas principal components results are shown using dark colours. Error bars indicate the 95% confidence interval. **(A)** The horizontal axis shows the unit increase (95% CI) per 1 SD decrease in sclerostin levels. GWAS results for LDL and HDL-Cholesterol are in mg/dL, mmol/L for GWAS results of fasting glucose, % change for HbA1c and SD increase for the other outcomes. **(B)** The horizontal axis shows the odds/hazard ratio (95% CI) per 1 SD decrease in sclerostin levels. Note: UKB-LR = UK Biobank logistic regression, UKB-SA = UK Biobank survival analysis.

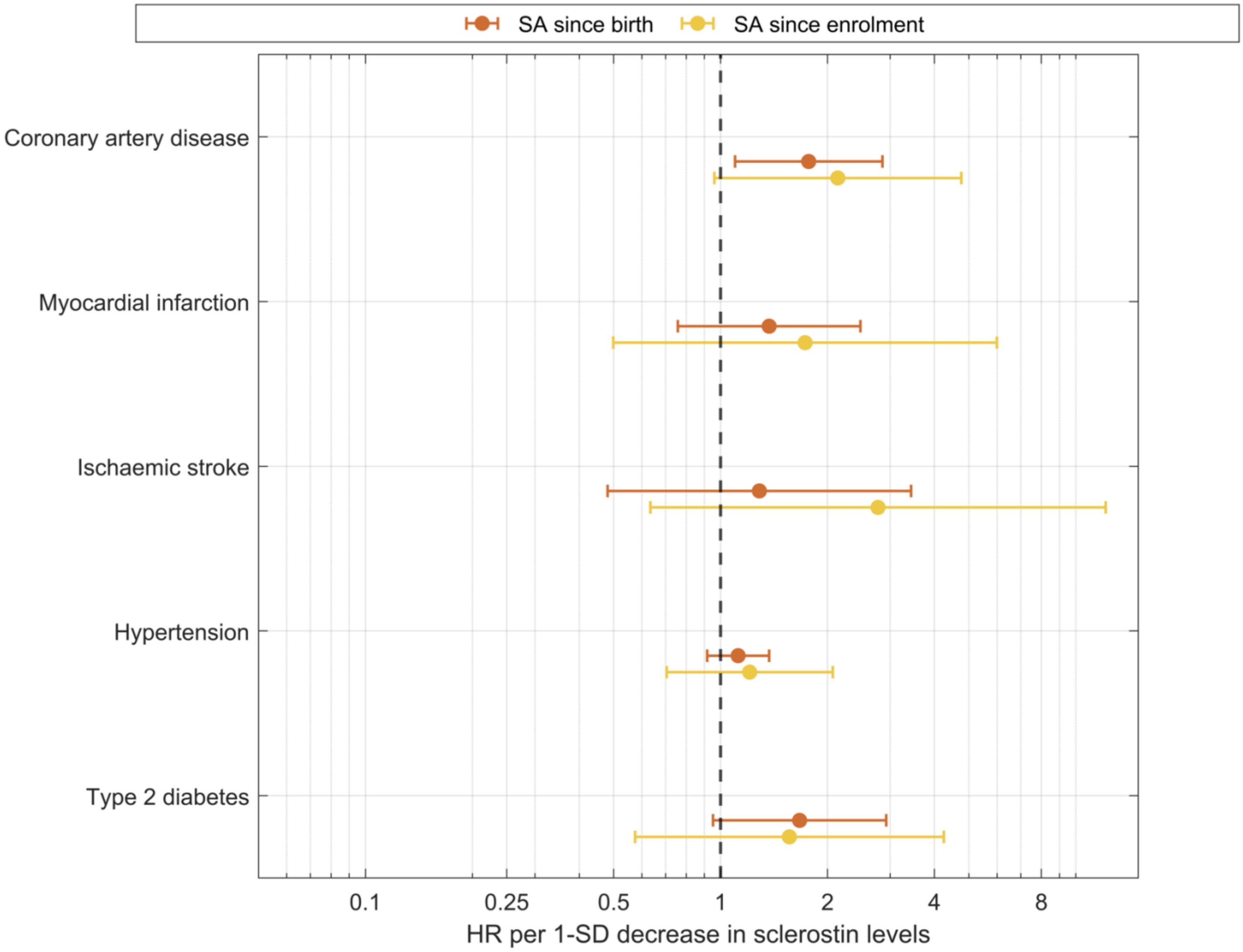

Supplementary Fig. 10: Survival analysis since UK Biobank enrolment sensitivity analysis results. Effect sizes were calculated using the generalised inverse variance weighted method. Main analysis results (survival outcomes since birth date) are shown in orange, whereas results from the sensitivity analysis (survival outcomes since UK Biobank enrolment) are shown in yellow. The horizontal axis shows the hazard ratio (HR) (95% CI) per 1 SD decrease in sclerostin levels. Error bars indicate the 95% confidence interval. Note: SA = Survival analysis.
