# Supplementary material for: Effect of genetically predicted sclerostin on cardiovascular biomarkers, risk factors, and disease outcomes
Source: Nat Commun. 2024 Nov 13;15:9832. doi: 10.1038/s41467-024-53623-5 (PMC11561231; doi:10.1038/s41467-024-53623-5)
Supplement: Supplementary file 2 — Description of Additional Supplementary Files [file 41467_2024_53623_MOESM2_ESM.pdf]

Supplementary Data 1.

Results from the fixed-effects and random-effect meta-analysis.
